# Gene Expression Analysis in Ovarian Cancer – Faults and Hints from DNA Microarray Study

**DOI:** 10.3389/fonc.2014.00006

**Published:** 2014-01-28

**Authors:** Katarzyna Marta Lisowska, Magdalena Olbryt, Volha Dudaladava, Jolanta Pamuła-Piłat, Katarzyna Kujawa, Ewa Grzybowska, Michał Jarząb, Sebastian Student, Iwona Krystyna Rzepecka, Barbara Jarząb, Jolanta Kupryjańczyk

**Affiliations:** ^1^Center for Translational Research and Molecular Biology of Cancer, Maria Skłodowska-Curie Memorial Cancer Center and Institute of Oncology, Gliwice, Poland; ^2^Department of Medical Biology and Genetics, Grodno State Medical University, Grodno, Belarus; ^3^Faculty of Automated Control, Electronics and Computer Science, Silesian University of Technology, Gliwice, Poland; ^4^Department of Pathology, Maria Skłodowska-Curie Memorial Cancer Center and Institute of Oncology, Warsaw, Poland; ^5^Department of Nuclear Medicine and Oncological Endocrinology, Maria Skłodowska-Curie Memorial Cancer Center and Institute of Oncology, Gliwice, Poland

**Keywords:** epithelial ovarian cancer, gene expression profiling, oligonucleotide microarrays, tumor histology, survival time, molecular markers, genomic medicine, *CLASP1*

## Abstract

The introduction of microarray techniques to cancer research brought great expectations for finding biomarkers that would improve patients’ treatment; however, the results of such studies are poorly reproducible and critical analyses of these methods are rare. In this study, we examined global gene expression in 97 ovarian cancer samples. Also, validation of results by quantitative RT-PCR was performed on 30 additional ovarian cancer samples. We carried out a number of systematic analyses in relation to several defined clinicopathological features. The main goal of our study was to delineate the molecular background of ovarian cancer chemoresistance and find biomarkers suitable for prediction of patients’ prognosis. We found that histological tumor type was the major source of variability in genes expression, except for serous and undifferentiated tumors that showed nearly identical profiles. Analysis of clinical endpoints [tumor response to chemotherapy, overall survival, disease-free survival (DFS)] brought results that were not confirmed by validation either on the same group or on the independent group of patients. *CLASP1* was the only gene that was found to be important for DFS in the independent group, whereas in the preceding experiments it showed associations with other clinical endpoints and with *BRCA1* gene mutation; thus, it may be worthy of further testing. Our results confirm that histological tumor type may be a strong confounding factor and we conclude that gene expression studies of ovarian carcinomas should be performed on histologically homogeneous groups. Among the reasons of poor reproducibility of statistical results may be the fact that despite relatively large patients’ group, in some analyses one has to compare small and unequal classes of samples. In addition, arbitrarily performed division of samples into classes compared may not always reflect their true biological diversity. And finally, we think that clinical endpoints of the tumor probably depend on subtle changes in many and, possibly, alternative molecular pathways, and such changes may be difficult to demonstrate.

## Introduction

Since the report describing the use of microarray technique in cancer research by Golub et al. (1), great expectations were born concerning better cancer classification, discovery of new molecular markers and finally, individualization of patient’s treatment. Disappointingly, after 15 years of research, most potential genomic medicine tools remain at experimental stage and their clinical validity and utility has not been established (2). Although some new biomarkers have emerged from the microarray studies, very few were introduced into clinical practice [e.g., Ref. (3–18); reviewed recently in Ref. (19)]. For ovarian cancer, only one single new biomarker, HE4 was cleared by FDA and one multi-marker test OVA1 (Vermillion Inc.) was developed. HE4, similarly to CA125, is accepted for monitoring and recurrence of the disease, while OVA1 is approved for women with undefined ovarian mass, to assess whether they should be referred to the oncology specialist. None of these biomarkers are suitable for ovarian cancer screening.

We performed a microarray study, which was carefully designed and based on relatively large collection of well characterized clinical samples. Our primary goal was to dissect the molecular background of tumor chemoresistance and to find molecular markers suitable for prediction of therapy failure as well as patient’s outcome (prognosis). In addition, we performed a number of systematic analyses of gene expression patterns related to several defined clinicopathological and molecular features. However, in most comparisons we obtained low numbers of statistically significant genes, majority of which were not validated by real-time RT-PCR. Nonetheless, our results allowed for some considerations concerning biology of ovarian cancer and brought some important hints concerning the analysis of expression data.

## Materials and Methods

### Clinical samples

Surgical samples of ovarian cancer were obtained during primary surgery, then snap-frozen in liquid nitrogen and stored at −80°C. Only samples from patients without neo-adjuvant chemotherapy were used. The tissue samples were collected at the Maria Skłodowska-Curie Memorial Cancer Center and Institute of Oncology in Warsaw, Poland. Altogether, we analyzed 97 ovarian cancer specimens: 71 serous, 11 endometrioid, 9 clear cell, and 6 undifferentiated [classified according to the criteria of the World Health Organization (20)]. The tumors were graded in a four-grade scale, according to the criteria given in Ref. (21).

The majority of clinical analyses were performed on a group of 72 samples (68 serous and 4 undifferentiated) with complete clinical data (Table 1). Of those, 32 patients were treated with platinum/cyclophosphamide, while 40 patients were treated with taxane/platinum regimen. Since it was not possible to obtain a group uniform as to residual tumor size, we chose samples from patients in whom the residual tumor apparently did not influence treatment results, e.g., sensitivity to chemotherapy in a patient with large residual tumor or progression in a patient with small residual tumor. Detection of hereditary mutations in BRCA1 gene was done according to Ref. (22). For external validation of the selected genes, we used an independent set of 30 serous ovarian cancers. Detailed criteria of evaluation of the tumors and clinical endpoints were given previously (23).

**Table 1:** **Characteristics of the group of patients and tumor samples**.

Characteristics	Numbers of samples (*n*)							
	Status	*n*	Status	*n*	Status	*n*	Status	*n*
Histology	Serous	71	Endometrioid	11	Clear cell	9	Undifferentiated	6
CHT-response	CR	48	PR	14	SD	3	P	7
Platinum-sensitivity	Highly sensitive	12	Moderately sensitive	27	Resistant	33	
FIGO stage	FIGO II	3	FIGO III	59	FIGO IV	10	
Tumor grade	G2	9	G3	49	G4	19	
Residual tumor	R0	15	R1	36	R2	21	
BRCA1 mutation	Mutation	19	No mutation	53			

*R0, residual tumor less than 1 cm; R1, residual tumor between 1 and 5 cm; R2, residual tumor larger than 5 cm. Chemotherapy (CHT) response described as clinical status of the patient after first line treatment: CR, complete response; PR, partial response; SD, stable disease; P, progression. Platinum-sensitivity: tumors were classified as highly sensitive when DFS was >732 days, moderately sensitive when 180 > DFS > 732 and resistant when DFS <180 days*.

### RNA isolation

Total RNA was isolated from three to five sections (20 μm thick) of frozen tumor using RNeasy Mini Kit (Qiagen) with simultaneous on-column DNase I digestion. RNA purity and concentration were estimated with ND-1000 spectrophotometer (NanoDrop Technologies). RNA quality was assessed using Agilent platform: RNA 6000 Nano LabChip Kit, RNA Integrity Number software, and the Agilent 2100 Bioanalyzer (Agilent Technologies). The samples with RIN values above 7 (full range: 0–10) were accepted for further processing.

### Oligonucleotide microarrays

We used HG U133 Plus 2.0 Gene Chip oligonucleotide arrays (Affymetrix). The hybridizations were carried out as described in Ref. (24). Briefly: total RNA (8 μg) was used for synthesis of double stranded cDNA. Biotinylated cRNA was synthesized with the BioArray High Yield RNA Transcript Labeling Kit (Enzo Diagnostics). Both cDNA and cRNA were purified with Gene Chip Sample Cleanup Module (Affymetrix). cRNA (16 μg) was fragmented and hybridized to the microarray for 16 h at 45°C. The microarrays were stained, washed, and subsequently scanned with GeneChip Scanner 3000 (Affymetrix). Data were acquired using GCOS 1.2 software (Affymetrix). The preprocessing was performed by Robust Multi-array Analysis (RMA, Bioconductor).

### Reverse-transcription and quantitative PCR

Half a microgram of total RNA was taken for cDNA synthesis using Omniscript RT Kit (Qiagen), random primers (4 μM, Sigma-Aldrich), oligo(dT) primer (1 μM, QBiogene Inc.), and RNase inhibitor (10 U, Fermentas). The reaction was performed in 20 μl of total volume, according to manufacturer’s protocol, using thermocycler UNO II (Biometra). The cDNA was diluted 10-fold and a 5-μl aliquot was taken for real-time PCR performed using Taqman 2× PCR Master Mix (Roche), Exiqon probe (100 nM), and appropriate primers (200 nM each; Data Sheet 1 in Supplementary Material) designed using dedicated software from the Roche web site. The reaction was carried out using ABI PRISM7700 Sequence Detection System (Applied Biosystems) and the following thermal conditions: 2 min at 50°C, 10 min at 95°C, 40 cycles of 15 s at 95°C, 1 min at 60°C, and 1 min at 72°C. The experiments were performed in triplicates. The relative amount of cDNA was calculated using comparative Δ*C*_t_ method. Δ*C*_t_ values of the samples of interest were compared with a calibrator (RNA of known concentration pooled from several samples). The *C*_t_ values of both the calibrator and the samples of interest were normalized to the expression of three control genes, *ATP6V1*, *HADHA*, and *UBE2D2*.

### Methods of data analysis

Gene expression comparisons by Welch *t*-test were performed using GeneSpring 7.2 software (Agilent), with non-corrected threshold of *p*-value <0.001. False Discovery Rate (FDR) was estimated by Benjamini–Hochberg algorithm. Two-way analysis of variance (ANOVA), with random variance assumption and global testing were carried out by procedures implemented in BRB Array (developed by Richard Simon and Amy Peng Lam; available on the National Cancer Institute website). Class prediction procedure was carried out using support vector machines (SVM) class prediction engine with leave-one-out cross-validation (BRB Array Tools). Sensitivity and selectivity of classification as well as positive predictive values (PPV) and negative predictive values (NPV) were assessed. Biological significance of the differences in gene expression pattern was analyzed using Gene Ontology and Biocarta^1^ databases. Gene lists were analyzed using GOHyperG^2^ and Bioconductor Package^3^. Three types of tests were used for estimation of signaling pathways statistical significance: least squares, Kolmogorov–Smirnoff, and Hotelling test. Statistical significance of real-time PCR results was estimated using non-parametric Kolmogorov–Smirnov test by SPSS 13 software (SPSS), with two-sided *p*-value threshold of *p* < 0.05.

### Data analysis workflow

In the majority of the analyses, we used Welch test for selection of genes with changed expression. When we compared more than two classes, we used one-way ANOVA, while for selection of genes in pairwise comparisons, we used *post hoc* Tukey test. For estimation of statistical significance of each gene, two types of selection criteria were applied: uncorrected *p*-value <0.001 and FDR <10%. Biological significance of gene lists obtained in consecutive comparisons was analyzed by searching for over-represented functional gene classes (according to Gene Ontology database) and signaling pathways (Biocarta repository). With the usage of linear discriminant analysis, we also checked whether selected gene lists may be used for classification of samples. Global test was used to confirm if a given gene list is statistically significant (25).

### Validation of the microarray results

First, we used qRT-PCR to compare expression level of 18 selected genes in the tissue samples that were used for microarray experiments. This set of samples was called a training set. Then, we analyzed expression level of selected genes in samples derived from an independent group of patients. This set of samples was called a test set. Disease-free survival (DFS) and overall survival (OS) were analyzed by the Kaplan–Meier method and compared between groups using the log-rank test. Differences in characteristics between groups of patients according to the quantitative real-time PCR estimated gene expression levels were evaluated by the χ^2^ test. A *p*-value of <0.05 was considered statistically significant. The analyses of survival time were performed using R Statistical Software.

## Results

We analyzed global gene expression pattern in ovarian cancer with respect to several defined clinicopathological and molecular features of the tumor. These were: histological tumor type and grade, FIGO (International Federation of Gynecologists and Obstetricians) clinical stage, the volume of residual tumor left after surgery, and a germline BRCA1 gene mutation. Among the clinical endpoints analyzed, there were response to the first line chemotherapy, DFS, and OS. Full lists of genes obtained in these comparisons, the results of hierarchical clustering, as well as the lists of over-represented gene ontology classes and signaling pathways characteristic for each trait are presented as supplementary Data Sheets.

### Histological type of the tumor

Epithelial ovarian cancers have heterogeneous histology; serous carcinomas are the most frequent ones while endometrioid, mucinous, clear cell, and undifferentiated tumors are relatively rare. All analyses performed in this study by alternative bioinformatic algorithms indicated that histological type of the tumor was the strongest factor affecting global gene expression pattern. When all four histological types were compared using one-way ANOVA, we found 3526 probe sets with significantly changed expression (FDR <10%; Data Sheet 2 in Supplementary Material). This difference was also significant in the global test (3651 probe sets, *p* < 0.001). None of the other features analyzed were associated with that large number of differentially expressed genes.

The annotated genes from the list obtained from ANOVA were taken for analysis of signaling pathways. Among significantly affected pathways were those engaged in cell cycle regulation, apoptosis, ubiquitination and sumoylation, signaling by estrogen receptor, GATA3, Trefoil factor, PTEN, and STAT (Data Sheet 3 in Supplementary Material).

We also performed pairwise comparisons (*post hoc* class comparison, Tukey test) to assess how many genes are differentially expressed between each two histological types of ovarian cancer (Table 2). Most pronounced molecular differences were observed between serous and clear cell tumors (625 differentially expressed probe sets with *p* < 0.001 and 40 probe sets with FDR <10%). Endometrioid and undifferentiated types were equally different from clear cell tumors. In the comparison of endometrioid and clear cell tumors, we observed 233 differentially expressed probe sets, *p* < 0.001 (12 probe sets with FDR <10%). Comparison of undifferentiated and clear cell tumors gave 237 probe sets, *p* < 0.001 (11 probe sets with FDR <10%).

**Table 2:** **Pairwise comparisons of different histological types of ovarian cancer (*post hoc* comparison, Tukey test)**.

	Endometrioid	Undifferentiated	Serous
Clear cell	233/12	237/11	625/40
Endometrioid	–	38/0	176/0
Undifferentiated		–	2/0

*Given in the table are the numbers of probe sets with significantly changed expression (no. of probe sets with *p* < 0.001/no. of probe sets with FDR <10%)*.

On the contrary, undifferentiated tumors were characterized by almost identical gene expression pattern to serous tumors (only two differentially expressed probe sets, *p* < 0.001; none of the probe sets with FDR <10%). Also in the global test, the difference between serous and undifferentiated tumors was insignificant (43 probe sets, *p* = 0.28). Taking into account this striking similarity, we decided to merge serous and undifferentiated ovarian cancer samples into one group and excluded clear cell and endometrioid tumors from the subsequent analyses in order to reduce unwanted sources of variability.

We also performed a linear discriminant analysis to check whether we can properly classify tumor samples according to the histological type, based on the expression level of selected genes (3526 probe sets selected in ANOVA were used for this purpose). Results of classification are given in Table 3. In total, we observed only 20% of incorrectly classified samples; the best classification rate was achieved for serous cancer (89%). Interestingly, all undifferentiated samples were wrongly classified as serous, again indicating that gene expression pattern of these two histological types is very similar.

**Table 3:** **Classification of the tumor samples according to the histological type using linear discriminant analysis**.

Histology	Sensitivity	Specificity	PPV	NPV	No misclassified/total no. (% misclassified)
Clear cell	0.778	1	1	0.978	2/9 (22)
Endometrioid	0.667	0.966	0.727	0.955	4/11 (36)
Serous	0.889	0.741	0.901	0.714	8/71 (11)
Undifferentiated	0	0.892	0	0.933	6/6 (100)
All					20

*PPV, positive predictive value; NPV, negative predictive value*.

### FIGO stage

Clinical cancer stage is one of the major prognostic factors. Ovarian cancer, which is the most deadly gynecological cancer, is usually diagnosed at an advanced stage. Our collection of samples was typical in this respect: the majority of patients were diagnosed at FIGO III stage. In order to analyze whether the advancement of the disease may be reflected by the changes in gene expression pattern, we compared 3 samples from patients diagnosed at stage II, 59 samples from stage III, and 10 samples from stage IV tumors (72 tumor samples in total).

When we used one-way ANOVA for comparison of three FIGO classes, we found 541 differentially expressed probe sets passing criterion of *p* < 0.001 (538 probe sets with FDR <10%). Among the most significant genes were *FOXE 1* (Forkhead box E1), *FLRT2* (Fibronectin leucine rich transmembrane protein 2), and *GRK6* (G protein-coupled receptor kinase 6).

However, in the global test the difference between FIGO stages appeared insignificant (25 probe sets, *p* = 0.75). Consequently, when we used the genes selected in ANOVA for classification of samples, the results were poor. Although 71% of samples were properly classified, the specificity was unacceptably low in respect to stage II and stage IV samples (Table 4).

**Table 4:** **Classification of tumor samples according to FIGO stage (linear discriminant analysis)**.

Stage	Sensitivity	Specificity	PPV	NPV	% Properly classified
FIGO II	0	1	–	0.958	
FIGO III	0.831	0.231	0.831	0.231	
FIGO IV	0.2	0.823	0.154	0.864	
All					71%

*PPV, positive predictive value; NPV, negative predictive*.

Also, in the subsequent pairwise comparisons (Tukey test) we found very low numbers of genes differentiating FIGO classes from each other. There were only one gene differentiating stage II from stage III and two genes showing changed expression between stage II and stage IV. These were *ATH1* (acid trehalase-like 1, yeast) and *AGR2* (anterior gradient homolog, *Xenopus laevis*) in stage II vs. IV comparison; the latter one was also significant for stage II vs. III difference.

For further analysis, we combined stage III and IV and compared this group of samples with stage II. This comparison yielded 714 probe sets, *p* < 0.001 (Data Sheet 4 in Supplementary Material) and 650 probe sets with FDR <10%. To better explore biological differences between early and advanced tumors, we performed analysis of gene ontology classes and signaling pathways that may be affected in these two groups (Data Sheet 5 in Supplementary Material). For this purpose, we used the annotated genes present on the list of 714 probe sets (*p* < 0.001), differentiating stage II from stage III/IV tumors. Among the most significantly over-represented gene ontology classes were those linked to the immunological processes, exogenous signal detection, neural transmission, and differentiation. Signaling pathways (according to Biocarta database), changed between early and advanced ovarian cancer, were those connected with immunological response and inflammation as well as cellular metabolism, apoptosis, PPAR, PKC, and TNFR signaling. These results, although interesting, must be taken with caution: possible bias could have been introduced due to uneven number of samples in the groups (3 stage II vs. 69 other samples).

### Grade

Histological tumor grade is the measure of cancer cells differentiation, with the high grade being a factor indicating bad prognosis. Among 77 analyzed tumor samples, 9 were defined as grade 2 (G2), 49 as G3, and 19 as G4. We were especially interested in defining the molecular difference between G3 and G4 as grade 4 is nowadays not commonly recognized, and most pathologists use the 3-grade scale.

In one-way ANOVA, we found 327 (*p* < 0.001) and 152 (FDR <10%) differentially expressed probe sets. In the global test, this difference appeared to be significant (257 probe sets, *p* < 0.001). However, in linear discriminant analysis only 55% of samples were properly classified; such result may likely be achieved by chance. Also, in pairwise comparisons (*post hoc* class comparison, Tukey test), we found only very limited numbers of differentially expressed genes: in G2 vs. G3 comparison – only one gene with *p* < 0.001 (10 probe sets with FDR <10%); for G2 vs. G4 and G3 vs. G4 comparisons no genes with *p* < 0.001 were obtained (5 and 1 probe set with FDR <10%, respectively).

These results indicate that although postulated tumor grade 4 may be distinguished histologically, it does not differ in gene expression pattern from grade 3 tumors. Thus, we merged G3 and G4 groups and compared them against G2, using Welch test, that yielded 411 (*p* < 0.001; Data Sheet 6 in Supplementary Material) and 267 (FDR <10%) probe sets, among them there were many uncharacterized or poorly characterized ones. Within this gene set, most over-represented gene ontology classes were associated with hemopoiesis, amino acid metabolism, and MAP kinase pathway (Data Sheet 7 in Supplementary Material). Among signaling pathways from Biocarta database, significantly engaged in this difference were: cdc25/chk1, pRB, src, sonic Hedgehog, G2/M checkpoint, and “role of BRCA1, BRCA2, and ATR in cancer susceptibility.”

### Cytoreduction

Usually, at the time of diagnosis, ovarian cancer spreads widely inside peritoneal cavity. The state of the art treatment of patients with this cancer is based on maximal possible surgical cytoreduction and adjuvant chemotherapy. The volume of residual tumor left after surgery is one of most important prognostic factors; the smaller is the size or volume of the residual tumor, the better for the patient. The best prognosis is reported for patients with no residual disease, while it is the worst for residual tumor above 5 cm in diameter. It has been already shown by Berchuck et al. that different sizes of residual tumor (<1 and >1 cm) are linked to different gene expression patterns (26). This might indicate that the size of residual tumor may not only be attributable to the successful removal of the tumor masses, but may be partially linked to the underlying biologic properties of the cancer.

Our analysis was done using the data from 72 cancer samples (serous and undifferentiated) for which the appropriate clinical data were available. In 15 cases, the residual tumor had diameter less than 1 cm (R0 group), 36 patients had tumor masses within 1–5 cm range (R1), while 21 cases had residual tumor over 5 cm in diameter (R2). Using one-way ANOVA, we found 349 probe sets with *p* < 0.001 and 63 probe sets with FDR <10%. Interestingly, in the global test, this difference was statistically significant (187 probe sets, *p* < 0.001). However, in *post hoc* Tukey test, only a few genes were found that differentiate the classes in pairwise comparisons. These were: one gene, *p* < 0.001 and seven genes, FDR <10% for R0/R1 difference, zero genes, *p* < 0.001 and two genes, FDR <10% for R1/R2 comparison and none for R0/R2. Thus, we merged groups R1 and R2 and compared it against R0 (a comparison alike that in the study by Berchuck et al.). Two-hundred and twelve probe sets with *p* < 0.001 (Data Sheet 8 in Supplementary Material) but only two with FDR <10% were found in Welch test. Only MAP3K7 gene was common in Berchuck’s and in our analysis. Gene ontology assessment revealed functional gene groups connected with embryo- and morphogenesis. The analysis according to Biocarta database showed signaling pathways related with chromatin remodeling as well as pathways regulated by CDK5, AKT, estrogen receptor, CDC25, CHK1, pRB, Fas, TNF, Ras, and NF-κB (Data Sheet 9 in Supplementary Material). The list of 349 probe sets (*p* < 0.001) obtained in Welch test was validated in linear discrimination analysis. Only 57% of the tumors were properly classified into classes R0, R1, and R2, the result likely obtained by chance.

### Response to chemotherapy

Ovarian cancer usually responds well to the first line chemotherapy and patients achieve either complete remission (CR) or partial remission (PR). Fewer numbers of tumors respond poorly, leading either to the stabilization of the disease [stable disease (SD)] or to progression (P). Among 72 tumor samples of serous or undifferentiated histology with sufficient clinical data, 62 were obtained from patients with either CR or PR, as it was established prospectively. These samples were classified as “chemotherapy-sensitive.” Another 10 samples were obtained from patients with SD or progression (P) and were classified as “chemotherapy-resistant.” Merging of SD and P samples seemed not only biologically valid, but was also justified by the low numbers of samples in these groups, the factor that can cause bias in the results of microarray data analysis. We found 196 differentially expressed probe sets, *p* < 0.001 (9 probe sets with FDR <10%) when comparing CR/PR vs. SD/P samples in Welch test (Data Sheet 10 in Supplementary Material). Majority of the top genes were uncharacterized, except for *SNX8* (sorting nexin 8) and *FGF12* (fibroblast growth factor 12). Gene ontology analysis (done on the annotated genes present at the list of 196 probe sets with *p* < 0.001) revealed only six functional classes significantly changed in this comparison, containing genes related with neuronal development, regulation of cell division, and WNT1 signaling (Data Sheet 11 in Supplementary Material). Analysis of signaling pathways (Biocarta repository) revealed only two affected pathways: “cyclins and cell cycle regulation” and the second one concerned with the neuronal signaling. To check whether the genes selected in Welch test may serve for classification of chemotherapy-sensitive vs. resistant tumors, we applied linear discrimination analysis. Although 81% of tumor samples were properly classified, the test showed unacceptably low specificity (10%) in respect to chemotherapy-sensitive samples and low sensitivity in detecting resistant tumors (10%). Thus, this test is without practical clinical value in respect to prediction of tumor response to chemotherapy.

### Prognostic factors

Among the genes that are differentially expressed in the tumors from patients with short and long survival times, putative prognostic molecular markers may be selected. Potentially, such markers could serve to predict patients’ prognosis and individually tailor the therapy in order to improve treatment outcome.

### Overall survival

The genes related to the OS were selected using Cox-regression model. Seventy-two tumor samples with sufficient clinical data were analyzed, all of serous or undifferentiated histology. We found 93 differentially expressed probe sets, *p* < 0.001, however, it must be noted that they were characterized by high FDR values (between 12 and 21%; Data Sheet 12 in Supplementary Material). The most significant were *ATRX* (α-thalassemia/mental retardation syndrome X-linked, RAD54 homolog) and *PI3KR1* (phosphoinositide-3-kinase, regulatory subunit 1). Odds ratio (OR) estimated for twofold increase in the expression level of those genes were: 6 for *ATRX* and 14.5 for *PI3KR1*. Several genes showed protective effect connected with its increased expression level (OR <1). They were, e.g., tyrosine phosphatases *PTPN2* and *PTPRS* (OR = 0.24 and 0.31, respectively), MRPS10 (OR = 0.22), *KCNC3* (potassium voltage-gated channel, Shaw-related subfamily, member 3; OR = 0.31), and *FBXW7* (F-box and WD-40 domain protein 7; OR = 0.32).

### Disease-free survival

The Cox-regression model was also applied to select the genes associated with DFS. We analyzed 72 tumor samples. Eighteen probe sets were selected with *p* < 0.001, however, FDR values were very poor (~85%; Data Sheet 13 in Supplementary Material). Two genes with the best *p*-values and highest OR rates calculated for twofold expression increase were: *CLASP1* (cytoplasmic linker associated protein 1) and *VAV2* oncogene (OD 3.5 and 3.7, respectively). *ATRX* was also present on this list (OR = 3.15). Among the genes with protective effect was *CDC42EP4* (CDC42 effector protein, Rho GTPase binding 4; OR = 0.4).

### Technical validation of the microarray results

**Figure 1:**
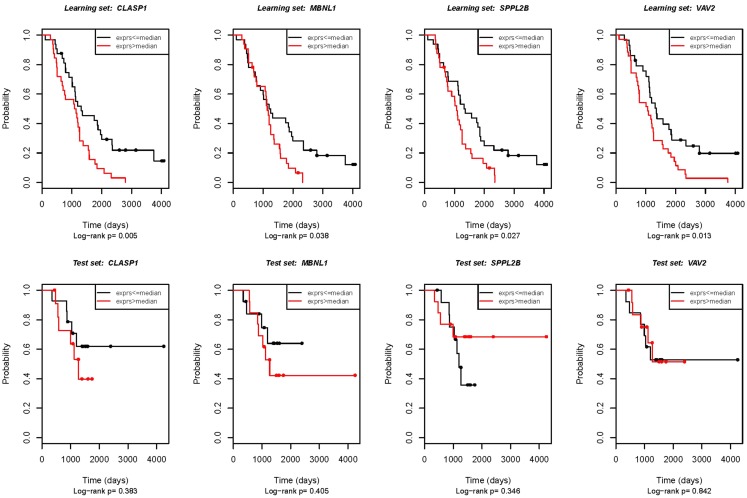
**Real-time RT-PCR validation of the genes potentially associated with OS**. First row: technical validation in the initial set of samples (the same samples that were used for the microarray experiment). Second row: external validation in the independent patient group. The Kaplan–Meier analysis plot of observed overall survival for patients with ovarian cancer by log-rank test according to real-time RT-PCR estimated gene expression.

To verify the microarray results, we analyzed expression of selected genes by quantitative RT-PCR. The same RNA samples were used for qRT-PCR as were previously analyzed in the microarray experiment. Fifteen genes related with OS were chosen for validation; two of those genes (*ATRX* and *CLASP1*) also showed an association with DFS. Four genes were confirmed to be significantly associated with OS (*p* < 0.05; see also Figure 1). These were *CLASP1* (*p* = 0.005), *MBNL1* [Muscle blind-like (*Drosophila*), *p* = 0.0381], *SPPL2B* (signal peptide peptidase-like 2B, *p* = 0.0271), and *VAV2* oncogene (*p* = 0.0133), however, correlation of expression of *ATRX* and *CLASP1* with DFS was not validated.

We also analyzed three genes associated with CHT-response, i.e., two cyclins: *CCNB1* and *CCNE1* and cyclin-dependent kinase inhibitor 2A (*CDKN2A*), however, none of them were positively validated by qRT-PCR.

Using the expression data obtained by real-time RT-PCR, we also performed few other comparisons to check, whether the genes previously selected as related to OS/DFS and CHT-response may be significantly correlated with other features (Table 5). We analyzed the so-called platinum-sensitivity (classified as follows: DFS <180 days means platinum-resistant tumor; DFS >180 means platinum-moderately sensitive one; DFS >732 days (2 years) means high platinum-sensitivity), as well as CHT-response (measured as CR and PR vs. SD and P). In addition, we analyzed an association of selected genes with hereditary BRCA1 mutation status. There are data indicating that tumors developing in patients with hereditary BRCA1 mutation respond better to DNA-damaging cytostatics than sporadic cancers, and thus BRCA1 testing may be important for therapeutic decisions [e.g., Ref. (27)].

**Table 5:** **Technical validation of microarray results by real-time RT-PCR**.

No.	Gene	Related to (in microarray analysis)	Statistical significance in real-time RT-PCR validation (*p*-value)				
			OS	DFS	CHT-response	Platinum-sensitivity	BRCA1 mutation
1	*AGGF*	OS	−		0.0818	**0.0293**	
2	*ATRX*	OS, DFS	−	−			
3	*CCNB1*	BRCA1, CHT-response	**0.0431**		−		
4	*CCNE1*	CHT-response	**0.0342**		−		
5	*CCNF*	OS	−				
6	*CDKN2A*	CHT-response			−		
7	***CLASP1***	OS, DFS	**0.0050**	−	**0.0005**		**0.0349**
8	*CTNND2*	OS	−				
9	*MRPS10*	OS	−	**0.0215**			
10	***MBNL1***	OS	**0.0381**	**0.0273**			
11	*PIK3R1*	OS	−				
12	*PRKCA*	OS, TP53 mutation	−				
13	*PSCD3*	OS	−		**0.0183**	**0.008**	
14	*PTPN2*	OS	−			**0.0248**	
15	***SPPL2B***	OS	**0.0271**	0.0684			
16	*STX7*	OS	−				
17	*USP1*	OS	−				
18	***VAV2***	OS, DFS	**0.0133**				

*The third column describes the feature that appeared to be significantly linked with a given gene in microarray analysis. Only statistically significant correlations measured at the validation step are shown. Minus in brackets, i.e., (−) indicates that the given gene was negatively validated in respect to the feature which it was related to in the microarray analysis. OS and DFS were analyzed by the Kaplan–Meier method (log-rank test). CHT-response and platinum-sensitivity were analyzed by Monte Carlo method (Kruskal–Wallis test). Correlations with the germline BRCA1 were calculated using Mann–Whitney *U* test. Statistically significant correlations are indicated in bold*.

Interestingly, *CLASP1*, in addition to its association with OS, showed also strong correlation with CHT-response (*p* = 0.0005) as well as with BRCA1 mutation status (*p* = 0.0349).

Expression of three genes: *AGGF* (angiogenic factor with G patch and FHA domains 1), *PSCD3* (pleckstrin homology 3), and *PTPN2* (protein tyrosine phosphatase, non-receptor type 2), which was not validated to correlate with OS, was proven to correlate with platinum-sensitivity. One of them (*PSCD3*) also showed correlation with CHT-response. Surprisingly, in this analysis, expression of both cyclins (*CCNB1* and *CCNE1*, not validated in respect to CHT-response) proved to be significantly correlated with OS.

### Validation in the independent group of patients

**Figure 2:**
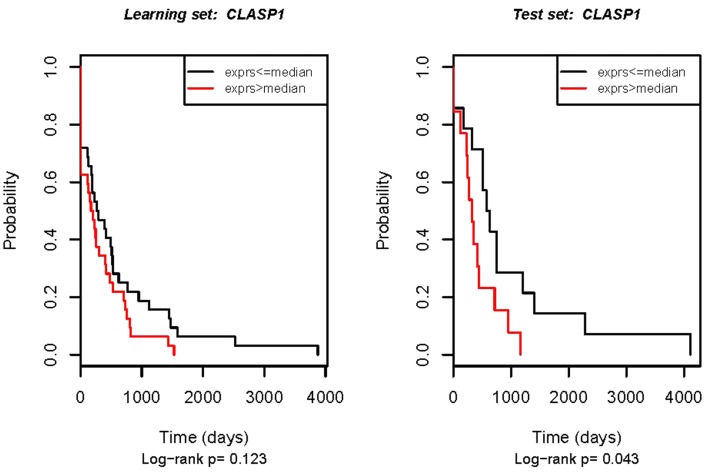
**Real-time RT-PCR validation of the *CLASP1* gene in relation to DFS**. Left: technical validation in the initial set of samples (the same samples that were used for the microarray experiment). Right: external validation in the independent patient group. The Kaplan–Meier analysis plot of observed DFS for patients with ovarian cancer by log-rank test according to real-time RT-PCR estimated gene expression.

The clinical importance of potential prognostic and predictive molecular markers must be reproducibly seen in different groups of patients, if the markers are to be used in practice. Thus, four genes that were validated in respect to OS (*MBNL1*, *SPPLB2*, *VAV2*, and *CLASP1*) were further tested in the independent set of 30 ovarian cancer samples. Disappointingly, none of these genes were validated according to OS in the independent set of samples. However, in this experiment, *CLASP1* turned out to be related to DFS again (Figure 2).

## Discussion

Expression microarrays are used to analyze molecular profiles of cancer in order to better understand the biological background of the disease. Another aim is to find new molecular markers, therapeutic targets, and/or new classification approaches that will enable better treatment of patients. Our study was intended to achieve both goals. We searched for gene expression patterns that may characterize histological types of ovarian cancer and are related to its histological grade, FIGO stage, response to chemotherapy, and survival times.

From the broad spectrum of features that we analyzed in our study, only histological type of the tumor was a factor, which showed a very strong impact on the gene expression pattern. Interestingly, there was one exception: six undifferentiated tumors that were available for this analysis, showed practically no difference in gene expression pattern from serous cancers. If confirmed in other studies, this may be an indication for evaluating these two groups together in microarray analyses.

On the contrary, the differences between serous/undifferentiated, endometrioid, and clear cell cancers were statistically highly significant. Moreover, the gene expression signature selected in respect to tumor histology allowed for a very precise sample classification, with the sensitivity and specificity not achieved in any other comparisons. Also unsupervised analysis, performed using the singular value decomposition (SVD) showed that histological type of the tumor is a major source of variability in the gene expression pattern in ovarian cancer (not shown). This large difference in gene expression pattern may be not surprising when we take into account that histological differences are clearly manifested at the morphological level and are easily distinguishable by light microscopy. On the other hand, these results, indicating deep molecular divergence, may support the current knowledge that ovarian cancer has a heterogeneous histological origin (e.g., fallopian, endometrioid, or endocervical) (28–32).

The histology of ovarian cancer was already analyzed in many previous microarray studies (33–40), however, it has not been regarded as a confounding factor in gene expression analysis in respect to other features. Conversely, different factors have been analyzed across various histological types. This may be one of the reasons for discrepancies and low reproducibility of the findings. Thus, a practical conclusion may be drawn that when searching for the genes related to other features of ovarian cancer, the analyses should be carried out on histologically homogenous groups of samples. Alternatively, the influence of the histological type on gene expression may be controlled by multivariate approach.

Except for evaluation of histological type, no other comparison gave such a huge number of statistically significant genes. This was the reason why we decided to use less stringent criteria for gene selection (uncorrected *p*-value <0.001 and FDR <10%). Analyzing gene expression patterns in tumor samples of different grades, we focused mostly on the difference between grade 3 and 4, as the usage of the latter grade was abandoned in ovarian cancer diagnostics. A study performed by members of our group showed that the recognition of grade 4 might be important from the clinical viewpoint, since patients with grade 4 ovarian cancer had worse response to taxanes than to DNA-damaging agents (23). Thus, we expected that we would find differences between grade 3 and 4 also at the molecular level. However, samples classification was poor and in pairwise comparison we found only one gene with significantly changed expression (FDR <10%). It was surprising, as in ANOVA we found 152 probe sets (FDR <10%) differentiating between three grades (G2, G3, and G4), and this difference was also significant in the global test. In our opinion, this discrepancy may suggest that although tumor grade is generally associated with significant changes in gene expression pattern, the subjectively defined grades 3 and 4 may not reflect these differences. An additional factor influencing the results of this analysis may be the small and unequal size of the groups evaluated.

The problem also occurred when analyzing gene expression profiles in relation to FIGO stages and residual tumor size. These features were significant in the ANOVA and global tests, but the number of genes with different expression found in pairwise comparisons was low and the quality of classification was poor. The difference between FIGO II and FIGO III/IV was statistically significant, however, this result may be an artifact related to uneven samples distribution in the groups being compared.

As far as the residual tumor size is concerned, poor classification of tumor samples may be due to the fact that debulking status did not solely depend on the biological tumor profile, but also on the changing attitude to optimal debulking over several years during which our material was collected. Other factors influencing the results might be technical issues, such as skills of surgeons and the equipment available. Our samples came from mid 1990s (patients treated with platinum–cyclophosphamide, PC), and from early 2000s (patients treated with taxane–platinum, TP). The group treated with PC had been generally less radically operated than the group treated with TP (23). Thus, this may be the major reason why it was hard to obtain reliable results in gene expression analysis in respect to this parameter. In addition, the arbitrarily outlined classes (R0–2) may not reflect intrinsic biological differences.

The most important, from the clinical point of view, is the search for molecular markers suitable for prediction of tumor response to the therapy. In the presented analysis, we were not able to find a gene signature that would allow for good classification of samples sensitive and resistant to chemotherapy. It seems that chemosensitivity/resistance, in contrast to, e.g., histological type, is a feature that may depend on subtle molecular changes, possibly in many alternative pathways. Such differences may be hard to detect by the methods applied. It has been shown recently, by comparing the data from Cancer Cell Line Encyclopedia and Cancer Genome Project, that discrepancies in drug sensitivity testing are common even when performed on cell lines (41). Another reason for the failure of this analysis may be again the fact that we analyzed two cohorts of patients treated with different CHT regimens. Probably, different molecular pathways were engaged in tumor response to the two regimens and this could affect the results of our analyses. It was not advisable, however, to divide patients into two groups according to the CHT regimen, because this would result in biased results due to small classes of samples.

We also searched for genes that may be related to patients’ prognosis, i.e., DFS and OS. Only 4 out of 15 genes, selected in microarray analysis as associated with OS, were positively validated by qRT-PCR, and none were validated for DFS. Our further attempts to validate these four genes in the independent set of samples were unsuccessful. There may be several reasons for this result. First, all genes selected in respect to survival time were of low statistical significance in the microarray analysis. Second, contrarily to the initial group, the independent set of patients used for validation was uniformly treated with TP regimen only. Therefore, it might show results different from those obtained in the initial, mixed group. Indeed, we observed that the initial group of patients had different OS statistics than the test group (Table 6).

**Table 6:** **Characteristics of the two groups of patients according to OS statistics (days)**.

Group	Minimal OS	First quartile	Median OS	Third quartile	Mean OS	Max. OS
Learning set	104	687	1131	1306	1773	4080
Test set	346	885.5	1199.0	1267.0	1468.0	4250

*Learning set, ovarian cancer samples used for the microarray analysis; test set, ovarian cancer samples from the independent group of patients, used for external validation*.

In general, the results of qRT-PCR validation were surprising. Several genes that were selected as related to one feature appeared to correlate with another factor(s). In our opinion, this observation confirms that many clinical and biological features of the tumor are difficult to define and that arbitrarily assigned groups of samples used in gene expression analyses not always reflect biologically significant differences.

Our attempts to validate selected genes were rather unsuccessful. It should be noted, however, that we performed an external validation on the independent group of tumor samples, while many other studies that claim finding potential biomarkers, were confined just to the internal, technical validation [reviewed, e.g., in Ref. (2, 42)].

One of the most interesting genes selected in our study is *CLASP1* (cytoplasmic linker associated protein 1). It was associated with both OS and DFS in the microarray analysis, although validation results were mixed. In the initial group of samples, it was validated in respect to OS and showed significant association with response to chemotherapy and with the presence of hereditary BRCA1 mutation. Surprisingly, when we tried to validate *CLASP1* in the independent set of samples it was statistically insignificant in respect to OS, but it proved to be associated again with DFS. *CLASP1* is thought to play a role in the regulation of microtubule dynamics in interphase and during cell division (43, 44). Thus, the protein may be important in tumor cell response to taxanes. Possibly, it may also be somehow engaged in differential response to CHT in patients with hereditary, BRCA1 mutation-linked ovarian cancer. Regardless of the inconsistent results of validation, we think that *CLASP1* may be worth further investigation as a potential prognostic and predictive marker.

## Conclusion

Our results confirm previous observations that histological type of the tumor is the major source of variability in gene expression in ovarian cancer. This statement does not refer, however, to the difference between serous and undifferentiated tumors. In our analyses, these two histological types showed almost identical gene expression pattern and were evaluated as one group. Taking into account large differences in molecular profile between serous/undifferentiated vs. endometrioid vs. clear cell tumors, we think that it is advisable to perform analyses of other clinical and molecular features of ovarian cancer only on the histologically homogenous groups of samples. In our opinion, the mixed results of quantitative RT-PCR validation shed light on the general problem that is present in supervised analyses of microarray results. In such approach, one arbitrarily defines the groups of tumors to be compared in terms of gene expression pattern. Most likely, arbitrarily performed division of samples may not reflect biological diversity of the tumors. In our opinion, this may be one of the reasons why, the results of such studies are often inconclusive and hard to replicate in different experimental settings.

## Author Contributions

Katarzyna Marta Lisowska took part in designing the study and preparing grant application; did some microarray experiments; analyzed the results; and drafted the manuscript, Magdalena Olbryt did the majority of microarray experiments, Volha Dudaladava and Jolanta Pamuła-Piłat did some microarray experiments, Ewa Grzybowska took part in designing the study and in preparing grant application, Katarzyna Kujawa did qRT-PCR experiments, Michał Jarzab did majority of bioinformatic analyses, Sebastian Student did some bioinformatic analyses, Iwona Krystyna Rzepecka did genetic tests, Barbara Jarzab took part in designing the study and helped to analyze the results, Jolanta Kupryjanczyk collected all tumor samples, did pathological assessments, and provided all clinical and molecular data; she also took part in designing the study; preparing the grant application; and writing the final version of the manuscript.

## Conflict of Interest Statement

The authors declare that the research was conducted in the absence of any commercial or financial relationships that could be construed as a potential conflict of interest.

## Supplementary Material

The Supplementary Material for this article can be found online at http://www.frontiersin.org/journal/10.3389/fonc.2014.00006/abstract

## Footnotes

^1^www.biocarta.com

^2^www.geneontology.org

^3^www.bioconductor.org
