# Supplementary material for: Gene Expression Analysis in Ovarian Cancer – Faults and Hints from DNA Microarray Study
Source: Front Oncol. 2014 Jan 28;4:6. doi: 10.3389/fonc.2014.00006 (PMC3904181; doi:10.3389/fonc.2014.00006)
Supplement: Supplementary file 1 [file 61993_Lisowska_DataSheet1.PDF]

Supplementary Table 1. The sequence of the primers and the probe numbers used for real-time RT-PCR

Gene	Probe no (Roche)	Forward primers	Reverse primers
**Experimental genes**			
AGGF1	#36	F 5’ tggaccattttgcctcaaat	R 5’ aggtatcttctgttgctaatgcag
ATRX	#06	F 5’ tggtacttctctttgaaattcttcg	R 5’ agggactggctgaaaacaag
CCNB1	#18	F 5’ catggtgcactttcctcctt	R 5’ aggtaatgttgtagagttggtgtcc
CCNE1	#36	F 5’ ggccaaaatcgacaggac	R 5’ gggtctgcacagactgcat
CCNF	#03	F 5’ cgaaaactcagggactacgc	R 5’ tttgcacaggctttggcta
CDKN2A	#34	F 5’ gtggacctggctgaggag	R 5’ ctttcaatcggggatgtctg
CLASP1	#63	F 5’ gctgttgctgatgctttgaa	R 5’ gaatacatcccatacggctca
CTNND2	#64	F 5’ cccagaccaaactgctcatc	R 5’ ctgctttgggattcaggttc
MBNL1	#47	F 5’ ggttgggtttgttggtttca	R 5’ gtccccctctggaactctct
MRPS10	#09	F 5’ aatgcttgtgtatcagacttacgttac	R 5’ gcacaaggctgcaattcttt
PIK3R1	#16	F 5’ aatgaacgacagcctgcac	R 5’ ccgttgttggctacagtagtagg
PRKCA	#75	F 5’ tggcgtcctgttgtatgaaa	R 5’ tcgtcttcatcttcaccatca
PSCD3	#33	F 5’ gacccacaccttcttcaacc	R 5’ gcttccaggtcttcacacg
PTPN2	#49	F 5’ acaatggaggagaacagtgagag	R 5’ ttcatctgctgcaccttctg
SPPL2B	#66	F 5’ tcctgggtttcggagacat	R 5’ caaacctgtggcagtaggc
TP53	#12	F 5’ aggccttggaactcaaggat	R 5’ cccttttggacttcaggtg
STX7	#84	F 5’ agcaaatcagcagctgtcaa	R 5’ tgatgcacagggtttttctg
VAV2	#39	F 5’ gggacgacatctacgaggac	R 5’ ttcagtcatgcccattttca
USP1	#77	F 5’ gtgctttgctgctagtggttt	R 5’ gaggtgtcaataaaggagtgttga
**Reference genes**			
ATP6V1	#03	F 5’ aagccggctggatctcat	R 5’ gcatttgcaccaaacaagg
HADHA	#65	F 5’ gtcttgcgcccatgatgt	R 5’ cagcttcttcgggtcaactc
UBE2D2	#67	F 5’ aatggcagcatttgtcttga	R 5’ cacaacagagaacagatgga
