# Supplementary material for: Gene Expression Analysis in Ovarian Cancer – Faults and Hints from DNA Microarray Study
Source: Front Oncol. 2014 Jan 28;4:6. doi: 10.3389/fonc.2014.00006 (PMC3904181; doi:10.3389/fonc.2014.00006)
Supplement: Supplementary file 2 [file 61993_Lisowska_DataSheet2.PDF]

Supplementary Table 2. Histological type of ovarian cancer

List of genes with significantly changed expression in different histological types of ovarian cancer (serous, undifferentiated, clear cell and endometrioid). 3526 probe sets with FDR<10% were selected using one-way ANOVA. Only top 500 genes are shown here.

**Affymetrix probe set ID**	**FDR**	**Gene symbol**	**Gene name**
229021_at	7,850E-10	MCTP2	Multiple C2-domains with two transmembrane regions 2
200953_s_at	8,430E-10	CCND2	cyclin D2
244784_at	1,950E-08	DHX57	DEAH (Asp-Glu-Ala-Asp/His) box polypeptide 57
1554235_at	1,950E-08	CTNNA3	catenin (cadherin-associated protein), alpha 3
236118_at	2,730E-08	LOC201484	hypothetical LOC201484
1562633_at	4,170E-08	NCRMS	non-coding RNA in rhabdomyosarcoma (RMS)
1557639_at	5,170E-08		CDNA FLJ35450 fis, clone SMINT2004280
1554060_s_at	5,170E-08	SETMAR	SET domain and mariner transposase fusion gene
224251_at	5,510E-08	C10orf79	chromosome 10 open reading frame 79
235209_at	7,710E-08	RPESP	RPE-spondin
201478_s_at	9,210E-08	DKC1	dyskeratosis congenita 1, dyskerin
244065_at	9,850E-08	CNTNAP3B	contactin associated protein-like 3B
223245_at	1,150E-07	STRBP	spermatid perinuclear RNA binding protein
244422_at	1,150E-07		Transcribed locus
220692_at	1,150E-07	HSPC047	HSPC047 protein
201479_at	1,150E-07	DKC1	dyskeratosis congenita 1, dyskerin
219005_at	1,650E-07	C19orf4	chromosome 19 open reading frame 4
237024_at	2,000E-07	FLJ38608	hypothetical protein FLJ38608
237097_at	2,180E-07	SPTBN5	Spectrin, beta, non-erythrocytic 5
228719_at	2,180E-07	TTC19	Hypothetical protein LOC125150
242600_at	2,220E-07	FRMD3	FERM domain containing 3
230165_at	2,220E-07	SGOL2	shugoshin-like 2 (S, pombe)
236150_at	2,300E-07	LOC123688	similar to RIKEN cDNA C630028N24 gene
201675_at	2,530E-07	AKAP1	A kinase (PRKA) anchor protein 1
213918_s_at	2,810E-07	NIPBL	Nipped-B homolog (Drosophila)
244134_at	2,820E-07	OXCT1	3-oxoacid CoA transferase 1
238836_at	3,410E-07		
206798_x_at	3,410E-07	DLEC1	deleted in lung and esophageal cancer 1
216881_x_at	5,540E-07	PRB4	proline-rich protein BstNI subfamily 4
217119_s_at	7,260E-07	CXCR3	chemokine (C-X-C motif) receptor 3
215062_at	7,260E-07	FMNL2	Formin-like 2
217379_at	7,260E-07		
1560661_x_at	7,260E-07	LOC150759	KIAA1641
235210_s_at	7,300E-07	RPESP	RPE-spondin
233014_at	7,630E-07	WDR37	WD repeat domain 37
227278_at	7,630E-07		Transcribed locus, moderately similar to XP_512541,1 PREDICTED: similar to hypothetical protein [Pan troglodytes]
227127_at	7,630E-07	MGC52022	Musculoskeletal, embryonic nuclear protein 1
224090_s_at	7,890E-07	TNFRSF19	tumor necrosis factor receptor superfamily, member 19
1553367_a_at	8,760E-07	COX6B2	cytochrome c oxidase subunit VIb polypeptide 2 (testis)
228842_at	8,930E-07		CDNA: FLJ22063 fis, clone HEP10326
206055_s_at	8,930E-07	SNRPA1	small nuclear ribonucleoprotein polypeptide A'
235545_at	9,680E-07	DEPDC1	DEP domain containing 1
205617_at	1,070E-06	PRRG2	proline rich Gla (G-carboxyglutamic acid) 2
211304_x_at	1,130E-06	KCNJ5	potassium inwardly-rectifying channel, subfamily J, member 5
204889_s_at	1,130E-06	NEURL	neuralized-like (Drosophila)
212900_at	1,240E-06	SEC24A	SEC24 related gene family, member A (S, cerevisiae)
1560659_at	1,290E-06	LOC150759	KIAA1641
227021_at	1,290E-06	AOF1	amine oxidase (flavin containing) domain 1

215606_s_at	1,430E-06	RAB6IP2	RAB6 interacting protein 2
1558195_at	1,460E-06	LOC283404	hypothetical protein LOC283404
222402_at	1,460E-06	C13orf12	chromosome 13 open reading frame 12
201926_s_at	1,460E-06	DAF	decay accelerating factor for complement (CD55, Cromer blood group system)
201813_s_at	1,460E-06	TBC1D5	TBC1 domain family, member 5
236042_at	1,460E-06		CDNA clone IMAGE:4752264
221959_at	1,460E-06	C8orf72	chromosome 8 open reading frame 72
207047_s_at	1,460E-06	CLCNKA ; CLCNKB	chloride channel Ka ; chloride channel Kb
1553971_a_at	1,500E-06	GATS	opposite strand transcription unit to STAG3
232858_at	1,500E-06	SCMH1	Sex comb on midleg homolog 1 (Drosophila)
211893_x_at	1,530E-06	CD6	CD6 antigen
215838_at	1,600E-06	LILRA5	leukocyte immunoglobulin-like receptor, subfamily A (with TM domain), member 5
204875_s_at	1,600E-06	GMDS	GDP-mannose 4,6-dehydratase
231355_at	1,610E-06		CDNA FLJ16240 fis, clone HCASM2003357
243016_at	1,610E-06	TYMS	Thymidylate synthetase
1564093_at	1,720E-06	NEK1	NIMA (never in mitosis gene a)-related kinase 1
227531_at	1,840E-06		CDNA FLJ39000 fis, clone NT2RI2022468
202189_x_at	1,870E-06	PTBP1	polypyrimidine tract binding protein 1
214888_at	1,870E-06	CAPN2	calpain 2, (m/II) large subunit
241361_at	1,870E-06	KIAA1833	Hypothetical protein KIAA1833
220459_at	1,870E-06	MCM3APAS	MCM3 minichromosome maintenance deficient 3 (S, cerevisiae) associated protein antisense
1562009_a_at	1,970E-06		CDNA FLJ40764 fis, clone TRACH2002954
228143_at	1,970E-06	CP	ceruloplasmin (ferroxidase)
208623_s_at	1,970E-06	VIL2	villin 2 (ezrin)
240385_at	1,970E-06	GATA6	GATA binding protein 6
211946_s_at	1,970E-06	BAT2D1	BAT2 domain containing 1
219359_at	2,520E-06	FLJ22635	hypothetical protein FLJ22635
205262_at	2,520E-06	KCNH2	potassium voltage-gated channel, subfamily H (eag-related), member 2
238356_at	2,730E-06	DOCK11	dedicator of cytokinesis 11
241881_at	2,760E-06	OR2W3	olfactory receptor, family 2, subfamily W, member 3
216589_at	2,800E-06	LOC390998	similar to 60S ribosomal protein L10 (QM protein) (Tumor suppressor QM) (Laminin receptor homolog)
39549_at	2,820E-06	NPAS2	neuronal PAS domain protein 2
218806_s_at	2,820E-06	VAV3	vav 3 oncogene
1558340_at	2,820E-06	DIXDC1	DIX domain containing 1
240053_x_at	2,820E-06		Full length insert cDNA clone YY86C01
212197_x_at	2,850E-06	M-RIP	myosin phosphatase-Rho interacting protein
230525_at	2,890E-06	LOC388206	hypothetical LOC388206
216977_x_at	3,100E-06	SNRPA1	small nuclear ribonucleoprotein polypeptide A'
240727_s_at	3,220E-06	RPESP	RPE-spondin
241206_at	3,250E-06	A2M	Alpha-2-macroglobulin
204565_at	3,530E-06	THEM2	thioesterase superfamily member 2
241508_at	3,530E-06	ANKRD12	Ankyrin repeat domain 12
1560372_at	4,080E-06		CDNA FLJ34680 fis, clone LIVER2003524
1561795_at	4,080E-06		Homo sapiens, clone IMAGE:4276820, mRNA
229292_at	4,480E-06	EPB41L5	erythrocyte membrane protein band 4,1 like 5
1556309_s_at	4,480E-06	FLJ31031	Chromosome 1 open reading frame 86
203000_at	4,520E-06	STMN2	stathmin-like 2
1554953_a_at	4,540E-06	C21orf90	chromosome 21 open reading frame 90
202589_at	4,540E-06	TYMS	thymidylate synthetase
242321_at	4,540E-06		CDNA FLJ32401 fis, clone SKMUS2000339
237930_at	4,840E-06		Transcribed locus
240550_at	4,890E-06		Transcribed locus

218057_x_at	4,940E-06	COX4NB	COX4 neighbor
235283_at	4,960E-06	DDX26	DEAD/H (Asp-Glu-Ala-Asp/His) box polypeptide 26
223407_at	5,250E-06	C16orf48	chromosome 16 open reading frame 48
215722_s_at	5,400E-06	SNRPA1	small nuclear ribonucleoprotein polypeptide A'
231359_at	5,400E-06	APOH	Apolipoprotein H (beta-2-glycoprotein I)
232541_at	5,400E-06	EGFR	Epidermal growth factor receptor (erythroblastic leukemia viral (v-erb-b) oncogene homolog, avian)
204743_at	5,710E-06	TAGLN3	transgelin 3
226255_at	5,710E-06	ZBTB33	zinc finger and BTB domain containing 33
242097_at	5,710E-06	NETO2	Neuropilin (NRP) and tolloid (TLL)-like 2
226782_at	5,750E-06	SLC25A30	solute carrier family 25, member 30
241954_at	5,830E-06	FDFT1	Farnesyl-diphosphate farnesyltransferase 1
213703_at	5,930E-06	LOC150759	hypothetical protein LOC150759
226779_at	6,080E-06	DKFZp434H2226	LMBR1 domain containing 2
238191_at	6,110E-06	chromosome 1 open reading frame 192	Similar to RIKEN cDNA 1700009P17
219542_at	6,110E-06	NEK11	NIMA (never in mitosis gene a)- related kinase 11
206830_at	6,210E-06	SLC4A10	solute carrier family 4, sodium bicarbonate transporter-like, member 10
1570339_x_at	6,380E-06	MGC25181	Hypothetical protein MGC25181 ; CDNA FLJ26792 fis, clone PRS04865
1556173_a_at	6,810E-06		CDNA FLJ43972 fis, clone TESTI4017961
205034_at	6,860E-06	CCNE2	cyclin E2
1565034_s_at	6,930E-06	AFF3 ; MLL	AF4/FMR2 family, member 3 ; myeloid/lymphoid or mixed-lineage leukemia (trithorax homolog, Drosophila)
224031_at	7,050E-06	STK17A	Serine/threonine kinase 17a (apoptosis-inducing)
223986_x_at	7,690E-06	DMRT2	doublesex and mab-3 related transcription factor 2
215540_at	7,950E-06	TRA@	T cell receptor alpha locus
238185_at	7,950E-06	RBMS1	RNA binding motif, single stranded interacting protein 1
235407_at	7,950E-06		
206319_s_at	7,950E-06	SPINLW1	serine peptidase inhibitor-like, with Kunitz and WAP domains 1 (eppin)
230981_at	8,000E-06	CATSPER3	cation channel, sperm associated 3
223868_s_at	8,060E-06	WWOX	WW domain containing oxidoreductase
206315_at	8,110E-06	CRLF1	cytokine receptor-like factor 1
230786_at	8,110E-06		Transcribed locus, weakly similar to XP_518535,1 PREDICTED: similar to dJ108C2,1,4 (MCM3 minichromosome maintenance deficient 3 (S, cerevisiae), variant 4) [Pan troglodytes]
1554951_at	8,110E-06	LOC554247	hypothetical LOC554247
227297_at	8,110E-06	ITGA9	Integrin, alpha 9
1553946_at	8,110E-06	DCD	dermcidin
243256_at	8,250E-06	MKNK1	MAP kinase interacting serine/threonine kinase 1
236161_at	8,250E-06		Transcribed locus
220764_at	8,250E-06	PPP4R2	protein phosphatase 4, regulatory subunit 2
1569746_s_at	8,330E-06		CDNA clone IMAGE:30343195
222723_at	8,720E-06	VWA1	von Willebrand factor A domain containing 1
214434_at	8,720E-06	HSPA12A	heat shock 70kDa protein 12A
1555180_at	8,910E-06	C11orf34	Hypothetical LOC399951
201326_at	8,910E-06	CCT6A	chaperonin containing TCP1, subunit 6A (zeta 1)
223512_at	8,910E-06	SARA2	SAR1a gene homolog 2 (S, cerevisiae)
212289_at	8,940E-06	ANKRD12	ankyrin repeat domain 12
201661_s_at	9,260E-06	ACSL3	acyl-CoA synthetase long-chain family member 3
206485_at	9,260E-06	CD5	CD5 antigen (p56-62)
206270_at	9,310E-06	PRKCG	protein kinase C, gamma
1561161_at	9,370E-06	SCMH1	Sex comb on midleg homolog 1 (Drosophila)
206047_at	9,580E-06	GNB3	guanine nucleotide binding protein (G protein), beta polypeptide 3

1559601_at	1,060E-05	KIAA2018	KIAA2018
201372_s_at	1,080E-05	CUL3	cullin 3
1555898_at	1,080E-05	LOC150759	Hypothetical protein LOC150759
233963_at	1,120E-05	LOC392512	similar to histone H2B-related protein
1559714_at	1,140E-05	RGR	retinal G protein coupled receptor
226181_at	1,140E-05	TUBE1	tubulin, epsilon 1
211428_at	1,140E-05	SERPINA1	serpin peptidase inhibitor, clade A (alpha-1 antiproteinase, antitrypsin), member 1
204358_s_at	1,170E-05	FLRT2	fibronectin leucine rich transmembrane protein 2
234622_at	1,200E-05	LOC221814	Similar to RIKEN cDNA 4930579E17
239491_at	1,240E-05	LASS5	LAG1 longevity assurance homolog 5 (S, cerevisiae)
200725_x_at	1,260E-05	RPL10	ribosomal protein L10
220759_at	1,280E-05	FAM12B	family with sequence similarity 12, member B (epididymal)
209881_s_at	1,300E-05	LAT	linker for activation of T cells
224959_at	1,300E-05	SLC26A2	solute carrier family 26 (sulfate transporter), member 2
221092_at	1,310E-05	ZNFN1A3	zinc finger protein, subfamily 1A, 3 (Aiolos)
234548_at	1,340E-05	HSPC117	Hypothetical protein HSPC117
1554493_s_at	1,400E-05	THADA	thyroid adenoma associated
202610_s_at	1,400E-05	CRSP2	cofactor required for Sp1 transcriptional activation, subunit 2, 150kDa
1561642_at	1,400E-05	PHACTR1	Phosphatase and actin regulator 1
226531_at	1,400E-05	FLJ14466	hypothetical protein FLJ14466
240616_at	1,440E-05		Transcribed locus, weakly similar to XP_496299,1 PREDICTED: hypothetical protein LOC148206 [Homo sapiens]
1564705_at	1,440E-05	GLS2	glutaminase 2 (liver, mitochondrial)
229915_at	1,440E-05	LOC441168	hypothetical protein LOC441168
210324_at	1,470E-05	C8G	complement component 8, gamma polypeptide
222939_s_at	1,510E-05	SLC16A10	solute carrier family 16 (monocarboxylic acid transporters), member 10
228281_at	1,510E-05	FLJ25416	hypothetical protein FLJ25416
231620_at	1,510E-05		
213735_s_at	1,510E-05	COX5B	cytochrome c oxidase subunit Vb
214565_s_at	1,510E-05	SMR3B ; SMR3A	submaxillary gland androgen regulated protein 3 homolog B (mouse) ; submaxillary gland androgen regulated protein 3 homolog A (mouse)
223669_at	1,530E-05	HEMGN	hemogen
1560237_at	1,570E-05		CDNA FLJ14487 fis, clone MAMMA1002721
1561481_at	1,620E-05		CDNA clone IMAGE:4827393
242126_at	1,630E-05	EXT1	Exostoses (multiple) 1
236656_s_at	1,640E-05		Full length insert cDNA YI37C01
1554528_at	1,650E-05	C3orf15	chromosome 3 open reading frame 15
201443_s_at	1,650E-05	ATP6AP2	ATPase, H+ transporting, lysosomal accessory protein 2
226752_at	1,770E-05	UNQ1912	HGS_RE408
1566528_at	1,860E-05	CUTL2	Cut-like 2 (Drosophila)
1560866_at	1,860E-05	WNK2	WNK lysine deficient protein kinase 2
226755_at	1,870E-05		Nasopharyngeal carcinoma-associated antigen NPC-A-5
1569938_at	1,870E-05	SIRT5	sirtuin (silent mating type information regulation 2 homolog) 5 (S, cerevisiae)
208616_s_at	1,930E-05	PTP4A2	protein tyrosine phosphatase type IVA, member 2
221842_s_at	1,950E-05	ZNF131	zinc finger protein 131 (clone pHZ-10)
221785_at	2,000E-05	WIZ	widely-interspaced zinc finger motifs
206259_at	2,020E-05	PROC	protein C (inactivator of coagulation factors Va and VIIIa)
214401_at	2,020E-05	PAX1	paired box gene 1
214156_at	2,020E-05	MYRIP	myosin VIIA and Rab interacting protein
214748_at	2,020E-05	LOC88523	CG016
243677_at	2,020E-05	STI2	Tetratricopeptide repeat domain 21A
235527_at	2,020E-05	LOC284214	hypothetical protein LOC284214
201501_s_at	2,020E-05	GRSF1	G-rich RNA sequence binding factor 1

236832_at	2,040E-05	LOC221442	hypothetical protein LOC221442
1555764_s_at	2,060E-05	TIMM10	translocase of inner mitochondrial membrane 10 homolog (yeast)
210913_at	2,060E-05	CDH20	cadherin 20, type 2
218850_s_at	2,090E-05	LIMD1	LIM domains containing 1
208602_x_at	2,120E-05	CD6	CD6 antigen
205777_at	2,140E-05	DUSP9	dual specificity phosphatase 9
207104_x_at	2,150E-05	LILRB1	leukocyte immunoglobulin-like receptor, subfamily B (with TM and ITIM domains), member 1
210806_at	2,160E-05	TTLL5	tubulin tyrosine ligase-like family, member 5
206665_s_at	2,160E-05	BCL2L1	BCL2-like 1
237770_at	2,180E-05		Transcribed locus, moderately similar to XP_497042,1 PREDICTED: hypothetical protein XP_497042 [Homo sapiens]
1568780_at	2,230E-05		Hypothetical LOC497257
210475_at	2,230E-05	POU3F1	POU domain, class 3, transcription factor 1
239211_at	2,230E-05	ANKRD11	Ankyrin repeat domain 11
226044_at	2,230E-05	TDP1	tyrosyl-DNA phosphodiesterase 1
226212_s_at	2,250E-05	INSR	Insulin receptor
225304_s_at	2,250E-05	NDUFA11	NADH dehydrogenase (ubiquinone) 1 alpha subcomplex, 11, 14,7kDa
221251_x_at	2,260E-05	ZNHIT4	zinc finger, HIT type 4 ; zinc finger, HIT type 4
222721_at	2,510E-05	DOCK5	Dedicator of cytokinesis 5
243234_at	2,510E-05	TBX3	T-box 3 (ulnar mammary syndrome)
1553151_at	2,620E-05	ATP6V0D2	ATPase, H+ transporting, lysosomal 38kDa, V0 subunit d isoform 2
218628_at	2,630E-05	CGI-116	CGI-116 protein
1561671_at	2,660E-05	LOC286121	hypothetical protein LOC286121
1560495_at	2,700E-05		CDNA clone IMAGE:5729277
210983_s_at	2,710E-05	MCM7	MCM7 minichromosome maintenance deficient 7 (S, cerevisiae)
232639_at	2,760E-05	LOC90288	hypothetical protein LOC90288
216311_at	2,760E-05	LOC400688	similar to comment for location 3447-3655: BLASTX gi|103290|pir||S16356 ovo protein - fruit fly (Drosophila melanogaster), PVal= 3,8e-47
218320_s_at	2,770E-05	NDUFB11	NADH dehydrogenase (ubiquinone) 1 beta subcomplex, 11, 17,3kDa
204967_at	2,780E-05	APXL	apical protein-like (Xenopus laevis)
216748_at	2,820E-05	PYHIN1	pyrin and HIN domain family, member 1
1558613_at	2,840E-05	NS5ATP13TP2	NS5ATP13TP2 protein
222557_at	2,900E-05	STMN3	stathmin-like 3
228108_at	2,980E-05		CDNA FLJ30761 fis, clone FEBRA2000538
229640_x_at	2,980E-05		Similar to hypothetical protein, MGC:7199
1569023_a_at	2,990E-05		Homo sapiens, Similar to otoconin 90, clone IMAGE:4279181, mRNA
211800_s_at	3,090E-05	USP4	ubiquitin specific peptidase 4 (proto-oncogene)
240321_at	3,090E-05	TCF4	Transcription factor 4
234945_at	3,090E-05	FAM54A	family with sequence similarity 54, member A
242939_at	3,090E-05	TFDP1	transcription factor Dp-1
1559045_at	3,150E-05		CDNA FLJ37541 fis, clone BRCAN2026340
221984_s_at	3,150E-05	C2orf17	chromosome 2 open reading frame 17
216978_x_at	3,180E-05	ACTL6B	Actin-like 6B
1563207_at	3,190E-05		CDNA FLJ32900 fis, clone TESTI2005492
211270_x_at	3,190E-05	PTBP1	polypyrimidine tract binding protein 1
238371_s_at	3,220E-05	EPS8	Epidermal growth factor receptor pathway substrate 8
243562_at	3,250E-05		
230634_x_at	3,280E-05	LOC113179	hypothetical protein BC011824
229573_at	3,300E-05		Transcribed locus, weakly similar to NP_009083,1 zinc finger protein 195 [Homo sapiens]
242069_at	3,410E-05	CBX5	Chromobox homolog 5 (HP1 alpha homolog, Drosophila)

216446_at	3,500E-05	GLIS1	GLIS family zinc finger 1
242565_x_at	3,560E-05		Full-length cDNA clone CS0CAP007YH21 of Thymus of Homo sapiens (human)
242073_at	3,630E-05	RALBP1	RalA binding protein 1
211546_x_at	3,820E-05	SNCA	synuclein, alpha (non A4 component of amyloid precursor)
232657_at	3,940E-05	GPR107	G protein-coupled receptor 107
225186_at	3,980E-05	RAPH1	Ras association (RalGDS/AF-6) and pleckstrin homology domains 1
229672_at	4,080E-05	C20orf44	Chromosome 20 open reading frame 44
212338_at	4,090E-05	MYO1D	myosin ID
210744_s_at	4,090E-05	IL5RA	interleukin 5 receptor, alpha
217364_x_at	4,090E-05	EIF3S1	eukaryotic translation initiation factor 3, subunit 1 alpha, 35kDa
60794_f_at	4,140E-05	ZNF587	zinc finger protein 587
1556477_a_at	4,160E-05	LOC283485	hypothetical protein LOC283485
208629_s_at	4,220E-05	HADHA	hydroxyacyl-Coenzyme A dehydrogenase/3-ketoacyl-Coenzyme A thiolase/enoyl-Coenzyme A hydratase (trifunctional protein), alpha subunit
1569894_at	4,330E-05	C14orf10	chromosome 14 open reading frame 10
227875_at	4,340E-05	KLHL13	kelch-like 13 (Drosophila)
223799_at	4,470E-05	KIAA1826	KIAA1826 protein
226901_at	4,500E-05	LOC284018	hypothetical protein LOC284018
208576_s_at	4,600E-05	HIST1H3B	histone 1, H3b
227417_at	4,640E-05	MOSC2	MOCO sulphurase C-terminal domain containing 2
207870_at	4,640E-05	AKAP9	A kinase (PRKA) anchor protein (yotiao) 9
234701_at	4,710E-05	ANKRD11	ankyrin repeat domain 11
235536_at	4,710E-05		Similar to RIKEN cDNA E030024N20 gene
1553129_at	4,710E-05	SVEP1	sushi, von Willebrand factor type A, EGF and pentraxin domain containing 1
240902_at	4,750E-05	LOC283624	Hypothetical protein LOC283624
236166_at	4,750E-05	LOC285147	hypothetical protein LOC285147
204283_at	4,870E-05	FARS2	phenylalanine-tRNA synthetase 2 (mitochondrial)
234809_at	4,930E-05	KIAA1166	KIAA1166
208408_at	4,960E-05	PTN	pleiotrophin (heparin binding growth factor 8, neurite growth-promoting factor 1)
233717_x_at	4,970E-05	SMO	Smoothened homolog (Drosophila)
218807_at	4,990E-05	VAV3	vav 3 oncogene
223750_s_at	5,070E-05	TLR10	toll-like receptor 10
234953_x_at	5,110E-05	ZNF19	zinc finger protein 19 (KOX 12)
203559_s_at	5,110E-05	ABP1	amiloride binding protein 1 (amine oxidase (copper-containing))
217328_at	5,170E-05		T cell receptor beta chain (TCRB)
220142_at	5,230E-05	HAPLN2	hyaluronan and proteoglycan link protein 2
216812_at	5,460E-05		
201712_s_at	5,510E-05	RANBP2	RAN binding protein 2
222545_s_at	5,570E-05	C10orf57	chromosome 10 open reading frame 57
226367_at	5,570E-05	JARID1A	Jumonji, AT rich interactive domain 1A (RBBP2-like)
203343_at	5,600E-05	UGDH	UDP-glucose dehydrogenase
213732_at	5,630E-05	TCF3	Transcription factor 3 (E2A immunoglobulin enhancer binding factors E12/E47)
1555807_a_at	5,730E-05	MOG	myelin oligodendrocyte glycoprotein
236372_at	5,730E-05	PANX1	Pannexin 1
224580_at	5,910E-05	SLC38A1	Solute carrier family 38, member 1
205167_s_at	5,950E-05	CDC25C	cell division cycle 25C
219232_s_at	5,990E-05	EGLN3	egl nine homolog 3 (C, elegans)
202343_x_at	6,010E-05	COX5B	cytochrome c oxidase subunit Vb
1566959_at	6,100E-05	GAB2	GRB2-associated binding protein 2
212984_at	6,160E-05	ATF2	activating transcription factor 2
214793_at	6,160E-05	DUSP7	dual specificity phosphatase 7

229839_at	6,220E-05	MGC45780	Scavenger receptor class A, member 5 (putative)
243916_x_at	6,530E-05	UBLCP1	ubiquitin-like domain containing CTD phosphatase 1
224647_at	6,570E-05	C10orf9	chromosome 10 open reading frame 9
210454_s_at	6,640E-05	KCNJ6	potassium inwardly-rectifying channel, subfamily J, member 6
1565613_at	6,710E-05		Homo sapiens, clone IMAGE:4403366
1553067_a_at	6,710E-05	GNRHR2	gonadotropin-releasing hormone (type 2) receptor 2
233302_at	6,720E-05	BCL11B	B-cell CLL/lymphoma 11B (zinc finger protein)
1561473_at	6,840E-05		CDNA clone IMAGE:5295793
1557124_at	6,850E-05		Similar to RIKEN cDNA 1110012D08
227482_at	6,850E-05	ADCK1	aarF domain containing kinase 1
219649_at	6,850E-05	ALG6	asparagine-linked glycosylation 6 homolog (yeast, alpha-1,3-glucosyltransferase)
204266_s_at	6,920E-05	CHKA	choline kinase alpha
221217_s_at	6,920E-05	A2BP1	ataxin 2-binding protein 1
239358_at	7,050E-05	FDFT1	Farnesyl-diphosphate farnesyltransferase 1
243969_at	7,140E-05	SLC24A4	solute carrier family 24 (sodium/potassium/calcium exchanger), member 4
242925_at	7,140E-05	RNF148	ring finger protein 148
1560987_a_at	7,140E-05	RBM15	RNA binding motif protein 15
208787_at	7,230E-05	MRPL3	mitochondrial ribosomal protein L3
203572_s_at	7,330E-05	TAF6	TAF6 RNA polymerase II, TATA box binding protein (TBP)-associated factor, 80kDa
214806_at	7,330E-05	BICD1	bicaudal D homolog 1 (Drosophila)
222871_at	7,440E-05	FLJ10748	Hypothetical protein FLJ10748
204662_at	7,600E-05	CP110	CP110 protein
221637_s_at	7,620E-05	MGC2477	hypothetical protein MGC2477
244515_at	7,620E-05	PSMD7	Proteasome (prosome, macropain) 26S subunit, non-ATPase, 7 (Mov34 homolog)
206215_at	7,800E-05	OPCML	opioid binding protein/cell adhesion molecule-like
224665_at	7,860E-05	C10orf104	chromosome 10 open reading frame 104
234355_s_at	7,910E-05	PTCHD2	patched domain containing 2
221476_s_at	7,910E-05	RPL15	ribosomal protein L15
204501_at	7,920E-05	NOV	nephroblastoma overexpressed gene
223905_at	7,930E-05	C16orf50	chromosome 16 open reading frame 50
232235_at	8,060E-05	C18orf4	chromosome 18 open reading frame 4
219075_at	8,060E-05	YIPF2	Yip1 domain family, member 2
225788_at	8,060E-05	C6orf153	chromosome 6 open reading frame 153
218953_s_at	8,080E-05	MGC3265	hypothetical protein MGC3265
230832_at	8,110E-05	RTF1	Rtf1, Paf1/RNA polymerase II complex component, homolog (S, cerevisiae)
231909_x_at	8,140E-05	ODF2L	outer dense fiber of sperm tails 2-like
243571_at	8,240E-05		Transcribed locus
238966_at	8,340E-05	BRUNOL4	Bruno-like 4, RNA binding protein (Drosophila)
202908_at	8,480E-05	WFS1	Wolfram syndrome 1 (wolframin)
228100_at	8,560E-05	C1orf88	chromosome 1 open reading frame 88
219875_s_at	8,560E-05	C1orf121	chromosome 1 open reading frame 121
1555839_a_at	8,560E-05	LOC152118	hypothetical gene supported by AF086445
1554250_s_at	8,560E-05	TRIM50B	tripartite motif-containing 50B
1554571_at	8,560E-05	APBB1IP	amyloid beta (A4) precursor protein-binding, family B, member 1 interacting protein
1554793_at	8,560E-05	UBE3C	ubiquitin protein ligase E3C
231910_at	8,700E-05	NUDT14	Nudix (nucleoside diphosphate linked moiety X)-type motif 14
217123_x_at	8,790E-05	PMCHL1	pro-melanin-concentrating hormone-like 1
204579_at	8,850E-05	FGFR4	fibroblast growth factor receptor 4
207015_s_at	8,850E-05	ALDH1A2	aldehyde dehydrogenase 1 family, member A2
232388_at	8,850E-05	CNTNAP4	contactin associated protein-like 4
228861_at	8,850E-05	CDS2	CDP-diacylglycerol synthase (phosphatidate cytidylyltransferase) 2

241864_x_at	8,850E-05		Transcribed locus
230198_at	8,850E-05	IDI1	Isopentenyl-diphosphate delta isomerase 1
212879_x_at	9,100E-05	PIAS4	protein inhibitor of activated STAT, 4
219583_s_at	9,100E-05	SPATA7	spermatogenesis associated 7
225061_at	9,110E-05	DNAJA4	DnaJ (Hsp40) homolog, subfamily A, member 4
217108_at	9,140E-05		
214329_x_at	9,140E-05	TNFSF10	Tumor necrosis factor (ligand) superfamily, member 10 ; Tumor necrosis factor (ligand) superfamily, member 10
202342_s_at	9,200E-05	TRIM2	tripartite motif-containing 2
231163_at	9,260E-05	C1orf111	chromosome 1 open reading frame 111
1558686_at	9,390E-05		Full length insert cDNA YP99D02
213941_x_at	9,400E-05	RPS7	ribosomal protein S7
240826_at	9,480E-05		Transcribed locus
209136_s_at	9,480E-05	USP10	ubiquitin specific peptidase 10
215309_at	9,550E-05		MRNA; cDNA DKFZp686H0940 (from clone DKFZp686H0940)
43544_at	9,550E-05	THRAP5	thyroid hormone receptor associated protein 5
209452_s_at	9,550E-05	VTI1B	vesicle transport through interaction with t-SNAREs homolog 1B (yeast)
239410_at	9,690E-05		
221921_s_at	1,020E-04	IGSF4B	immunoglobulin superfamily, member 4B
1554273_a_at	1,020E-04	LRAP	leukocyte-derived arginine aminopeptidase
226907_at	1,030E-04	PPP1R14C	protein phosphatase 1, regulatory (inhibitor) subunit 14C
204893_s_at	1,030E-04	ZFYVE9	zinc finger, FYVE domain containing 9
242240_at	1,030E-04	PTK2	PTK2 protein tyrosine kinase 2
203246_s_at	1,040E-04	TUSC4	tumor suppressor candidate 4
202424_at	1,040E-04	MAP2K2	mitogen-activated protein kinase kinase 2
1566402_at	1,050E-04	RNU68	RNA, U68 small nucleolar
201627_s_at	1,050E-04	INSIG1	insulin induced gene 1
238979_at	1,070E-04		
209910_at	1,080E-04	SLC25A16	solute carrier family 25 (mitochondrial carrier; Graves disease autoantigen), member 16
1566459_at	1,090E-04		CDNA clone IMAGE:4801197
235309_at	1,090E-04		CDNA clone IMAGE:4140029
223648_s_at	1,110E-04	FGFRL1	fibroblast growth factor receptor-like 1
1564272_a_at	1,120E-04	KLHDC1	kelch domain containing 1
206403_at	1,150E-04	ZNF536	zinc finger protein 536
1554908_at	1,160E-04	HYDIN	hydrocephalus inducing
212976_at	1,160E-04	LRRC8B	leucine rich repeat containing 8 family, member B
201510_at	1,160E-04	ELF3	E74-like factor 3 (ets domain transcription factor, epithelial-specific)
244495_x_at	1,160E-04	C18orf45	chromosome 18 open reading frame 45
236629_at	1,190E-04	C1orf69	chromosome 1 open reading frame 69
1564937_at	1,190E-04	KIAA1002	KIAA1002 protein
1553793_a_at	1,200E-04	KIAA1109	KIAA1109
214032_at	1,200E-04	ZAP70	zeta-chain (TCR) associated protein kinase 70kDa
238572_at	1,200E-04	MGC16211	hypothetical protein MGC16211
228891_at	1,210E-04	LOC349236	hypothetical protein LOC349236
230297_x_at	1,210E-04	SYNGAP1	synaptic Ras GTPase activating protein 1 homolog (rat)
214027_x_at	1,230E-04	DES ; FAM48A	desmin ; family with sequence similarity 48, member A
225377_at	1,270E-04	C9orf86	chromosome 9 open reading frame 86
243903_at	1,290E-04		Transcribed locus
227436_at	1,300E-04	ZA20D1	Zinc finger, A20 domain containing 1
1560791_at	1,300E-04		Homo sapiens, clone IMAGE:5221301, mRNA
1562683_a_at	1,320E-04	LOC285547	hypothetical protein LOC285547
1559952_x_at	1,320E-04	LOC401449 ; FAM66C ; FAM66E	hypothetical LOC401449 ; family with sequence similarity 66, member C ; family with sequence similarity 66, member E
211296_x_at	1,360E-04	UBC	ubiquitin C

207904_s_at	1,400E-04	LNPEP	leucyl/cystinyl aminopeptidase
1561589_a_at	1,410E-04	NBEAL1	neurobeachin-like 1
206382_s_at	1,430E-04	BDNF	brain-derived neurotrophic factor
220927_s_at	1,470E-04	HPSE2	heparanase 2
212706_at	1,470E-04	RASA4 ; FLJ21767	RAS p21 protein activator 4 ; hypothetical protein FLJ21767
1557347_at	1,480E-04	MCPH1	Microcephaly, primary autosomal recessive 1
216704_at	1,500E-04	TBCA	Tubulin-specific chaperone a
233815_at	1,500E-04	NAALAD2	N-acetylated alpha-linked acidic dipeptidase 2
217821_s_at	1,500E-04	WBP11	WW domain binding protein 11
233538_s_at	1,500E-04	CYBB	Cytochrome b-245, beta polypeptide (chronic granulomatous disease)
41660_at	1,520E-04	CELSR1	cadherin, EGF LAG seven-pass G-type receptor 1 (flamingo homolog, Drosophila)
241686_x_at	1,520E-04		Transcribed locus
213736_at	1,530E-04	COX5B	Cytochrome c oxidase subunit Vb
237973_at	1,540E-04		Transcribed locus
212083_at	1,540E-04	TEX261	testis expressed sequence 261
227248_at	1,540E-04	FLJ21019	hypothetical protein FLJ21019
1565935_at	1,540E-04	LOC91431	prematurely terminated mRNA decay factor-like
205906_at	1,560E-04	FOXJ1	forkhead box J1
203049_s_at	1,570E-04	KIAA0372	KIAA0372
240401_at	1,610E-04	AATF	Apoptosis antagonizing transcription factor
1556734_at	1,610E-04	LMO7	LIM domain 7
231203_at	1,630E-04	LOC389786	similar to ribosomal protein L7
1557208_at	1,640E-04	LOC219731	hypothetical protein LOC219731
202918_s_at	1,640E-04	PREI3	preimplantation protein 3
231227_at	1,640E-04	WNT5A	Wingless-type MMTV integration site family, member 5A
210100_s_at	1,640E-04	ABCA2	ATP-binding cassette, sub-family A (ABC1), member 2
218355_at	1,650E-04	KIF4A	kinesin family member 4A
232139_s_at	1,650E-04	KIAA1919	KIAA1919
214738_s_at	1,680E-04	NEK9	NIMA (never in mitosis gene a)- related kinase 9
235456_at	1,700E-04	HIST1H2BD	Histone 1, H2bd
236255_at	1,700E-04	KIAA1909	KIAA1909 protein
1568844_at	1,700E-04		CDNA clone IMAGE:4826156
234880_x_at	1,770E-04	KRTAP1-3	keratin associated protein 1-3
1555734_x_at	1,790E-04	AP1S3	adaptor-related protein complex 1, sigma 3 subunit
220173_at	1,800E-04	C14orf45	chromosome 14 open reading frame 45
232777_s_at	1,800E-04	C6orf118	chromosome 6 open reading frame 118
202095_s_at	1,800E-04	BIRC5	baculoviral IAP repeat-containing 5 (survivin)
203164_at	1,830E-04	SLC33A1	solute carrier family 33 (acetyl-CoA transporter), member 1
1563104_at	1,830E-04	RAB11FIP3	RAB11 family interacting protein 3 (class II)
244353_s_at	1,830E-04		
229580_at	1,830E-04		Transcribed locus
223738_s_at	1,830E-04	PGM2	phosphoglucomutase 2
236363_at	1,830E-04	LOC285378	hypothetical protein LOC285378
202335_s_at	1,830E-04	UBE2B	ubiquitin-conjugating enzyme E2B (RAD6 homolog)
217330_at	1,850E-04	DISC1	disrupted in schizophrenia 1
205265_s_at	1,880E-04	APEG1	aortic preferentially expressed gene 1
208502_s_at	1,920E-04	PITX1	paired-like homeodomain transcription factor 1
227667_at	1,920E-04	CUEDC1	CUE domain containing 1
242750_at	1,940E-04	MMAA	Methylmalonic aciduria (cobalamin deficiency) cblA type
215603_x_at	1,950E-04	GGT2	gamma-glutamyltransferase 2
201058_s_at	1,950E-04	MYL9	myosin, light polypeptide 9, regulatory
208373_s_at	1,970E-04	P2RY6	pyrimidinergic receptor P2Y, G-protein coupled, 6
213814_s_at	1,980E-04	SNTB2	Syntrophin, beta 2 (dystrophin-associated protein A1, 59kDa, basic component 2)

214741_at	1,980E-04	ZNF131	zinc finger protein 131 (clone pHZ-10)
211048_s_at	1,990E-04	PDIA4	protein disulfide isomerase family A, member 4 ; protein disulfide isomerase family A, member 4
215422_at	2,000E-04		CDNA FLJ31636 fis, clone NT2RI2003481
208933_s_at	2,030E-04	LGALS8	lectin, galactoside-binding, soluble, 8 (galectin 8)
206051_at	2,040E-04	ELAVL4	ELAV (embryonic lethal, abnormal vision, Drosophila)-like 4 (Hu antigen D)
1562982_at	2,060E-04		Homo sapiens, clone IMAGE:5541085, mRNA
220946_s_at	2,060E-04	HYPB	huntingtin interacting protein B
1556579_s_at	2,080E-04	IGSF10	immunoglobulin superfamily, member 10
236434_at	2,100E-04	PES1	pescadillo homolog 1, containing BRCT domain (zebrafish)
225780_at	2,120E-04	RSC1A1	regulatory solute carrier protein, family 1, member 1
1565595_at	2,160E-04	SLC6A16	Solute carrier family 6, member 16
204405_x_at	2,170E-04	HSA9761	dimethyladenosine transferase
210268_at	2,190E-04	NFX1	nuclear transcription factor, X-box binding 1
221733_s_at	2,210E-04	GPATC4	G patch domain containing 4
1570338_at	2,230E-04	MGC25181	Hypothetical protein MGC25181 ; CDNA FLJ26792 fis, clone PRS04865
239837_at	2,230E-04	ADAM11	ADAM metallopeptidase domain 11
243663_at	2,230E-04		
211805_s_at	2,230E-04	SLC8A1	solute carrier family 8 (sodium/calcium exchanger), member 1
232256_s_at	2,240E-04	LOC401321	hypothetical LOC401321
227338_at	2,240E-04	LOC440983	hypothetical gene supported by BC066916
237576_x_at	2,270E-04	LOC440422	LOC440422
217920_at	2,270E-04	MAN1A2	mannosidase, alpha, class 1A, member 2
227552_at	2,320E-04	SEPT1	septin 1
229411_at	2,320E-04	PNCK	pregnancy upregulated non-ubiquitously expressed CaM kinase
206457_s_at	2,330E-04	DIO1	deiodinase, iodothyronine, type I
208416_s_at	2,330E-04	SPTB	spectrin, beta, erythrocytic (includes spherocytosis, clinical type I)
208994_s_at	2,350E-04	PPIG	peptidyl-prolyl isomerase G (cyclophilin G)
1552607_at	2,350E-04	CXorf52	chromosome X open reading frame 52
200843_s_at	2,360E-04	EPRS	glutamyl-prolyl-tRNA synthetase
1564402_at	2,360E-04	LOC146795	hypothetical protein LOC146795
207314_x_at	2,450E-04	KIR3DL2	killer cell immunoglobulin-like receptor, three domains, long cytoplasmic tail, 2
207896_s_at	2,460E-04	DLEC1	deleted in lung and esophageal cancer 1
205378_s_at	2,530E-04	ACHE	acetylcholinesterase (YT blood group)
1569264_at	2,560E-04	LOC400655	hypothetical gene supported by BC013370; BC034583
201569_s_at	2,570E-04	SAMM50	sorting and assembly machinery component 50 homolog (S, cerevisiae)
226739_at	2,600E-04	RNF169	ring finger protein 169
1559474_at	2,630E-04	APEG1	aortic preferentially expressed gene 1
1570444_at	2,640E-04		Similar to hypothetical protein
238335_at	2,640E-04	LOC134218	DnaJ homology subfamily A member 5
1560774_at	2,710E-04		CDNA FLJ26469 fis, clone KDN04330
1560290_at	2,750E-04		CDNA FLJ26132 fis, clone TMS03580
208359_s_at	2,750E-04	KCNJ4	potassium inwardly-rectifying channel, subfamily J, member 4
209836_x_at	2,750E-04	BOLA2	bolA-like 2 (E, coli)
203632_s_at	2,750E-04	GPRC5B	G protein-coupled receptor, family C, group 5, member B
213599_at	2,750E-04	OIP5	Opa interacting protein 5
232190_x_at	2,750E-04	LOC115110 ; LOC440555	hypothetical protein LOC115110 ; hypothetical gene supported by AK021767; AL359943
220864_s_at	2,750E-04	NDUFA13	NADH dehydrogenase (ubiquinone) 1 alpha subcomplex, 13
