# Supplementary material for: Gene Expression Analysis in Ovarian Cancer – Faults and Hints from DNA Microarray Study
Source: Front Oncol. 2014 Jan 28;4:6. doi: 10.3389/fonc.2014.00006 (PMC3904181; doi:10.3389/fonc.2014.00006)
Supplement: Supplementary file 3 [file 61993_Lisowska_DataSheet3.PDF]

Histological type of ovarian cancer

1. Hierarchical clustering: Dendrogram shows the relations between samples of different histological type. The majority of clear cell and endometrioid tumors are clustered in a separate branch on the left side of dendrogram. Serous and undifferentiated tumors represent the majority in the right branch. Heat map shows relative intensity of genes expression. No specific gene expression pattern is visible corresponding to the given histological type.

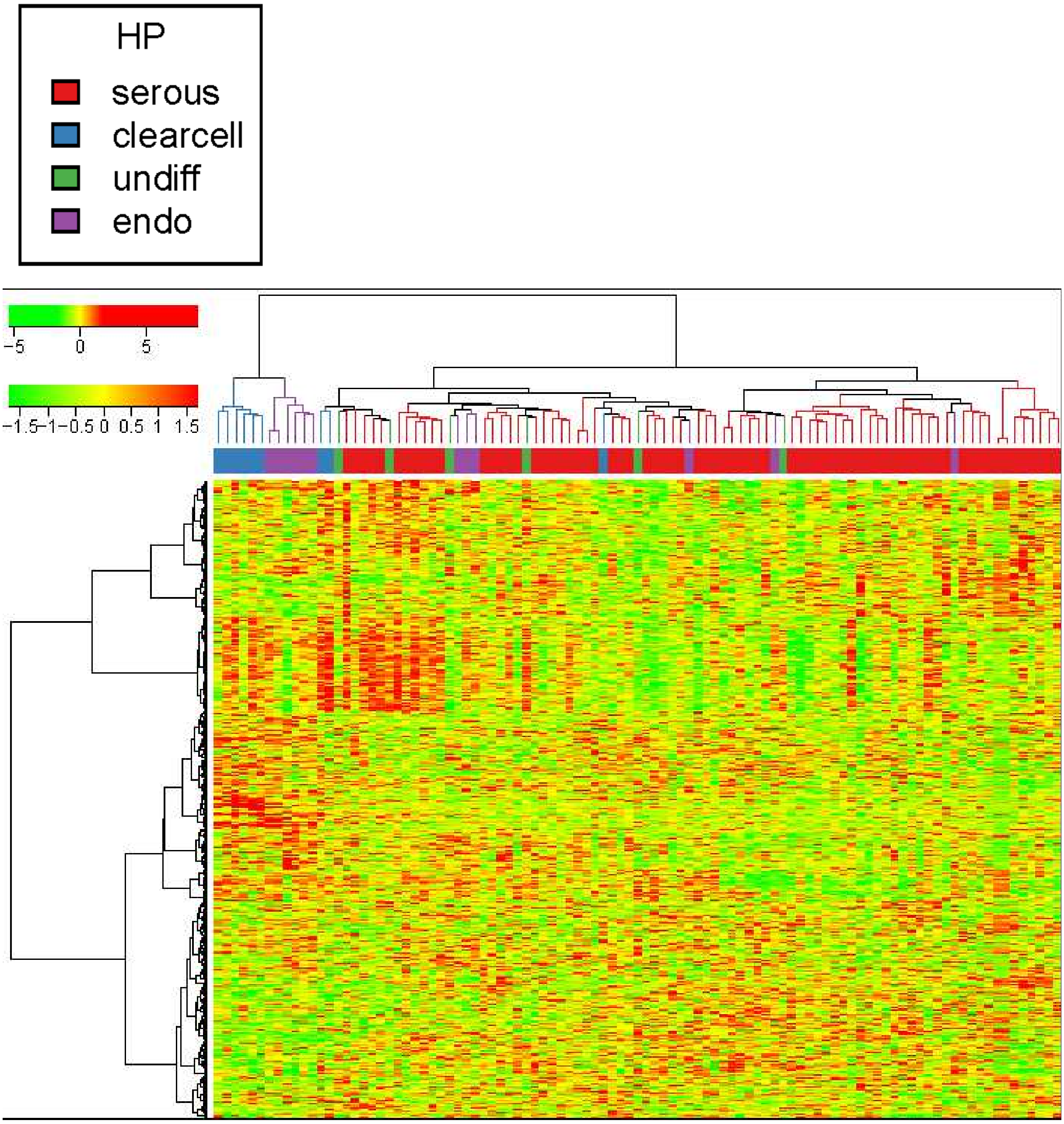

2. Signaling pathways (according to Biocarta repository) with significantly changed gene expression between ovarian cancers of distinct histology. Hotelling test was used to analyze annotated genes selected by ANOVA in comparison of 4 histological types of ovarian cancer (serous, undifferentiated, clear cell and endometrioid.

	**Signaling pathway (Biocarta)**	**Signaling pathway name**	**p-value**
1	h_nuclearRsPathway	Nuclear Receptors in Lipid Metabolism and Toxicity	p < 1e-07
2	h_EfpPathway	Estrogen-responsive protein Efp controls cell cycle and breast tumors growth	p < 1e-07
3	h_mta3Pathway	Downregulated of MTA-3 in ER-negative Breast Tumors	p < 1e-07
4	h_fxrPathway	FXR and LXR Regulation of Cholesterol Metabolism	p < 1e-07
5	h_pelp1Pathway	Pelp1 Modulation of Estrogen Receptor Activity	p < 1e-07
6	h_tidPathway	Chaperones modulate interferon Signaling Pathway	p < 1e-07
7	h_her2Pathway	Role of ERBB2 in Signal Transduction and Oncology	p < 1e-07
8	h_tertpathway	Overview of telomerase protein component gene hTert Transcriptional Regulation	p < 1e-07
9	h_HivnefPathway	HIV-I Nef: negative effector of Fas and TNF	p < 1e-07
10	h_carm-erPathway	CARM1 and Regulation of the Estrogen Receptor	p < 1e-07

11	h_il10Pathway	IL-10 Anti-inflammatory Signaling Pathway	p < 1e-07
12	h_no2il12Pathway	NO2-dependent IL 12 Pathway in NK cells	p < 1e-07
13	h_mrpPathway	Multi-Drug Resistance Factors	p < 1e-07
14	h_metPathway	Signaling of Hepatocyte Growth Factor Receptor	p < 1e-07
15	h_methioninepathway	Catabolic Pathways for Methionine, Isoleucine, Threonine and Valine	p < 1e-07
16	h_gsk3Pathway	Inactivation of Gsk3 by AKT causes accumulation of b-catenin in Alveolar Macrophages	p < 1e-07
17	h_cblPathway	CBL mediated ligand-induced downregulation of EGF receptors	p < 1e-07
18	h_hdacPathway	Control of skeletal myogenesis by HDAC & calcium/calmodulin-dependent kinase (CaMK)	p < 1e-07
19	h_deathPathway	Induction of apoptosis through DR3 and DR4/5 Death Receptors	p < 1e-07
20	h_prc2Pathway	The PRC2 Complex Sets Long-term Gene Silencing Through Modification of Histone Tails	p < 1e-07
21	h_il1rPathway	Signal transduction through IL1R	p < 1e-07
22	h_bArrestinPathway	ß-arrestins in GPCR Desensitization	p < 1e-07
23	h_mPRPathway	How Progesterone Initiates the Oocyte Maturation	p < 1e-07
24	h_mitochondriaPathway	Role of Mitochondria in Apoptotic Signaling	p < 1e-07
25	h_bArrestin-srcPathway	Roles of ß-arrestin-dependent Recruitment of Src Kinases in GPCR Signaling	p < 1e-07
26	h_crebPathway	Transcription factor CREB and its extracellular signals	p < 1e-07
27	h_barr-mapkPathway	Role of ß-arrestins in the activation and targeting of MAP kinases	p < 1e-07
28	h_p53hypoxiaPathway	Hypoxia and p53 in the Cardiovascular system	p < 1e-07
29	h_il6Pathway	IL 6 signaling pathway	p < 1e-07
30	h_extrinsicPathway	Extrinsic Prothrombin Activation Pathway	p < 1e-07
31	h_ctbp1Pathway	SUMOylation as a mechanism to modulate CtBP-dependent gene responses	p < 1e-07
32	h_tffPathway	Trefoil Factors Initiate Mucosal Healing	p < 1e-07
33	h_prionPathway	Prion Pathway	p < 1e-07
34	h_erythPathway	Erythrocyte Differentiation Pathway	p < 1e-07
35	h_freePathway	Free Radical Induced Apoptosis	p < 1e-07
36	h_ghrelinPathway	Ghrelin: Regulation of Food Intake and Energy Homeostasis	p < 1e-07
37	h_nthiPathway	NFkB activation by Nontypeable Hemophilus influenzae	p < 1e-07
38	h_LDLpathway	Low-density lipoprotein (LDL) pathway during atherogenesis	p < 1e-07
39	h_edg1Pathway	Phospholipids as signalling intermediaries	p < 1e-07
40	h_cpsfPathway	Polyadenylation of mRNA	p < 1e-07
41	h_argininecPathway	Catabolic Pathways for Arginine , Histidine, Glutamate, Glutamine, and Proline	p < 1e-07
42	h_vdrPathway	Control of Gene Expression by Vitamin D Receptor	p < 1e-07
43	h_nos1Pathway	Nitric Oxide Signaling Pathway	p < 1e-07
44	h_amiPathway	Acute Myocardial Infarction	p < 1e-07
45	h_aktPathway	AKT Signaling Pathway	p < 1e-07
46	h_stemPathway	Regulation of hematopoiesis by cytokines	p < 1e-07
47	h_pparaPathway	Mechanism of Gene Regulation by Peroxisome Proliferators via PPARa(alpha)	p < 1e-07
48	h_tnfr1Pathway	TNFR1 Signaling Pathway	p < 1e-07
49	h_g1Pathway	Cell Cycle: G1/S Check Point	p < 1e-07
50	h_pitx2Pathway	Multi-step Regulation of Transcription by Pitx2	p < 1e-07
51	h_hcmvPathway	Human Cytomegalovirus and Map Kinase Pathways	p < 1e-07
52	h_ranbp2Pathway	Sumoylation by RanBP2 Regulates Transcriptional Repression	p < 1e-07
53	h_rac1Pathway	Rac 1 cell motility signaling pathway	p < 1e-07
54	h_nfatPathway	NFAT and Hypertrophy of the heart (Transcription in the broken heart)	p < 1e-07
55	h_sppaPathway	Aspirin Blocks Signaling Pathway Involved in Platelet Activation	p < 1e-07
56	h_keratinocytePathway	Keratinocyte Differentiation	p < 1e-07
57	h_melanocytepathway	Melanocyte Development and Pigmentation Pathway	p < 1e-07
58	h_fasPathway	FAS signaling pathway (CD95)	p < 1e-07
59	h_eicosanoidPathway	Eicosanoid Metabolism	p < 1e-07
60	h_tgfbPathway	TGF beta signaling pathway	p < 1e-07
61	h_ndkDynaminPathway	Endocytotic role of NDK, Phosphins and Dynamin	p < 1e-07
62	h_IL12Pathway	IL12 and Stat4 Dependent Signaling Pathway in Th1 Development	p < 1e-07
63	h_ucalpainPathway	uCalpain and friends in Cell spread	p < 1e-07
64	h_alkPathway	ALK in cardiac myocytes	p < 1e-07
65	h_bcrPathway	BCR Signaling Pathway	p < 1e-07
66	h_raccPathway	Ion Channels and Their Functional Role in Vascular Endothelium	p < 1e-07
67	h_gpcrPathway	Signaling Pathway from G-Protein Families	p < 1e-07
68	h_41bbPathway	The 4-1BB-dependent immune response	p < 1e-07
69	h_fMLPpathway	fMLP induced chemokine gene expression in HMC-1 cells	p < 1e-07
70	h_stressPathway	TNF/Stress Related Signaling	p < 1e-07
71	h_arenrf2Pathway	Oxidative Stress Induced Gene Expression Via Nrf2	p < 1e-07
72	h_nktPathway	Selective expression of chemokine receptors during T-cell polarization	p < 1e-07
73	h_soddPathway	SODD/TNFR1 Signaling Pathway	p < 1e-07
74	h_cremPathway	Regulation of Spermatogenesis by CREM	p < 1e-07

75	h_caspasePathway	Caspase Cascade in Apoptosis	p < 1e-07
76	h_biopeptidesPathway	Bioactive Peptide Induced Signaling Pathway	p < 1e-07
77	h_appPathway	Generation of amyloid b-peptide by PS1	1e-07
78	h_etsPathway	METS affect on Macrophage Differentiation	1e-07
79	h_gsPathway	Activation of cAMP-dependent protein kinase, PKA	1e-07
80	h_tollPathway	Toll-Like Receptor Pathway	2e-07
81	h_mbdPathway	Mechanisms of transcriptional repression by DNA methylation	2e-07
82	h_rabPathway	Rab GTPases Mark Targets In The Endocytotic Machinery	2e-07
83	h_eponfkbPathway	Erythropoietin mediated neuroprotection through NF-kB	2e-07
84	h_chrebpPathway	ChREBP regulation by carbohydrates and cAMP	2e-07
85	h_hesPathway	Segmentation Clock	2e-07
86	h_p38mapkPathway	p38 MAPK Signaling Pathway	3e-07
87	h_g2Pathway	Cell Cycle: G2/M Checkpoint	3e-07
88	h_cellcyclePathway	Cyclins and Cell Cycle Regulation	3e-07
89	h_monocytePathway	Monocyte and its Surface Molecules	3e-07
90	h_Par1Pathway	Thrombin signaling and protease-activated receptors	4e-07
91	h_ranPathway	Cycling of Ran in nucleocytoplasmic transport	5e-07
92	h_lymphocytePathway	Adhesion Molecules on Lymphocyte	6e-07
93	h_p35alzheimersPathway	Deregulation of CDK5 in Alzheimers Disease	6e-07
94	h_gabaPathway	Gamma-aminobutyric Acid Receptor Life Cycle	6e-07
95	h_CCR3Pathway	CCR3 signaling in Eosinophils	6e-07
96	h_npcPathway	Mechanism of Protein Import into the Nucleus	7e-07
97	h_erkPathway	Erk1/Erk2 Mapk Signaling pathway	1e-06
98	h_cytokinePathway	Cytokine Network	1.2e-06
99	h_plcdPathway	Phospholipase C d1 in phospholipid associated cell signaling	1.2e-06
100	h_ppargPathway	Role of PPAR-gamma Coactivators in Obesity and Thermogenesis	1.4e-06
101	h_SARSpathway	SARS Coronavirus Protease	1.4e-06
102	h_igf1rPathway	Multiple antiapoptotic pathways from IGF-1R signaling lead to BAD phosphorylation	1.5e-06
103	h_fibrinolysisPathway	Fibrinolysis Pathway	1.5e-06
104	h_ahspPathway	Hemoglobin@	1.7e-06
105	h_eifPathway	Eukaryotic protein translation	1.8e-06
106	h_ck1Pathway	Regulation of ck1/cdk5 by type 1 glutamate receptors	2e-06
107	h_il2Pathway	IL 2 signaling pathway	2.3e-06
108	h_il2rbPathway	IL-2 Receptor Beta Chain in T cell Activation	2.4e-06
109	h_AcetaminophenPathway	Mechanism of Acetaminophen Activity and Toxicity	2.7e-06
110	h_skp2e2fPathway	E2F1 Destruction Pathway	2.9e-06
111	h_RacCycDPathway	Influence of Ras and Rho proteins on G1 to S Transition	3.8e-06
112	h_integrinPathway	Integrin Signaling Pathway	3.9e-06
113	h_cxcr4Pathway	CXCR4 Signaling Pathway	4.8e-06
114	h_rhoPathway	Rho cell motility signaling pathway	5e-06
115	h_cdMacPathway	Cadmium induces DNA synthesis and proliferation in macrophages	5e-06
116	h_tcytotoxicPathway	T Cytotoxic Cell Surface Molecules	5.4e-06
117	h_insulinPathway	Insulin Signaling Pathway	6.2e-06
118	h_cdk5Pathway	Phosphorylation of MEK1 by cdk5/p35 down regulates the MAP kinase pathway	6.3e-06
119	h_thelperPathway	T Helper Cell Surface Molecules	6.4e-06
120	h_ps1Pathway	Presenilin action in Notch and Wnt signaling	6.5e-06
121	h_parkinPathway	Role of Parkin in the Ubiquitin-Proteasomal Pathway	7.3e-06
122	h_carm1Pathway	Transcription Regulation by Methyltransferase of CARM1	7.3e-06
123	h_pdgfPathway	PDGF Signaling Pathway	8.5e-06
124	h_pyk2Pathway	Links between Pyk2 and Map Kinases	8.7e-06
125	h_longevityPathway	The IGF-1 Receptor and Longevity	8.7e-06
126	h_erk5Pathway	Role of Erk5 in Neuronal Survival	8.8e-06
127	h_badPathway	Regulation of BAD phosphorylation	9.4e-06
128	h_tall1Pathway	TACI and BCMA stimulation of B cell immune responses.	1.14e-05
129	h_eif4Pathway	Regulation of eIF4e and p70 S6 Kinase	1.16e-05
130	h_Lis1Pathway	Lissencephaly gene (LIS1) in neuronal migration and development	1.19e-05
131	h_reelinPathway	Reelin Signaling Pathway	1.24e-05
132	h_neutrophilPathway	Neutrophil and Its Surface Molecules	1.5e-05
133	h_rarrxrPathway	Nuclear receptors coordinate the activities of chromatin remodeling complexes and coactivators to facilitate initiation of transcription in carcinoma cells	1.61e-05
134	h_hifPathway	Hypoxia-Inducible Factor in the Cardiovascular System	1.65e-05
135	h_fcer1Pathway	Fc Epsilon Receptor I Signaling in Mast Cells	1.72e-05
136	h_mef2dPathway	Role of MEF2D in T-cell Apoptosis	1.9e-05
137	h_plcePathway	Phospholipase C-epsilon pathway	2.2e-05
138	h_pmlPathway	Regulation of transcriptional activity by PML	2.21e-05
139	h_PDZsPathway	Synaptic Proteins at the Synaptic Junction	2.35e-05
140	h_HuntingtonPathway	Inhibition of Huntington@	2.38e-05

141	h_hSWI-SNFpathway	Chromatin Remodeling by hSWI/SNF ATP-dependent Complexes	2.69e-05
142	h_agpcrPathway	Attenuation of GPCR Signaling	3.08e-05
143	h_erbB4pathway	g-Secretase mediated ErbB4 Signaling Pathway	3.12e-05
144	h_ace2Pathway	Angiotensin-converting enzyme 2 regulates heart function	3.18e-05
145	h_ghPathway	Growth Hormone Signaling Pathway	3.53e-05
146	h_dspPathway	Regulation of MAP Kinase Pathways Through Dual Swoistość Phosphatases	3.63e-05
147	h_tsp1Pathway	TSP-1 Induced Apoptosis in Microvascular Endothelial Cell	3.85e-05
148	h_il4Pathway	IL 4 signaling pathway	3.99e-05
149	h_malatePathway	Malate-aspartate shuttle	4.26e-05
150	h_cftrPathway	Cystic fibrosis transmembrane conductance regulator (CFTR) and beta 2 adrenergic receptor (b2AR) pathway	4.95e-05
151	h_rnaPathway	Double Stranded RNA Induced Gene Expression	5.73e-05
152	h_nkcellsPathway	Ras-Independent pathway in NK cell-mediated cytotoxicity	6e-05
153	h_arapPathway	ADP-Ribosylation Factor	6.29e-05
154	h_no1Pathway	Actions of Nitric Oxide in the Heart	7.48e-05
155	h_fosbPathway	FOSB gene expression and drug abuse	7.56e-05
156	h_mTORPathway	mTOR Signaling Pathway	7.68e-05
157	h_MITRPathway	Signal Dependent Regulation of Myogenesis by Corepressor MITR	8.63e-05
158	h_leptinPathway	Reversal of Insulin Resistance by Leptin	0.0001088
159	h_eradPathway	ER–associated degradation (ERAD) Pathway	0.0001104
160	h_wntPathway	WNT Signaling Pathway	0.0001108
161	h_cell2cellPathway	Cell to Cell Adhesion Signaling	0.000111
162	h_th1th2Pathway	Th1/Th2 Differentiation	0.0001113
163	h_ceramidePathway	Ceramide Signaling Pathway	0.0001216
164	h_GATA3pathway	GATA3 participate in activating the Th2 cytokine genes expression	0.0001313
165	h_il17Pathway	IL 17 Signaling Pathway	0.0001425
166	h_tercPathway	Overview of telomerase RNA component gene hTerc Transcriptional Regulation	0.0001745
167	h_ephA4Pathway	Eph Kinases and ephrins support platelet aggregation	0.0002158
168	h_asbcellPathway	Antigen Dependent B Cell Activation	0.0003102
169	h_bbcellPathway	Bystander B Cell Activation	0.0003102
170	h_pparPathway	Basic mechanism of action of PPARa, PPARb(d) and PPARg and effects on gene expression	0.0003166
171	h_compPathway	Complement Pathway	0.0003325
172	h_myosinPathway	PKC-catalyzed phosphorylation of inhibitory phosphoprotein of myosin phosphatase	0.0003334
173	h_spryPathway	Sprouty regulation of tyrosine kinase signals	0.0003356
174	h_s1pPathway	SREBP control of lipid synthesis	0.0003403
175	h_dreampathway	Repression of Pain Sensation by the Transcriptional Regulator DREAM	0.000343
176	h_iresPathway	Internal Ribosome entry pathway	0.0003444
177	h_egfr_smrtePathway	Map Kinase Inactivation of SMRT Corepressor	0.0003688
178	h_sam68Pathway	Regulation of Splicing through Sam68	0.0003819
179	h_vobesityPathway	Visceral Fat Deposits and the Metabolic Syndrome	0.0003925
180	h_chemicalPathway	Apoptotic Signaling in Response to DNA Damage	0.0005135
181	h_stat3Pathway	Stat3 Signaling Pathway	0.0005499
182	h_plcPathway	Phospholipase C Signaling Pathway	0.0005541
183	h_agrPathway	Agrin in Postsynaptic Differentiation	0.0005628
184	h_atmPathway	ATM Signaling Pathway	0.0006065
185	h_dcPathway	Dendritic cells in regulating TH1 and TH2 Development	0.0006583
186	h_igf1Pathway	IGF-1 Signaling Pathway	0.0007213
187	h_plk3Pathway	Regulation of cell cycle progression by Plk3	0.0007266
188	h_smPathway	Spliceosomal Assembly	0.0009198
189	h_ptenPathway	PTEN dependent cell cycle arrest and apoptosis	0.0009466
190	h_atrbrcaPathway	Role of BRCA1, BRCA2 and ATR in Cancer Susceptibility	0.0009696
191	h_actinYPathway	Y branching of actin filaments	0.0009827
192	h_cdc42racPathway	Role of PI3K subunit p85 in regulation of Actin Organization and Cell Migration	0.0010103
193	h_alternativePathway	Alternative Complement Pathway	0.001019
194	h_CSKPathway	Activation of Csk by cAMP-dependent Protein Kinase Inhibits Signaling through the T Cell Receptor	0.0015016
195	h_At1rPathway	Angiotensin II mediated activation of JNK Pathway via Pyk2 dependent signaling	0.0015491
196	h_il5Pathway	IL 5 Signaling Pathway	0.0015821
197	h_gcrPathway	Corticosteroids and cardioprotection	0.001719
198	h_hsp27Pathway	Stress Induction of HSP Regulation	0.0018028
199	h_granulocytesPathwa y	Adhesion and Diapedesis of Granulocytes	0.0018588
200	h_tob1Pathway	Role of Tob in T-cell activation	0.001871
201	h_pepiPathway	Proepithelin Conversion to Epithelin and Wound Repair Control	0.0019212
202	h_igf1mtorpathway	Skeletal muscle hypertrophy is regulated via AKT/mTOR pathway	0.0019464
203	h_tcrPathway	T Cell Receptor Signaling Pathway	0.0025311

204	h_TPOPathway	TPO Signaling Pathway	0.0025358
205	h_arfPathway	Tumor Suppressor Arf Inhibits Ribosomal Biogenesis	0.0026546
206	h_ranklPathway	Bone Remodelling	0.002829
207	h_ctla4Pathway	The Co-Stimulatory Signal During T-cell Activation	0.0028418
208	h_setPathway	Granzyme A mediated Apoptosis Pathway	0.0028832
209	h_plateletAppPathway	Platelet Amyloid Precursor Protein Pathway	0.0028898
210	h_malPathway	Role of MAL in Rho-Mediated Activation of SRF	0.0034369
211	h_reckPathway	Inhibition of Matrix Metalloproteinases	0.0035269
212	h_p27Pathway	Regulation of p27 Phosphorylation during Cell Cycle Progression	0.0037042
213	h_GrupaicPathway	Grupaical Complement Pathway	0.0037152
214	h_bard1Pathway	BRCA1-dependent Ub-ligase activity	0.0037503
215	h_ecmPathway	Erk and PI-3 Kinase Are Necessary for Collagen Binding in Corneal Epithelia	0.0038796
216	h_ctlPathway	CTL mediated immune response against target cells	0.0040321
217	h_aMANpathway	Steps in the Glycosylation of Mammalian N-linked Oligosaccarides	0.0047155

3. Signaling pathways (according to Biocarta repository) with significantly changed gene expression between ovarian cancers of distinct histology. Least square (LS) and Kolomogorov-Smirnoff tests (KS) were used to analyze annotated genes selected by ANOVA in comparison of 4 histological types of ovarian cancer.

	**Signalling pathways (Biocarta)**	**Signalling pathway name**	**p-value LS**	**p-value KS**
1	h_mta3Pathway	Downregulated of MTA-3 in ER-negative Breast Tumors	1e-05	0.0064106
2	h_nuclearRsPathway	Nuclear Receptors in Lipid Metabolism and Toxicity	1e-05	0.0293894
3	h_mrpPathway	Multi-Drug Resistance Factors	0.0003728	0.0975121
4	h_HivnefPathway	HIV-I Nef: negative effector of Fas and TNF	0.0008438	0.0433122
5	h_extrinsicPathway	Extrinsic Prothrombin Activation Pathway	0.0012624	0.1416955
6	h_EfpPathway	Estrogen-responsive protein Efp controls cell cycle and breast tumors growth	0.0017387	0.0048034
7	h_amiPathway	Acute Myocardial Infarction	0.0022952	0.1734852
8	h_prionPathway	Prion Pathway	0.0030394	0.1665524
9	h_deathPathway	Induction of apoptosis through DR3 and DR4/5 Death Receptors	0.0044739	0.3308774
10	h_il2rbPathway	IL-2 Receptor Beta Chain in T cell Activation	0.004894	0.0003221
11	h_AcetaminophenPathway	Mechanism of Acetaminophen Activity and Toxicity	0.0285458	0.003447
