# Supplementary material for: Gene Expression Analysis in Ovarian Cancer – Faults and Hints from DNA Microarray Study
Source: Front Oncol. 2014 Jan 28;4:6. doi: 10.3389/fonc.2014.00006 (PMC3904181; doi:10.3389/fonc.2014.00006)
Supplement: Supplementary file 4 [file 61993_Lisowska_DataSheet4.PDF]

Supplementary Table 3. FIGO stage

List of genes with significantly changed expression in the comparison of FIGO II *versus* FIGO III/IV stages of ovarian cancer; 714 probe sets with p<0.001 were selected in Welch test.

Affymetrix probe set ID	p-value	Gene symbol	Gene name
208239_at	4,03E-20	FOXE1	forkhead box E1 (thyroid transcription factor 2)
1561613_at	3,59E-19		CDNA clone IMAGE:3857861
204358_s_at	1,72E-18	FLRT2	fibronectin leucine rich transmembrane protein 2
1556088_at	8,69E-17	RIP	RPA interacting protein
238972_at	1,20E-15		Transcribed locus
211543_s_at	2,68E-15	GRK6	G protein-coupled receptor kinase 6
206746_at	1,26E-14	BFSP1	beaded filament structural protein 1, filensin
1553220_at	1,82E-14	ALS2CR13	amyotrophic lateral sclerosis 2 (juvenile) chromosome region, candidate 13
208904_s_at	4,19E-14	RPS28	ribosomal protein S28
213726_x_at	5,95E-14	TUBB2	tubulin, beta, 2
208253_at	7,20E-14	SIGLEC8	sialic acid binding Ig-like lectin 8
1560091_a_at	1,11E-13	PHF21B	PHD finger protein 21B
222481_at	1,33E-13	FXC1	fracture callus 1 homolog (rat)
215869_at	2,25E-13	ABCA1	ATP-binding cassette, sub-family A (ABC1), member 1
202328_s_at	2,85E-13	PKD1	polycystic kidney disease 1 (autosomal dominant)
227186_s_at	3,27E-13	MRPL41	mitochondrial ribosomal protein L41
1552631_a_at	3,33E-13	MAP3K6	mitogen-activated protein kinase kinase kinase 6
238310_at	5,07E-13		
203100_s_at	8,95E-13	CDYL	chromodomain protein, Y-like
203050_at	9,52E-13	TP53BP1	tumor protein p53 binding protein, 1
237290_at	1,34E-12		Transcribed locus
228241_at	1,36E-12	BCMP11	breast cancer membrane protein 11
1562373_at	1,57E-12	ITPR1	Inositol 1,4,5-triphosphate receptor, type 1
225257_at	1,97E-12	MGC20255	hypothetical protein MGC20255
203195_s_at	2,07E-12	NUP98	nucleoporin 98kDa
204163_at	2,50E-12	EMILIN1	elastin microfibril interfacer 1
225425_s_at	2,65E-12	MRPL41	mitochondrial ribosomal protein L41
235179_at	4,44E-12	ZNF641	zinc finger protein 641
208339_at	6,85E-12	XKRY ; XKRY2	X Kell blood group precursor-related, Y-linked ; X Kell blood group precursor-related, Y-linked 2
212762_s_at	9,91E-12	TCF7L2	transcription factor 7-like 2 (T-cell specific, HMG-box)
235471_at	1,72E-11	C10orf72	Chromosome 10 open reading frame 72
225891_at	3,32E-11	C9orf75	chromosome 9 open reading frame 75
1555743_s_at	4,60E-11		
219470_x_at	7,33E-11	CCNJ	cyclin J
221217_s_at	8,70E-11	A2BP1	ataxin 2-binding protein 1
1554306_at	9,09E-11	ITPKB	inositol 1,4,5-trisphosphate 3-kinase B
208874_x_at	9,70E-11	PPP2R4	protein phosphatase 2A, regulatory subunit B' (PR 53)
225486_at	9,75E-11	ARID2	AT rich interactive domain 2 (ARID, RFX-like)
236477_at	1,12E-10	FNBP3	Formin binding protein 3
203290_at	1,14E-10	HLA-DQA1	major histocompatibility complex, class II, DQ alpha 1 ; major histocompatibility complex, class II, DQ alpha 1
235150_at	1,17E-10	SESN3	Sestrin 3
1555090_x_at	1,26E-10	FLJ30294	hypothetical protein FLJ30294
209470_s_at	1,30E-10	GPM6A	glycoprotein M6A
1562303_at	1,41E-10	ZNF306	Zinc finger protein 306
201273_s_at	1,67E-10	SRP9	signal recognition particle 9kDa
203977_at	1,94E-10	TAZ	tafazzin (cardiomyopathy, dilated 3A (X-linked); endocardial fibroelastosis 2; Barth syndrome)
225357_s_at	2,09E-10	INO80	homolog of yeast INO80
235714_at	2,10E-10		
216119_s_at	2,17E-10	C20orf28	chromosome 20 open reading frame 28
233364_s_at	3,72E-10		Hypothetical gene supported by BX647608
218934_s_at	4,65E-10	HSPB7	heat shock 27kDa protein family, member 7 (cardiovascular)
225131_at	4,84E-10	ZRANB1	zinc finger, RAN-binding domain containing 1

201529_s_at	4,98E-10	RPA1	replication protein A1, 70kDa
1561383_at	5,00E-10	LOC284661	hypothetical protein LOC284661
244392_at	5,22E-10	USP3	Ubiquitin specific peptidase 3
239288_at	5,73E-10	TNIK	TRAF2 and NCK interacting kinase
240170_at	7,26E-10		Transcribed locus
234628_at	9,02E-10	RAB28	RAB28, member RAS oncogene family
1559108_at	9,61E-10	FLJ10979	Hypothetical protein FLJ10979
238244_at	1,00E-09		LOC441370
208385_at	1,19E-09	NR2E3	nuclear receptor subfamily 2, group E, member 3
215156_at	1,22E-09	REC14	WD repeat domain 61
1563458_at	1,29E-09	PARVA	parvin, alpha
233516_s_at	1,41E-09	SPAG17	sperm associated antigen 17
1553349_at	1,54E-09	ARID2	AT rich interactive domain 2 (ARID, RFX-like)
227053_at	1,63E-09	PACSIN1	protein kinase C and casein kinase substrate in neurons 1
233013_x_at	1,90E-09	RPL7L1 ; LOC401197	ribosomal protein L7-like 1 ; similar to RPL7L1 protein
234236_at	1,93E-09	FLJ20294	hypothetical protein FLJ20294
201561_s_at	1,98E-09	CLSTN1	calsyntenin 1
223270_at	2,14E-09	CTDSPL2	CTD (carboxy-terminal domain, RNA polymerase II, polypeptide A) small phosphatase like 2
223050_s_at	2,71E-09	FBXW5	F-box and WD-40 domain protein 5
1566094_at	2,93E-09	ARHGEF12	Rho guanine nucleotide exchange factor (GEF) 12
243380_at	3,10E-09	SLC4A5	Solute carrier family 4, sodium bicarbonate cotransporter, member 5
1557293_at	3,85E-09	LOC440993	hypothetical gene supported by AK128346
238088_at	4,02E-09		CDNA clone IMAGE:5259414
223313_s_at	4,13E-09	MAGED4	melanoma antigen family D, 4
206161_s_at	4,55E-09	SYT5	synaptotagmin V
210794_s_at	5,42E-09	MEG3	maternally expressed 3
222331_at	5,54E-09		
233072_at	6,25E-09	NTNG2	netrin G2
203675_at	6,96E-09	NUCB2	nucleobindin 2
1567861_at	6,99E-09	DNAH14	dynein, axonemal, heavy polypeptide 14
205914_s_at	9,24E-09	GRIN1	glutamate receptor, ionotropic, N-methyl D-aspartate 1
1569577_x_at	1,08E-08		Homo sapiens, clone IMAGE:3865586, mRNA
213059_at	1,19E-08	CREB3L1	cAMP responsive element binding protein 3-like 1
203772_at	1,24E-08	BLVRA	biliverdin reductase A
244623_at	2,07E-08		Transcribed locus
1558502_s_at	2,21E-08	DNM3	dynamin 3
240133_x_at	2,41E-08		Transcribed locus
223487_x_at	3,09E-08	GNB4	guanine nucleotide binding protein (G protein), beta polypeptide 4
243053_x_at	3,13E-08		Hypothetical LOC388738
230968_at	3,27E-08		Full-length cDNA clone CS0DF032YA11 of Fetal brain of Homo sapiens (human)
241189_at	3,33E-08		
237434_x_at	3,35E-08	LOC345222	hypothetical gene supported by BC043530
1567656_at	3,58E-08	OR2H1	Olfactory receptor, family 2, subfamily H, member 1
202426_s_at	3,72E-08	RXRA	retinoid X receptor, alpha
224013_s_at	3,92E-08	SOX7	SRY (sex determining region Y)-box 7
242915_at	3,99E-08	ZNF682	zinc finger protein 682
1556734_at	4,07E-08	LMO7	LIM domain 7
228163_at	4,55E-08	SIAT7D	ST6 (alpha-N-acetyl-neuraminyl-2,3-beta-galactosyl-1,3)-N-acetylgalactosaminide alpha-2,6-sialyltransferase 4
217530_at	4,73E-08	SLC34A1	solute carrier family 34 (sodium phosphate), member 1
206909_at	5,11E-08		
239465_at	5,20E-08	UQCRC2	Ubiquinol-cytochrome c reductase core protein II
224242_at	5,54E-08	GALP	galanin-like peptide precursor
207786_at	5,57E-08	CYP2R1	cytochrome P450, family 2, subfamily R, polypeptide 1
211794_at	5,79E-08	FYB	FYN binding protein (FYB-120/130)
228720_at	6,17E-08	SORCS2	sortilin-related VPS10 domain containing receptor 2
1569689_s_at	6,94E-08	GABRB3	gamma-aminobutyric acid (GABA) A receptor, beta 3
1569063_at	7,25E-08	KBTBD9	Kelch repeat and BTB (POZ) domain containing 9
1557861_at	9,38E-08		CDNA clone IMAGE:5302109

214619_at	1,00E-07	CRHR1	corticotropin releasing hormone receptor 1
207820_at	1,08E-07	ADH1A	alcohol dehydrogenase 1A (class I), alpha polypeptide
228183_s_at	1,08E-07	RIP	RPA interacting protein
211690_at	1,09E-07		
227939_s_at	1,16E-07	TRA2A	Transformer-2 alpha
242304_at	1,17E-07	WIBG	within bgcn homolog (Drosophila)
224481_s_at	1,29E-07	HECTD1	HECT domain containing 1 ; HECT domain containing 1
225865_x_at	1,35E-07	TH1L	TH1-like (Drosophila)
241591_at	1,44E-07		
212100_s_at	1,47E-07	POLDIP3	polymerase (DNA-directed), delta interacting protein 3
205694_at	1,54E-07	TYRP1	tyrosinase-related protein 1
233404_at	1,57E-07		CDNA FLJ13756 fis, clone PLACE3000365
1553882_at	1,60E-07		CDNA clone IMAGE:3547043
200871_s_at	1,64E-07	PSAP	prosaposin (variant Gaucher disease and variant metachromatic leukodystrophy)
1553991_s_at	1,99E-07	FLJ20674	hypothetical protein FLJ20674
230886_at	2,07E-07		Transcribed locus
214334_x_at	2,19E-07	DAZAP2 ; LOC401029	DAZ associated protein 2 ; similar to DAZ-associated protein 2 (Deleted in azoospermia-associated protein 2)
226068_at	2,21E-07	SYK	Spleen tyrosine kinase
243016_at	2,36E-07	TYMS	Thymidylate synthetase
235433_at	2,70E-07	CXorf33	chromosome X open reading frame 33
230554_at	2,80E-07	LOC123876	hypothetical protein LOC123876
231557_at	2,85E-07	DKFZp434L142	Hypothetical protein DKFZp434L142
244417_at	2,93E-07		Transcribed locus
1563154_at	2,96E-07	ADRA1B	Adrenergic, alpha-1B-, receptor
229312_s_at	3,10E-07	GKAP1	G kinase anchoring protein 1
227271_at	3,13E-07	FGF11	fibroblast growth factor 11
1569764_at	3,20E-07		
236455_at	3,32E-07		
235758_at	3,40E-07	PNMA6A	paraneoplastic antigen like 6A
1559622_at	3,51E-07		MRNA; cDNA DKFZp313O2039 (from clone DKFZp313O2039)
224579_at	3,52E-07	SLC38A1	Solute carrier family 38, member 1
200925_at	3,55E-07	COX6A1	cytochrome c oxidase subunit VIa polypeptide 1
205714_s_at	3,67E-07	ZMYND10	zinc finger, MYND-type containing 10
232218_at	3,78E-07		CDNA: FLJ22437 fis, clone HRC09230
65591_at	3,81E-07	WDR48	WD repeat domain 48
207977_s_at	4,29E-07	DPT	dermatopontin
203919_at	4,33E-07	TCEA2	transcription elongation factor A (SII), 2
212982_at	4,37E-07	ZDHHC17	zinc finger, DHHC-type containing 17
205465_x_at	4,43E-07	HS3ST1	heparan sulfate (glucosamine) 3-O-sulfotransferase 1
1555640_at	4,93E-07	DAOA	D-amino acid oxidase activator
237367_x_at	4,93E-07	CFLAR	CASP8 and FADD-like apoptosis regulator
220234_at	4,95E-07	CA8	carbonic anhydrase VIII
220605_s_at	5,00E-07	SIRT2	sirtuin (silent mating type information regulation 2 homolog) 2 (S. cerevisiae)
213656_s_at	5,02E-07	KNS2	kinesin 2
231996_at	5,45E-07	N4BP2	Nedd4 binding protein 2
216262_s_at	6,13E-07	TGIF2	TGFB-induced factor 2 (TALE family homeobox)
212124_at	6,65E-07	RAI17	retinoic acid induced 17
227712_at	6,87E-07	DJ122O8.2	hypothetical protein dJ122O8.2
1568807_a_at	7,21E-07	NDFIP2	Nedd4 family interacting protein 2
235130_at	7,45E-07	PANK2	Pantothenate kinase 2 (Hallervorden-Spatz syndrome)
201321_s_at	7,50E-07	SMARCC2	SWI/SNF related, matrix associated, actin dependent regulator of chromatin, subfamily c, member 2
1561152_a_at	7,54E-07	ZC3HC1	Zinc finger, C3HC-type containing 1
221220_s_at	7,65E-07	SCYL2	SCY1-like 2 (S. cerevisiae)
241701_at	8,07E-07	ARHGAP21	Rho GTPase activating protein 21
216601_at	8,58E-07	LOC90586	AOC3 pseudogene
234860_at	8,82E-07	TRAV20	T cell receptor alpha variable 20
229897_at	9,09E-07	FLJ31295	Zinc finger protein 641
214431_at	9,20E-07	GMPS	guanine monphosphate synthetase

239069_s_at	1,04E-06		Transcribed locus
235681_at	1,12E-06	HIST1H2BD	Histone 1, H2bd
207679_at	1,14E-06	PAX3	paired box gene 3 (Waardenburg syndrome 1)
228902_at	1,38E-06	NUP214	nucleoporin 214kDa
223823_at	1,43E-06	KCNMB2	potassium large conductance calcium-activated channel, subfamily M, beta member 2
225006_x_at	1,48E-06	TH1L	TH1-like (Drosophila)
201651_s_at	1,49E-06	PACSIN2	protein kinase C and casein kinase substrate in neurons 2
211495_x_at	1,60E-06	TNFSF13 ; TNFSF12-TNFSF13	tumor necrosis factor (ligand) superfamily, member 13 ; tumor necrosis factor (ligand) superfamily, member 12-member 13
241503_at	1,66E-06	MGC26690	Hypothetical protein MGC26690
229352_at	1,75E-06	SPESP1	sperm equatorial segment protein 1
229973_at	1,75E-06	C1orf173	chromosome 1 open reading frame 173
209269_s_at	1,80E-06	SYK	Spleen tyrosine kinase
237750_at	1,81E-06	LOC63929	Hypothetical protein LOC63929
203926_x_at	1,84E-06	ATP5D	ATP synthase, H+ transporting, mitochondrial F1 complex, delta subunit
201262_s_at	1,88E-06	BGN	biglycan
242393_x_at	1,90E-06	LOC399768	hypothetical gene supported by AK093334; AL833330; BC020871; BC032492
244639_at	1,94E-06	CGI-119	Transmembrane BAX inhibitor motif containing 4
234339_s_at	1,94E-06	GLTSCR2	glioma tumor suppressor candidate region gene 2
206032_at	2,00E-06	DSC3	desmocollin 3
209243_s_at	2,02E-06	PEG3	paternally expressed 3
217788_s_at	2,04E-06	GALNT2	UDP-N-acetyl-alpha-D-galactosamine:polypeptide N-acetylgalactosaminyltransferase 2 (GalNAc-T2)
200934_at	2,10E-06	DEK	DEK oncogene (DNA binding)
217077_s_at	2,15E-06	GPR51	G protein-coupled receptor 51
204061_at	2,35E-06	PRKX	protein kinase, X-linked
227243_s_at	2,44E-06	EBF3	early B-cell factor 3
206463_s_at	2,49E-06	DHRS2	dehydrogenase/reductase (SDR family) member 2
208757_at	2,55E-06	TMED9	transmembrane emp24 protein transport domain containing 9
244393_x_at	2,78E-06	AKR1CL2	Aldo-keto reductase family 1, member C-like 2
223609_at	2,82E-06	ROPN1L	ropporin 1-like
239150_at	2,85E-06	LOC132203	similar to hypothetical protein A430083B19
205825_at	2,92E-06	PCSK1	proprotein convertase subtilisin/kexin type 1
229482_at	3,02E-06	DDX51	DEAD (Asp-Glu-Ala-Asp) box polypeptide 51
222407_s_at	3,16E-06	ZFP106	zinc finger protein 106 homolog (mouse)
205769_at	3,36E-06	SLC27A2	solute carrier family 27 (fatty acid transporter), member 2
209469_at	3,47E-06	GPM6A	glycoprotein M6A
225001_at	3,66E-06	RAB3D	RAB3D, member RAS oncogene family
237281_at	3,80E-06	AKAP14	A kinase (PRKA) anchor protein 14
232645_at	3,89E-06	LOC153684	hypothetical protein LOC153684
238505_at	3,95E-06	ADPRH	ADP-ribosylarginine hydrolase
1568820_a_at	3,98E-06	C6orf65	chromosome 6 open reading frame 65
219240_s_at	4,14E-06	C10orf88	chromosome 10 open reading frame 88
227591_at	4,15E-06	SH3BP5	SH3-domain binding protein 5 (BTK-associated)
217787_s_at	4,15E-06	GALNT2	UDP-N-acetyl-alpha-D-galactosamine:polypeptide N-acetylgalactosaminyltransferase 2 (GalNAc-T2)
203074_at	4,24E-06	ANXA8	annexin A8
1554141_s_at	4,30E-06	WDR78	WD repeat domain 78
232441_at	4,52E-06	HRB2	HIV-1 rev binding protein 2
213695_at	4,58E-06	PON3	paraoxonase 3
1556689_a_at	4,58E-06	WNT4	wingless-type MMTV integration site family, member 4
204060_s_at	4,89E-06	PRKX ; PRKY	protein kinase, X-linked ; protein kinase, Y-linked
218591_s_at	5,05E-06	NOL10	nucleolar protein 10
234577_at	5,24E-06		MRNA; cDNA DKFZp434I1526 (from clone DKFZp434I1526)
1553169_at	5,38E-06	C20orf75	chromosome 20 open reading frame 75
208646_at	5,49E-06	RPS14	ribosomal protein S14
241615_x_at	5,74E-06	DAF	Decay accelerating factor for complement (CD55, Cromer blood group system)
217061_s_at	5,83E-06	ETV1	ets variant gene 1

242792_at	6,11E-06	NFIB	Nuclear factor I/B
223348_x_at	6,23E-06	MUM1	melanoma associated antigen (mutated) 1
229232_at	6,51E-06	FLJ36812	hypothetical protein FLJ36812
240908_at	6,54E-06		Similar to Microneme antigen
201368_at	6,64E-06	ZFP36L2	zinc finger protein 36, C3H type-like 2
1559316_at	6,67E-06		CDNA FLJ36043 fis, clone TESTI2017582
1569462_x_at	7,22E-06	KCNT1	potassium channel, subfamily T, member 1
216663_s_at	7,23E-06	ZMYND10	zinc finger, MYND-type containing 10
239825_at	7,34E-06	ATF6	Activating transcription factor 6
205528_s_at	7,41E-06	RUNX1T1	runt-related transcription factor 1; translocated to, 1 (cyclin D-related)
222310_at	7,44E-06	SFRS15	splicing factor, arginine/serine-rich 15
223257_at	7,45E-06	KIAA1333	KIAA1333
210792_x_at	7,59E-06	SIVA	CD27-binding (Siva) protein
237531_at	8,04E-06	C2orf3	Chromosome 2 open reading frame 3
207375_s_at	8,11E-06	IL15RA	interleukin 15 receptor, alpha
207935_s_at	8,11E-06	KRT13	keratin 13
219876_s_at	8,23E-06	DKFZp434M0331	hypothetical protein DKFZp434M0331
229668_at	8,52E-06	LOC90393	hypothetical protein LOC90393
234254_at	9,15E-06		
1558136_s_at	9,61E-06	TAF11	TAF11 RNA polymerase II, TATA box binding protein (TBP)-associated factor, 28kDa
237566_at	9,61E-06		Transcribed locus
207850_at	9,78E-06	CXCL3	chemokine (C-X-C motif) ligand 3
229572_at	9,83E-06		Transcribed locus, moderately similar to XP_508230.1 PREDICTED: zinc finger protein 195 [Pan troglodytes]
234655_at	9,86E-06		
215150_at	1,02E-05	YOD1	YOD1 OTU deubiquinating enzyme 1 homolog (yeast)
201492_s_at	1,03E-05	RPL41	ribosomal protein L41
202414_at	1,04E-05	ERCC5	excision repair cross-complementing rodent repair deficiency, complementation group 5 (xeroderma pigmentosum, complementation group G (Cockayne syndrome))
227592_at	1,06E-05	ALDH16A1	aldehyde dehydrogenase 16 family, member A1
219393_s_at	1,12E-05	AKT3	v-akt murine thymoma viral oncogene homolog 3 (protein kinase B, gamma)
204086_at	1,13E-05	PRAME	preferentially expressed antigen in melanoma
234257_at	1,15E-05	TENS1	Tensin 3
243900_at	1,17E-05	LOC401551	similar to hypothetical protein FLJ25955
224941_at	1,17E-05	PAPPA	pregnancy-associated plasma protein A, pappalysin 1
1556536_at	1,23E-05	FLJ39061	Hypothetical protein FLJ39061
235700_at	1,25E-05	CT45-2 ; CT45-3 ; CT45-4 ; CT45-5 ; CT45-6 ; CT45-1	cancer/testis antigen CT45-2 ; cancer/testis antigen CT45-3 ; cancer/testis antigen CT45-4 ; cancer/testis antigen CT45-5 ; cancer/testis antigen CT45-6 ; cancer/testis antigen CT45-1
201528_at	1,26E-05	RPA1	replication protein A1, 70kDa
1553171_x_at	1,31E-05	C20orf75	chromosome 20 open reading frame 75
243574_at	1,43E-05	HADHSC	L-3-hydroxyacyl-Coenzyme A dehydrogenase, short chain
1559096_x_at	1,44E-05	FBXO9	F-box protein 9
234248_at	1,47E-05		
241535_at	1,48E-05		Transcribed locus
215636_at	1,50E-05	RBAF600	retinoblastoma-associated factor 600
204033_at	1,51E-05	TRIP13	thyroid hormone receptor interactor 13
224953_at	1,54E-05	YIPF5	Yip1 domain family, member 5
218178_s_at	1,58E-05	CHMP1B	chromatin modifying protein 1B
222876_s_at	1,68E-05	CENTA2	centaurin, alpha 2
223002_s_at	1,80E-05	XRN2	5'-3' exoribonuclease 2
226070_at	1,83E-05	LOC286257	hypothetical protein LOC286257
201012_at	1,89E-05	ANXA1	annexin A1
237898_at	1,93E-05		Transcribed locus
1555907_at	1,96E-05	CENTG1	Centaurin, gamma 1
240747_at	2,02E-05		
1558692_at	2,06E-05	MGC13102	Hypothetical protein MGC13102
243041_s_at	2,08E-05	RBMS3	RNA binding motif, single stranded interacting protein
1560169_at	2,10E-05	C9orf42	Chromosome 9 open reading frame 42

202365_at	2,13E-05	MGC5139	hypothetical protein MGC5139
242558_at	2,14E-05	CTNNB1	Catenin (cadherin-associated protein), beta 1, 88kDa
224229_s_at	2,16E-05	AKT3	v-akt murine thymoma viral oncogene homolog 3 (protein kinase B, gamma)
206924_at	2,20E-05	IL11	interleukin 11
234798_x_at	2,35E-05	C20orf66	chromosome 20 open reading frame 66
239301_at	2,41E-05	RASA1	RAS p21 protein activator (GTPase activating protein) 1
231766_s_at	2,52E-05	COL12A1	collagen, type XII, alpha 1
1566677_at	2,53E-05	MMP2	Matrix metallopeptidase 2 (gelatinase A, 72kDa gelatinase, 72kDa type IV collagenase)
210854_x_at	2,53E-05	SLC6A8	solute carrier family 6 (neurotransmitter transporter, creatine), member 8
227191_at	2,60E-05	CDA08	T-cell immunomodulatory protein
209958_s_at	2,62E-05	PTHB1	parathyroid hormone-responsive B1
1562173_a_at	2,67E-05		Clone IMAGE:501887, mRNA sequence
202848_s_at	2,69E-05	GRK6	G protein-coupled receptor kinase 6
208748_s_at	2,73E-05	FLOT1	flotillin 1
206206_at	2,82E-05	CD180	CD180 antigen
235649_at	2,89E-05	ADAMTS8	ADAM metallopeptidase with thrombospondin type 1 motif, 8
235151_at	2,92E-05	LOC283357	hypothetical protein LOC283357
213085_s_at	2,95E-05	KIBRA	KIBRA protein
37549_g_at	3,13E-05	PTHB1	parathyroid hormone-responsive B1
223962_at	3,22E-05	NYD-SP14	NYD-SP14 protein
202278_s_at	3,22E-05	SPTLC1	serine palmitoyltransferase, long chain base subunit 1
208256_at	3,37E-05	EFNA2	ephrin-A2
1569022_a_at	3,43E-05	PIK3C2A	phosphoinositide-3-kinase, class 2, alpha polypeptide
228056_s_at	3,48E-05	NAPSB	napsin B aspartic peptidase pseudogene
211547_s_at	3,48E-05	PAFAH1B1	platelet-activating factor acetylhydrolase, isoform Ib, alpha subunit 45kDa
1567912_s_at	3,52E-05	CT45-2 ; CT45-3 ; CT45-4 ; CT45-5 ; CT45-6 ; CT45-1	cancer/testis antigen CT45-2 ; cancer/testis antigen CT45-3 ; cancer/testis antigen CT45-4 ; cancer/testis antigen CT45-5 ; cancer/testis antigen CT45-6 ; cancer/testis antigen CT45-1
204606_at	3,54E-05	CCL21	chemokine (C-C motif) ligand 21
202226_s_at	3,54E-05	CRK	v-crk sarcoma virus CT10 oncogene homolog (avian)
236342_at	3,65E-05	PLEKHG1	Pleckstrin homology domain containing, family G (with RhoGef domain) member 1
220884_at	3,68E-05		
234647_at	3,72E-05	CSEN	Calsenilin, presenilin binding protein, EF-hand transcription factor
214568_at	3,74E-05	TPSD1	tryptase delta 1
216333_x_at	3,76E-05	TNXB	tenascin XB
1559861_at	3,77E-05	FBP2	Fructose-1,6-bisphosphatase 2
217465_at	3,82E-05		
1553604_at	3,84E-05	ABCA13	ATP-binding cassette, sub-family A (ABC1), member 13
232523_at	3,85E-05	MEGF10	MEGF10 protein
1554372_at	3,89E-05		
219154_at	3,89E-05		Hypothetical LOC144404
217964_at	3,90E-05	TTC19	tetratricopeptide repeat domain 19
237714_at	3,98E-05		Transcribed locus
1558814_s_at	3,98E-05	TMED5	transmembrane emp24 protein transport domain containing 5
1558014_s_at	4,02E-05	MLSTD2	male sterility domain containing 2
230593_at	4,06E-05		Transcribed locus
237549_at	4,08E-05		CDNA FLJ42790 fis, clone BRAWH3007455
1552754_a_at	4,16E-05	IGSF4D	immunoglobulin superfamily, member 4D
242208_at	4,35E-05	ZNF37B	Zinc finger protein 37b (KOX 21)
209773_s_at	4,37E-05	RRM2	ribonucleotide reductase M2 polypeptide
242751_at	4,44E-05	PRDX6	Peroxiredoxin 6
231883_at	4,49E-05	FBXW8	F-box and WD-40 domain protein 8
1569761_x_at	4,57E-05		Similar to 27 kDa Golgi SNARE protein (Golgi SNAP receptor complex member 2) (Membrin)
240815_at	4,65E-05	SEMA3C	Sema domain, immunoglobulin domain (Ig), short basic domain, secreted, (semaphorin) 3C
223897_at	4,70E-05	LOC91661	hypothetical protein BC001610
237282_s_at	4,87E-05	AKAP14	A kinase (PRKA) anchor protein 14

1568924_a_at	4,92E-05	FLJ35834	hypothetical protein FLJ35834
239406_at	4,94E-05	ZNF193	Zinc finger protein 193
1560425_s_at	5,00E-05		CDNA FLJ37023 fis, clone BRACE2010828
1561484_at	5,03E-05		CDNA clone IMAGE:4819678
219796_s_at	5,15E-05	MUCDHL	mucin and cadherin-like
222856_at	5,25E-05	APLN	apelin, AGTRL1 ligand
213342_at	5,36E-05	YAP1	Yes-associated protein 1, 65kDa
235086_at	5,45E-05	THBS1	Thrombospondin 1
243087_at	5,51E-05	WDR63	WD repeat domain 63
227184_at	5,55E-05		Transcribed locus, weakly similar to NP_689672.2 hypothetical protein MGC45438 [Homo sapiens]
223532_at	5,67E-05	ANKRD39	ankyrin repeat domain 39
236653_at	5,90E-05		Transcribed locus
227776_at	6,00E-05		Transcribed locus
200645_at	6,06E-05	GABARAP	GABA(A) receptor-associated protein
224070_at	6,11E-05	tAKR	aldo-keto reductase, truncated
243110_x_at	6,39E-05	NPW	neuropeptide W
218909_at	6,53E-05	RPS6KC1	ribosomal protein S6 kinase, 52kDa, polypeptide 1
239638_at	6,58E-05		CDNA FLJ33227 fis, clone ASTRO2001088
1562406_at	6,72E-05	TRHDE	Thyrotropin-releasing hormone degrading enzyme
223267_at	6,73E-05	RG9MTD1	RNA (guanine-9-) methyltransferase domain containing 1
1568867_x_at	6,82E-05	KIAA0553	KIAA0553 protein
238368_at	6,86E-05	PVT1	Pvt1 oncogene homolog, MYC activator (mouse)
242186_x_at	6,87E-05	LPHN3	latrophilin 3
210814_at	7,07E-05	TRPC3	transient receptor potential cation channel, subfamily C, member 3
226063_at	7,27E-05	VAV2	vav 2 oncogene
206033_s_at	7,32E-05	DSC3	desmocollin 3
1007_s_at	7,44E-05	DDR1	discoidin domain receptor family, member 1
1553655_at	7,47E-05	FLJ37927	CDC20-like protein
211384_s_at	7,62E-05	CASR	calcium-sensing receptor (hypocalciuric hypercalcemia 1, severe neonatal hyperparathyroidism)
239161_at	7,70E-05	FDX1	ferredoxin 1
221057_at	7,74E-05	SPATA1	spermatogenesis associated 1
232140_at	7,74E-05		LOC441443
1569167_at	7,82E-05	COMMD10	COMM domain containing 10
208208_at	7,88E-05	MYH13	myosin, heavy polypeptide 13, skeletal muscle
203884_s_at	7,95E-05	RAB11FIP2	RAB11 family interacting protein 2 (class I)
204934_s_at	7,98E-05	HPN	hepsin (transmembrane protease, serine 1)
206111_at	8,10E-05	RNASE2	ribonuclease, RNase A family, 2 (liver, eosinophil-derived neurotoxin)
230071_at	8,13E-05	SEPT11	Septin 11
244723_at	8,20E-05		CDNA FLJ34659 fis, clone KIDNE2018863
1555895_at	8,29E-05	DNM2	dynamin 2
230068_s_at	8,40E-05	PEG3	Paternally expressed 3
242919_at	8,51E-05	LOC114977	hypothetical protein BC014148
1562371_s_at	8,61E-05	MGC26733	hypothetical protein MGC26733
1568999_at	8,62E-05	PSG4	pregnancy specific beta-1-glycoprotein 4
201051_at	8,64E-05	ANP32A	Acidic (leucine-rich) nuclear phosphoprotein 32 family, member A
1569344_a_at	8,83E-05		Homo sapiens, clone IMAGE:4044872, mRNA
244811_at	8,88E-05	IRAK1BP1	Interleukin-1 receptor-associated kinase 1 binding protein 1
208399_s_at	8,90E-05	EDN3	endothelin 3
1564402_at	8,93E-05	LOC146795	hypothetical protein LOC146795
217598_at	9,33E-05	CINP	Cyclin-dependent kinase 2-interacting protein
237690_at	9,48E-05	GPR115	G protein-coupled receptor 115
233591_at	9,76E-05		CDNA: FLJ23098 fis, clone LNG07440
208735_s_at	9,81E-05	CTDSP2	CTD (carboxy-terminal domain, RNA polymerase II, polypeptide A) small phosphatase 2
239432_at	9,94E-05		MRNA; cDNA DKFZp781K21161 (from clone DKFZp781K21161)
210831_s_at	9,94E-05	PTGER3	prostaglandin E receptor 3 (subtype EP3)
244014_x_at	9,96E-05	LOC137392	Family with sequence similarity 92, member A1
218865_at	0,0001	MOSC1	MOCO sulphurase C-terminal domain containing 1
1557693_at	1,00E-04		CDNA clone IMAGE:5303580
219454_at	0,000103	EGFL6	EGF-like-domain, multiple 6

229124_at	0,000104	PROK1	prokineticin 1
227468_at	0,000104	CPT1C	carnitine palmitoyltransferase 1C
231584_s_at	0,000105	BCAS4	Breast carcinoma amplified sequence 4
201330_at	0,000106	RARS	arginyl-tRNA synthetase
1561437_at	0,000111		CDNA clone IMAGE:5294936
204234_s_at	0,000115	ZNF195	zinc finger protein 195
239886_at	0,000118	RNF130	Ring finger protein 130
205394_at	0,000122	CHEK1	CHK1 checkpoint homolog (S. pombe)
64474_g_at	0,000123	DGCR8	DiGeorge syndrome critical region gene 8
225248_at	0,000125	SPPL2B	signal peptide peptidase-like 2B
225703_at	0,000126	KIAA1545	KIAA1545 protein
1565132_at	0,00013	RBMY3AP	RNA binding motif protein, Y-linked, family 3, member A pseudogene
223132_s_at	0,000131	TRIM8	tripartite motif-containing 8
209183_s_at	0,000131	C10orf10	chromosome 10 open reading frame 10
238551_at	0,000132	FUT11	fucosyltransferase 11 (alpha (1,3) fucosyltransferase)
228399_at	0,000134	OSR1	odd-skipped related 1 (Drosophila)
236502_at	0,000134		
229955_at	0,000135	FBXO3	F-box protein 3
219552_at	0,000135	C9orf13	Sushi, von Willebrand factor type A, EGF and pentraxin domain containing 1
243163_at	0,000136		Transcribed locus
205184_at	0,000139	GNG4	guanine nucleotide binding protein (G protein), gamma 4
214543_x_at	0,00014	QKI	quaking homolog, KH domain RNA binding (mouse)
233511_at	0,000142		Hypothetical gene supported by AK128010
212512_s_at	0,000143	CARM1	coactivator-associated arginine methyltransferase 1
203959_s_at	0,000143	ZBTB40	zinc finger and BTB domain containing 40
1555411_a_at	0,000144	CCNL1	cyclin L1
209025_s_at	0,000148	SYNCRIP	synaptotagmin binding, cytoplasmic RNA interacting protein
221916_at	0,000151	NEFL	Neurofilament, light polypeptide 68kDa
243819_at	0,000157	GNG2	Guanine nucleotide binding protein (G protein), gamma 2
238845_at	0,000159	SLC30A4	solute carrier family 30 (zinc transporter), member 4
240394_at	0,00016		Hypothetical gene supported by AK091668; BC043585; BX647564
220095_at	0,000161	C9orf39	chromosome 9 open reading frame 39
233365_at	0,000161	PRKG1	Protein kinase, cGMP-dependent, type I
244238_at	0,000164		Transcribed locus
242893_at	0,000166		
1564315_at	0,000166	C8orf49	chromosome 8 open reading frame 49
1568974_at	0,000168		CDNA clone IMAGE:4100365
224427_s_at	0,000169	PAPOLG	poly(A) polymerase gamma ; poly(A) polymerase gamma
213830_at	0,000169	TRD@	T cell receptor delta locus
210432_s_at	0,00017	SCN3A	sodium channel, voltage-gated, type III, alpha
201417_at	0,000181	SOX4	SRY (sex determining region Y)-box 4
224332_s_at	0,000187	MRPL43	mitochondrial ribosomal protein L43 ; mitochondrial ribosomal protein L43
202175_at	0,000187	CHPF	chondroitin polymerizing factor
202900_s_at	0,000188	NUP88	nucleoporin 88kDa
212944_at	0,000189	MRPS6	Mitochondrial ribosomal protein S6
227720_at	0,000193	ANKRD13B	ankyrin repeat domain 13B
226712_at	0,000195		CDNA: FLJ22100 fis, clone HEP17127
240752_at	0,000195	PCGF2	Polycomb group ring finger 2
204700_x_at	0,000197	C1orf107	chromosome 1 open reading frame 107
1559748_at	0,000201	ADAMTSL3	ADAMTS-like 3
225435_at	0,000203		CDNA: FLJ22100 fis, clone HEP17127
210645_s_at	0,000205	TTC3	tetratricopeptide repeat domain 3
209989_at	0,000206	ZNF268	zinc finger protein 268
209653_at	0,000209	KPNA4	karyopherin alpha 4 (importin alpha 3)
1552666_a_at	0,000214	LRRC7	leucine rich repeat containing 7
228238_at	0,000217	RNU47 ; GAS5	RNA, U47 small nuclear ; growth arrest-specific 5
208609_s_at	0,000219	TNXA ; TNXB	tenascin XA pseudogene ; tenascin XA pseudogene ; tenascin XB ; tenascin XB
240113_at	0,00022	SASH1	SAM and SH3 domain containing 1
1555971_s_at	0,000222	FBXO28	F-box protein 28

207750_at	0,000231	EPS15L2	epidermal growth factor receptor pathway substrate 15-like 2
204722_at	0,000232	SCN3B	sodium channel, voltage-gated, type III, beta
219460_s_at	0,000233	FLJ20507	hypothetical protein FLJ20507
235976_at	0,000233	SLITRK6	SLIT and NTRK-like family, member 6
223438_s_at	0,000234	PPARA	peroxisome proliferative activated receptor, alpha
216810_at	0,000235	KRTAP4-7	keratin associated protein 4-7
238984_at	0,000238		
243483_at	0,000239	TRPM8	transient receptor potential cation channel, subfamily M, member 8
205662_at	0,000243	EPPB9	B9 protein
1553888_at	0,000247	LDHAL6A	lactate dehydrogenase A-like 6A
234803_at	0,000249	CSTL1	cystatin-like 1
207307_at	0,000256	HTR2C	5-hydroxytryptamine (serotonin) receptor 2C
214433_s_at	0,000257	SELENBP1	selenium binding protein 1 ; selenium binding protein 1
201151_s_at	0,000264	MBNL1	muscleblind-like (Drosophila)
204647_at	0,000265	HOMER3	homer homolog 3 (Drosophila)
226994_at	0,000267	DNAJA2	DnaJ (Hsp40) homolog, subfamily A, member 2
32723_at	0,000273	CSTF1	cleavage stimulation factor, 3' pre-RNA, subunit 1, 50kDa
214111_at	0,000273	OPCML	opioid binding protein/cell adhesion molecule-like
243279_at	0,000274		Transcribed locus
211006_s_at	0,000275	KCNB1	potassium voltage-gated channel, Shab-related subfamily, member 1
236517_at	0,000276	MEGF10	MEGF10 protein
220187_at	0,000278	STEAP4	STEAP family member 4
236534_at	0,00028	BNIPL	BCL2/adenovirus E1B 19kD interacting protein like
221885_at	0,000283	DENND2A	DENN/MADD domain containing 2A
223924_at	0,000283	DKFZP434H0115	hypothetical protein DKFZp434H0115
231341_at	0,000286	SLC35D3	solute carrier family 35, member D3
201192_s_at	0,000287	PITPNA	phosphatidylinositol transfer protein, alpha
243173_at	0,000287	CABP7	calcium binding protein 7
219359_at	0,000298	FLJ22635	hypothetical protein FLJ22635
213843_x_at	0,000302	SLC6A8	solute carrier family 6 (neurotransmitter transporter, creatine), member 8
234205_at	0,000306	MAML2	Mastermind-like 2 (Drosophila)
1566324_a_at	0,000307	MAF	v-maf musculoaponeurotic fibrosarcoma oncogene homolog (avian)
229076_s_at	0,000307	TOMM22	Translocase of outer mitochondrial membrane 22 homolog (yeast)
203866_at	0,000307	NLE1	notchless homolog 1 (Drosophila)
234471_s_at	0,000308	NCOA5	nuclear receptor coactivator 5
203910_at	0,000312	ARHGAP29	Rho GTPase activating protein 29
242600_at	0,000319	FRMD3	FERM domain containing 3
242507_at	0,000319	KIAA0794	KIAA0794 protein
1556793_a_at	0,000322	C20orf128	chromosome 20 open reading frame 128
1553542_at	0,000327	KENAE	Kenae
1566694_at	0,000328		CDNA: FLJ20947 fis, clone ADSE01841
233217_at	0,000329	TM4SF16	Tetraspanin 16
230843_at	0,000333	FLJ10579	Family with sequence similarity 82, member C
242071_x_at	0,000334	ITGA8	integrin, alpha 8
1563466_at	0,000334	MYLK	Myosin, light polypeptide kinase
209195_s_at	0,000337	ADCY6	adenylate cyclase 6
210375_at	0,000338	PTGER3	prostaglandin E receptor 3 (subtype EP3)
220504_at	0,000339	KERA	keratocan
212000_at	0,00034	SFRS14	splicing factor, arginine/serine-rich 14
243426_at	0,000342	LOC339290	hypothetical protein LOC339290
212454_x_at	0,000343	HNRPDL	Heterogeneous nuclear ribonucleoprotein D-like
230665_at	0,000344		CDNA FLJ40823 fis, clone TRACH2011093
217390_x_at	0,000344		
210170_at	0,000351	PDLIM3	PDZ and LIM domain 3
204690_at	0,000352	STX8	syntaxin 8
219469_at	0,000354	DNCH2	dynein, cytoplasmic, heavy polypeptide 2
215644_at	0,000357	ZNF518	zinc finger protein 518
225987_at	0,000359	STEAP4	STEAP family member 4
203768_s_at	0,00036	STS	steroid sulfatase (microsomal), arylsulfatase C, isozyme S
232931_at	0,000362	ASCC3L1	Activating signal cointegrator 1 complex subunit 3-like 1

1556417_a_at	0,000369	FALZ	Fetal Alzheimer antigen
209215_at	0,000369	TETRAN	tetracycline transporter-like protein
208073_x_at	0,00037	TTC3	tetratricopeptide repeat domain 3
227653_at	0,000378	TRMT5	TRM5 tRNA methyltransferase 5 homolog (S. cerevisiae)
231231_at	0,000379		
210319_x_at	0,000384	MSX2	msh homeo box homolog 2 (Drosophila)
1562701_at	0,000384		CDNA FLJ37143 fis, clone BRACE2024222
214023_x_at	0,000385	TUBB-PARALOG	tubulin, beta polypeptide paralog
38340_at	0,000385	HIP1R	huntingtin interacting protein-1-related
210112_at	0,000398	HPS1	Hermansky-Pudlak syndrome 1
225261_x_at	0,000401	TH1L	TH1-like (Drosophila)
1557699_x_at	0,000402		Homo sapiens, clone IMAGE:4043992, mRNA
214789_x_at	0,000404	SRP46	Splicing factor, arginine/serine-rich, 46kD
206093_x_at	0,000404	TNXB	tenascin XB
208538_at	0,000408	ANP32C	acidic (leucine-rich) nuclear phosphoprotein 32 family, member C
236750_at	0,000415	NRXN3	Neurexin 3
201463_s_at	0,000423	TALDO1	transaldolase 1
229356_x_at	0,000425	INO80	homolog of yeast INO80
219444_at	0,000429	BCORL1	BCL6 co-repressor-like 1
223760_s_at	0,000429		
229091_s_at	0,000434	CCNJ	cyclin J
225320_at	0,000434	C10orf42	chromosome 10 open reading frame 42
238058_at	0,000438	LOC150381	hypothetical protein LOC150381
216035_x_at	0,00044	TCF7L2	transcription factor 7-like 2 (T-cell specific, HMG-box)
226109_at	0,000442	C21orf91	chromosome 21 open reading frame 91
221261_x_at	0,000443	MAGED4	melanoma antigen family D, 4 ; melanoma antigen family D, 4
220988_s_at	0,000451	C1QTNF3	C1q and tumor necrosis factor related protein 3 ; C1q and tumor necrosis factor related protein 3
232166_at	0,000455	KIAA1377	KIAA1377 protein
1569041_at	0,000457	JMJD1C	Hypothetical protein MGC14425
207899_at	0,000457	GIP	gastric inhibitory polypeptide
204259_at	0,000458	MMP7	matrix metallopeptidase 7 (matrilysin, uterine)
218683_at	0,000465	PTBP2	polypyrimidine tract binding protein 2
207928_s_at	0,000469	GLRA3	glycine receptor, alpha 3
230342_at	0,00047	FOXK1	forkhead box K1
1562812_at	0,00047	C10orf44	Chromosome 10 open reading frame 44
200043_at	0,000471	ERH	enhancer of rudimentary homolog (Drosophila) ; enhancer of rudimentary homolog (Drosophila)
227094_at	0,000475	DHTKD1	dehydrogenase E1 and transketolase domain containing 1
243896_at	0,000486	C10orf79	chromosome 10 open reading frame 79
1554518_at	0,000489	FLJ13273	hypothetical protein FLJ13273
231346_s_at	0,00049		
1564472_at	0,000495	LOC441306	hypothetical gene supported by AK097459
210303_at	0,000499	MAB21L2	mab-21-like 2 (C. elegans)
221858_at	0,000499	TBC1D12	TBC1 domain family, member 12
208806_at	0,0005	CHD3	chromodomain helicase DNA binding protein 3
202222_s_at	0,0005	DES	desmin
207853_s_at	0,00051	SNCB	synuclein, beta
209066_x_at	0,000513	UQCRB	ubiquinol-cytochrome c reductase binding protein
209896_s_at	0,000521	PTPN11	protein tyrosine phosphatase, non-receptor type 11 (Noonan syndrome 1)
220651_s_at	0,000521	MCM10	MCM10 minichromosome maintenance deficient 10 (S. cerevisiae)
215608_at	0,000531		
216740_at	0,000551	TRERF1	Transcriptional regulating factor 1
239731_at	0,000554		Transcribed locus
223905_at	0,000563	C16orf50	chromosome 16 open reading frame 50
237020_at	0,000564	MGC39581	hypothetical protein MGC39581
242094_at	0,000566		Full length insert cDNA clone YR40C10
244760_at	0,000567		
236194_at	0,00057		Transcribed locus
203932_at	0,000572	HLA-DMB	major histocompatibility complex, class II, DM beta ; major histocompatibility complex, class II, DM beta

220721_at	0,000574	ZNF614	zinc finger protein 614
238720_at	0,000574		
239041_at	0,000575	HIST1H2BN	Histone 1, H2bn
234348_at	0,000577		
1553989_a_at	0,000579	ATP6V1C2	ATPase, H+ transporting, lysosomal 42kDa, V1 subunit C isoform 2
244057_s_at	0,000588	C10orf72	chromosome 10 open reading frame 72
219336_s_at	0,000597	ASCC1	activating signal cointegrator 1 complex subunit 1
241545_x_at	0,0006	RBBP6	Retinoblastoma binding protein 6
240684_at	0,000601	SIPA1L3	Signal-induced proliferation-associated 1 like 3
1553446_at	0,000602	FLJ37396	hypothetical protein FLJ37396
222588_s_at	0,000613	FLJ10726	hypothetical protein FLJ10726
215325_x_at	0,000621	C19orf26	chromosome 19 open reading frame 26
227065_at	0,000623	TRIAD3	TRIAD3 protein
238260_at	0,000624	RAPGEF2	Rap guanine nucleotide exchange factor (GEF) 2
204524_at	0,000624	PDPK1	3-phosphoinositide dependent protein kinase-1
223364_s_at	0,000624	DHX37	DEAH (Asp-Glu-Ala-His) box polypeptide 37
226692_at	0,000627	SERF2	Small EDRK-rich factor 2
218767_at	0,000637	REXO4	REX4, RNA exonuclease 4 homolog (S. cerevisiae)
216903_s_at	0,000638	CBARA1	calcium binding atopy-related autoantigen 1
201145_at	0,000645	HAX1	HCLS1 associated protein X-1
1568606_at	0,000647	FLJ46266	FLJ46266 protein
211272_s_at	0,00065	DGKA	diacylglycerol kinase, alpha 80kDa
225389_at	0,000653	BTBD6	BTB (POZ) domain containing 6
227446_s_at	0,000654	C14orf167	chromosome 14 open reading frame 167
226871_s_at	0,000665	ATG4D	ATG4 autophagy related 4 homolog D (S. cerevisiae)
1556656_at	0,000671	FMNL2	Formin-like 2
205735_s_at	0,000675	AFF3	AF4/FMR2 family, member 3
225568_at	0,000683	MGC14141	hypothetical protein MGC14141
242424_at	0,000684	LETMD1	LETM1 domain containing 1
223089_at	0,000694	VEZATIN	transmembrane protein vezatin
220392_at	0,000698	EBF2	early B-cell factor 2
226764_at	0,000704	LOC152485	hypothetical protein LOC152485
242814_at	0,00071	SERPINB9	serpin peptidase inhibitor, clade B (ovalbumin), member 9
218685_s_at	0,000711	SMUG1	single-strand selective monofunctional uracil DNA glycosylase
224185_at	0,00073	FLJ10385	WD repeat domain 79
216105_x_at	0,000733	PPP2R4	protein phosphatase 2A, regulatory subunit B' (PR 53)
206432_at	0,000738	HAS2	hyaluronan synthase 2
224905_at	0,000741	WDR26	WD repeat domain 26
221241_s_at	0,000751	BCL2L14	BCL2-like 14 (apoptosis facilitator) ; BCL2-like 14 (apoptosis facilitator)
1565544_at	0,000759	RNF141	ring finger protein 141
233878_s_at	0,000762	XRN2	5'-3' exoribonuclease 2
212054_x_at	0,000763	KIAA0676	KIAA0676 protein
219873_at	0,000765	COLEC11	collectin sub-family member 11
206773_at	0,000776	LY6H	lymphocyte antigen 6 complex, locus H
1561880_a_at	0,00078	SIGLECP16	sialic acid binding Ig-like lectin, pseudogene 16
242592_at	0,000783	DKFZp762F0713	hypothetical protein DKFZp762F0713
1565917_at	0,000793		MRNA from chromosome 5q21-22, clone:LI26
205812_s_at	0,000806	TMED9	transmembrane emp24 protein transport domain containing 9
236709_at	0,000833	LOC400411	hypothetical LOC400411
243509_at	0,000835	BTG1	B-cell translocation gene 1, anti-proliferative
214167_s_at	0,000842	RPLP0 ; RPLP0-like	ribosomal protein, large, P0 ; similar to ribosomal protein P0
1559477_s_at	0,000843	MEIS1	Meis1, myeloid ecotropic viral integration site 1 homolog (mouse)
33323_r_at	0,000844	SFN	stratifin
227208_at	0,000856	DLNB14	similar to DLNB14
231800_s_at	0,000862	DMRT3	doublesex and mab-3 related transcription factor 3
223218_s_at	0,000865	NFKBIZ	nuclear factor of kappa light polypeptide gene enhancer in B-cells inhibitor, zeta
204193_at	0,000868	CHKB	choline kinase beta
221323_at	0,00087	ULBP1	UL16 binding protein 1
239477_at	0,000872	EFHB	EF-hand domain family, member B

213167_s_at	0,000875	MRPS6	Mitochondrial ribosomal protein S6
1554714_at	0,000877	FLJ11017	Hypothetical protein FLJ11017
232750_at	0,000877		CDNA FLJ13750 fis, clone PLACE3000331
211869_at	0,000883		
244212_at	0,000887	CCNC	Cyclin C
225123_at	0,000888	SESN3	Sestrin 3
233104_at	0,000894	C20orf119	Similar to embryonic poly(A) binding protein
225896_at	0,000895		CDNA: FLJ22382 fis, clone HRC07514
241377_s_at	0,00091	ZZANK1	Mindbomb homolog 2 (Drosophila)
229678_at	0,000916		MRNA; cDNA DKFZp762K056 (from clone DKFZp762K056)
221870_at	0,000916	EHD2	EH-domain containing 2
222539_at	0,000941	CLN6	ceroid-lipofuscinosis, neuronal 6, late infantile, variant
220315_at	0,000949	PARP11	poly (ADP-ribose) polymerase family, member 11
238237_at	0,000962		Transcribed locus, weakly similar to NP_064698.1 a disintegrin and metalloprotease domain 3 (cyritestin) [Rattus norvegicus]
214043_at	0,000962	PTPRD	Protein tyrosine phosphatase, receptor type, D
233071_at	0,000966	RSHL3	radial spokehead-like 3
223346_at	0,000967	VPS18	vacuolar protein sorting protein 18
222428_s_at	0,000975	LARS	leucyl-tRNA synthetase
239216_at	0,00098	TEKT1	tektin 1
217644_s_at	0,000984	SOS2	son of sevenless homolog 2 (Drosophila)
212561_at	0,000991	RAB6IP1	RAB6 interacting protein 1
221965_at	0,000997	MPHOSPH9	M-phase phosphoprotein 9
220153_at	0,000998	ENTPD7	ectonucleoside triphosphate diphosphohydrolase 7
