# Supplementary material for: Gene Expression Analysis in Ovarian Cancer – Faults and Hints from DNA Microarray Study
Source: Front Oncol. 2014 Jan 28;4:6. doi: 10.3389/fonc.2014.00006 (PMC3904181; doi:10.3389/fonc.2014.00006)
Supplement: Supplementary file 5 [file 61993_Lisowska_DataSheet5.PDF]

FIGO stage

1. Hierarchical clustering: Dendrogram shows the relations between ovarian cancer samples of different FIGO stages. Heat map shows relative intensity of genes expression. FIGO II samples are clustered together and show distinct gene expression pattern. FIGO III and FIGO IV samples are mixed together and do not show distinct gene expression pattern.

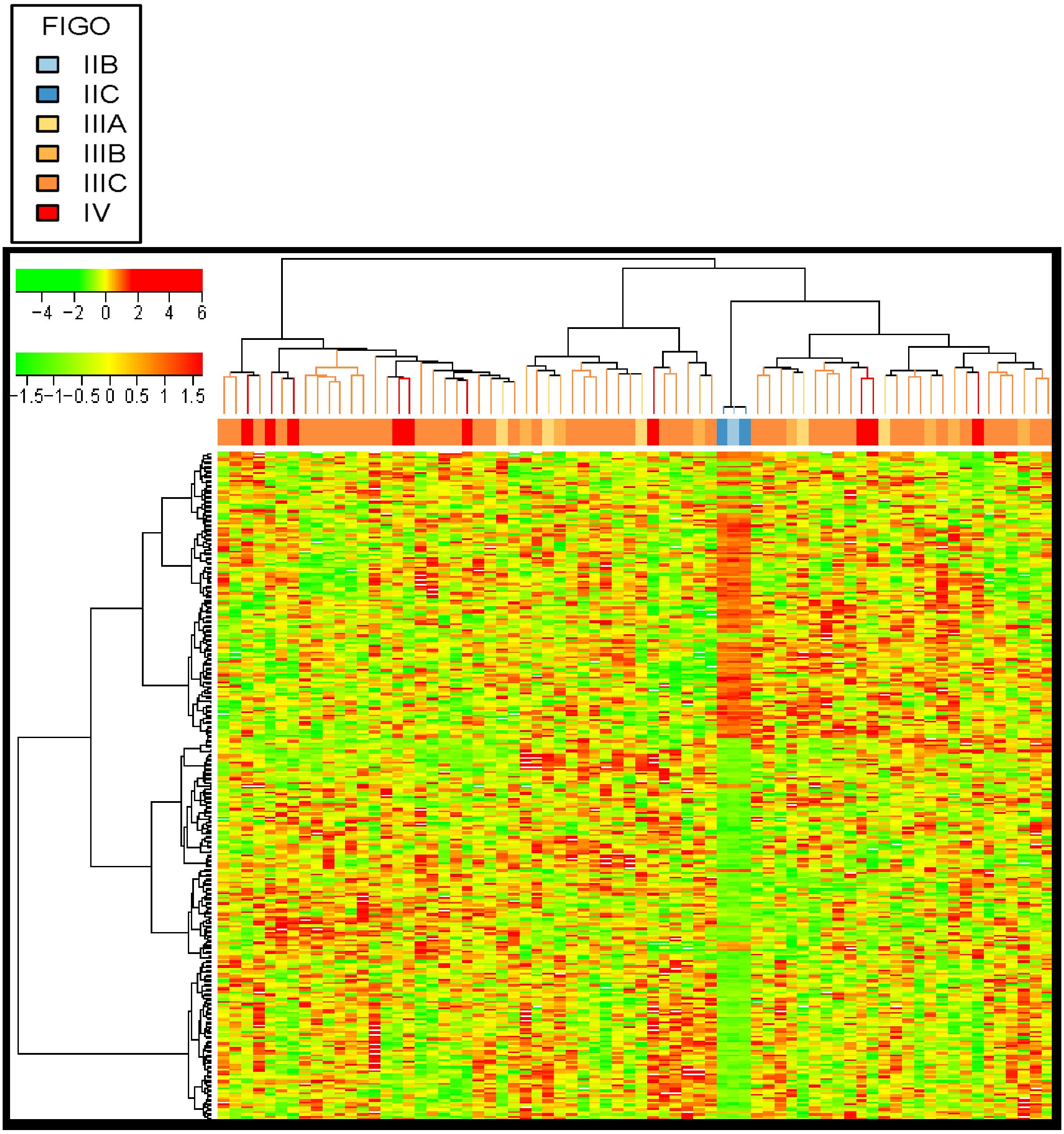

2. Gene Ontology analysis was performed using annotated genes selected in Welch ($p<0.001$) for the difference between FIGO II *versus* FIGO III/IV stages of ovarian cancer.

Gene Ontology class	p-value
GO:45830: positive regulation of isotype switching	0.00322
GO:48298: positive regulation of isotype switching to IgA isotypes	0.00322
GO:48296: regulation of isotype switching to IgA isotypes	0.00322
GO:48290: isotype switching to IgA isotypes	0.00322
GO:7159: leukocyte adhesion	0.0047
GO:50871: positive regulation of B cell activation	0.00543

GO:45444: fat cell differentiation	0.00635
GO:45191: regulation of isotype switching	0.00815
GO:9405: pathogenesis	0.00829
GO:7218: neuropeptide signaling pathway	0.00841
GO:51606: detection of stimulus	0.00932
GO:9582: detection of abiotic stimulus	0.00934
GO:45190: isotype switching	0.0097
GO:1504: neurotransmitter uptake	0.0097

3. Signaling pathways (according to Biocarta repository) with significantly changed gene expression between FIGO II *versus* FIGO III/IV stages of ovarian cancer. Least square (LS) and Kolomogorov-Smirnoff (KS) tests were used for analysis of annotated genes selected in Welch (p<0.001) for the difference between FIGO II *versus* FIGO III/IV stages.

	**Signaling pathways (Biocarta)**	**Signaling pathway name**	**p-value LS**	**p-value KS**
1	h_AcetaminophenPathway	Mechanism of Acetaminophen Activity and Toxicity	1e-05	1e-05
2	h_eicosanoidPathway	Eicosanoid Metabolism	1e-05	0.047444
3	h_sppaPathway	Aspirin Blocks Signaling Pathway Involved in Platelet Activation	0.0003494	0.0246272
4	h_bArrestinPathway	ß-arrestins in GPCR Desensitization	0.0019733	0.0048259
5	h_tnfr1Pathway	TNFR1 Signaling Pathway	0.0036629	0.095826
6	h_prionPathway	Prion Pathway	0.0044454	0.0157053
7	h_deathPathway	Induction of apoptosis through DR3 and DR4/5 Death Receptors	0.0045902	0.1032272
8	h_pparPathway	Basic mechanism of action of PPARa, PPARb(d) and PPARg and effects on gene expression	0.0051896	0.0003989
9	h_plcdPathway	Phospholipase C d1 in phospholipid associated cell signaling	0.0109403	9.18e-05
10	h_pkcPathway	Activation of PKC through G protein coupled receptor	0.0140429	0.0004615
11	h_tollPathway	Toll-Like Receptor Pathway	0.0163908	0.0021563
12	h_rnaPathway	Double Stranded RNA Induced Gene Expression	0.0359068	0.0025545
13	h_iresPathway	Internal Ribosome entry pathway	0.03749	0.0027449
14	h_myosinPathway	PKC-catalyzed phosphorylation of inhibitory phosphoprotein of myosin phosphatase	0.0427414	0.0030922
15	h_pitx2Pathway	Multi-step Regulation of Transcription by Pitx2	0.0944048	0.0044654
16	h_alkPathway	ALK in cardiac myocytes	0.1153683	0.0028702

4. Signaling pathways (according to Biocarta repository) with significantly changed gene expression between FIGO II *versus* FIGO III/IV stages of ovarian cancer.
Least square (LS) and Kolomogorov-Smirnoff (KS) tests were used for analysis of annotated genes selected in Welch test (p<0.001) for the difference between FIGO II *versus* FIGO III/IV stages.

	**Signaling pathways (Biocarta)**	**Signaling pathway name**	**p-value**
1	h_AcetaminophenPathway	Mechanism of Acetaminophen Activity and Toxicity	0.0006335
2	h_bArrestinPathway	ß-arrestins in GPCR Desensitization	0.0021596
3	h_sppaPathway	Aspirin Blocks Signaling Pathway Involved in Platelet Activation	0.0044041
4	h_akap13Pathway	Rho-Selective Guanine Exchange Factor AKAP13 Mediates Stress Fiber Formation	0.0048097
