# Supplementary material for: Gene Expression Analysis in Ovarian Cancer – Faults and Hints from DNA Microarray Study
Source: Front Oncol. 2014 Jan 28;4:6. doi: 10.3389/fonc.2014.00006 (PMC3904181; doi:10.3389/fonc.2014.00006)
Supplement: Supplementary file 6 [file 61993_Lisowska_DataSheet6.PDF]

Supplementary table 4. Tumor grade

List of genes with significantly changed expression in the comparison of grade 2 *versus* grade 3 + grade 4 tumors; 411 probe sets with p<0.001 were selected in Welch test.

Affymetrix probe set ID	p-value	Gene symbol	Gene name
239999_at	5,29E-10	C21orf34	Chromosome 21 open reading frame 34
230865_at	8,92E-09	LIX1	Lix1 homolog (mouse)
1557754_at	1,72E-08	LOC401068	hypothetical gene supported by BC028186
233753_at	1,02E-07	SFRS15	splicing factor, arginine/serine-rich 15
218736_s_at	1,05E-07	PALMD	palmdelphin
1562348_at	1,80E-07	LOC400680	hypothetical gene supported by AK097381; BC040866
215692_s_at	2,50E-07	MPPED2	metallophosphoesterase domain containing 2
239602_at	3,19E-07	C21orf87	Chromosome 21 open reading frame 87
1556444_a_at	4,32E-07		CDNA FLJ34367 fis, clone FEBRA2016621
226448_at	4,36E-07	FAM89A	family with sequence similarity 89, member A
226488_at	4,41E-07	RCCD1	RCC1 domain containing 1
218411_s_at	4,88E-07	MBIP	MAP3K12 binding inhibitory protein 1
229381_at	7,99E-07	C1orf64	chromosome 1 open reading frame 64
231470_at	8,00E-07		Transcribed locus
1562505_at	8,05E-07	STK35	Serine/threonine kinase 35
230465_at	1,67E-06	HS2ST1	heparan sulfate 2-O-sulfotransferase 1
207109_at	1,86E-06	POU2F3	POU domain, class 2, transcription factor 3
226621_at	2,43E-06	OSMR	Oncostatin M receptor
229349_at	2,46E-06	LIN28B	lin-28 homolog B (C. elegans)
229134_at	2,68E-06		Transcribed locus
206012_at	3,24E-06	LEFTY2	left-right determination factor 2
226926_at	3,57E-06	ZD52F10	dermokine
235441_at	3,72E-06	ADCY3	Adenylate cyclase 3
230720_at	4,28E-06	RNF182	ring finger protein 182
205934_at	5,93E-06	PLCL1	phospholipase C-like 1
1556827_at	6,58E-06	LOC339929	hypothetical protein LOC339929
238139_at	6,77E-06		Homo sapiens, clone IMAGE:4523945, mRNA
239703_at	7,35E-06		Transcribed locus
219251_s_at	7,79E-06	WDR60	WD repeat domain 60
232635_at	8,30E-06	C14orf145	chromosome 14 open reading frame 145
206296_x_at	8,37E-06	MAP4K1	mitogen-activated protein kinase kinase kinase kinase 1
216835_s_at	8,57E-06	DOK1	docking protein 1, 62kDa (downstream of tyrosine kinase 1)
239006_at	9,27E-06	SLC26A7	solute carrier family 26, member 7
224913_s_at	1,04E-05	TIMM50	translocase of inner mitochondrial membrane 50 homolog (yeast)
229711_s_at	1,05E-05	MGC5370	hypothetical protein MGC5370
219332_at	1,13E-05	MICAL-L2	MICAL-like 2
214006_s_at	1,18E-05	GGCX	gamma-glutamyl carboxylase
236163_at	1,19E-05	LIX1	Lix1 homolog (mouse)
227974_at	1,27E-05		Transcribed locus, weakly similar to XP_512006.1 PREDICTED: similar to FLJ46489 protein [Pan troglodytes]
244640_at	1,39E-05	LOC342892	Hypothetical protein LOC342892
214428_x_at	1,45E-05	C4A ; C4B	complement component 4A ; complement component 4B ; complement component 4B, telomeric
218651_s_at	1,56E-05	LARP6	La ribonucleoprotein domain family, member 6
202819_s_at	1,76E-05	TCEB3	transcription elongation factor B (SIII), polypeptide 3 (110kDa, elongin A)
223890_at	1,98E-05		
216006_at	2,03E-05		Clone 24694 mRNA sequence
230366_at	2,13E-05	LOC149913	hypothetical LOC149913
236080_at	2,53E-05		Homo sapiens, clone IMAGE:3616855, mRNA
224784_at	2,72E-05	MLLT6	myeloid/lymphoid or mixed-lineage leukemia (trithorax homolog, Drosophila); translocated to, 6
232757_at	2,74E-05	MTSS1	Metastasis suppressor 1
228296_at	2,88E-05	YPEL1	yippee-like 1 (Drosophila)
243612_at	3,01E-05	NSD1	Nuclear receptor binding SET domain protein 1
215723_s_at	3,02E-05	PLD1	phospholipase D1, phophatidylcholine-specific

220201_at	3,40E-05	MNAB	membrane associated DNA binding protein
1562894_at	3,49E-05	ELMO1	Engulfment and cell motility 1 (ced-12 homolog, C. elegans)
219970_at	3,63E-05	GIPC2	GIPC PDZ domain containing family, member 2
231677_at	3,72E-05	AIM1	Absent in melanoma 1
207016_s_at	3,97E-05	ALDH1A2	aldehyde dehydrogenase 1 family, member A2
244774_at	4,22E-05	PHACTR2	Phosphatase and actin regulator 2
227165_at	4,24E-05	C13orf3	chromosome 13 open reading frame 3
1552368_at	4,27E-05	CTCFL	CCCTC-binding factor (zinc finger protein)-like
226857_at	4,40E-05	ARHGEF19	Rho guanine nucleotide exchange factor (GEF) 19
209510_at	4,52E-05	RNF139	ring finger protein 139
231953_at	4,69E-05	FALZ	fetal Alzheimer antigen
213501_at	4,80E-05	ACOX1	acyl-Coenzyme A oxidase 1, palmitoyl
239169_at	4,83E-05	RDM1	RAD52 motif 1
215306_at	5,35E-05	LHCGR	Luteinizing hormone/choriogonadotropin receptor
224947_at	5,64E-05	RNF26	ring finger protein 26
1565834_a_at	5,75E-05		
238919_at	5,84E-05	PCDH9	Protocadherin 9
204374_s_at	5,97E-05	GALK1	galactokinase 1
1553321_a_at	6,80E-05	SULT1C2	sulfotransferase family, cytosolic, 1C, member 2
223384_s_at	6,81E-05	TRIM4	tripartite motif-containing 4
211473_s_at	6,93E-05	COL4A6	collagen, type IV, alpha 6
221467_at	6,95E-05	MC4R	melanocortin 4 receptor
206416_at	7,05E-05	ZNF205	zinc finger protein 205 ; zinc finger protein 205
219132_at	7,12E-05	PELI2	pellino homolog 2 (Drosophila)
1555046_at	7,16E-05	FSHPRH1	FSH primary response (LRPR1 homolog, rat) 1
230854_at	7,20E-05	LOC400500	hypothetical LOC400500
231876_at	7,31E-05	TRIM56	tripartite motif-containing 56
1553138_a_at	7,45E-05	ANKRD41	ankyrin repeat domain 41
226793_at	7,63E-05	LOC283267	hypothetical protein LOC283267
208184_s_at	7,84E-05	TMEM1	transmembrane protein 1
242639_at	7,87E-05	NARG2	NMDA receptor regulated 2
243010_at	8,12E-05	MSI2	musashi homolog 2 (Drosophila)
207501_s_at	8,25E-05	FGF12	fibroblast growth factor 12
220561_at	8,34E-05	IGF2AS	insulin-like growth factor 2 antisense
213009_s_at	8,44E-05	TRIM37	tripartite motif-containing 37
1570039_at	8,48E-05	MGC39900	hypothetical protein MGC39900
1568849_at	8,58E-05		Homo sapiens, Similar to nuclear localization signals binding protein 1, clone IMAGE:3891427, mRNA
211759_x_at	8,58E-05	CKAP1	cytoskeleton associated protein 1 ; cytoskeleton associated protein 1
209631_s_at	8,64E-05	GPR37	G protein-coupled receptor 37 (endothelin receptor type B-like)
221696_s_at	8,69E-05	STYK1	serine/threonine/tyrosine kinase 1 ; serine/threonine/tyrosine kinase 1
226722_at	9,30E-05	FAM20C	family with sequence similarity 20, member C
224591_at	9,35E-05	HP1BP3	heterochromatin protein 1, binding protein 3
221557_s_at	9,57E-05	LEF1	lymphoid enhancer-binding factor 1
200858_s_at	9,72E-05	RPS8	ribosomal protein S8
1569915_at	1,05E-04		CDNA clone IMAGE:5294524
1567686_at	1,05E-04	CECR9	cat eye syndrome chromosome region, candidate 9
219825_at	1,07E-04	CYP26B1	cytochrome P450, family 26, subfamily B, polypeptide 1
244183_x_at	1,08E-04	PCDHB3	protocadherin beta 3
206373_at	1,09E-04	ZIC1	Zic family member 1 (odd-paired homolog, Drosophila)
235333_at	1,12E-04	B4GALT6	UDP-Gal:betaGlcNAc beta 1,4- galactosyltransferase, polypeptide 6
238445_x_at	1,18E-04	MGAT5B	mannosyl (alpha-1,6-)-glycoprotein beta-1,6-N-acetyl-glucosaminyltransferase, isoenzyme B
239231_at	1,23E-04		CDNA FLJ41910 fis, clone PEBLM2007834
211546_x_at	1,26E-04	SNCA	synuclein, alpha (non A4 component of amyloid precursor)
203999_at	1,26E-04	SYT1	synaptotagmin I
224748_at	1,27E-04	WDR68	WD repeat domain 68
218205_s_at	1,31E-04	MKNK2	MAP kinase interacting serine/threonine kinase 2
201998_at	1,36E-04	ST6GAL1	ST6 beta-galactosamide alpha-2,6-sialyltranferase 1
213992_at	1,39E-04	COL4A6	collagen, type IV, alpha 6

227494_at	1,40E-04	LOC253842	hypothetical protein LOC253842
242766_at	1,42E-04		Similar to ankyrin repeat domain 20A
231310_at	1,43E-04		Transcribed locus
224906_at	1,43E-04	TMEM16F	transmembrane protein 16F
211052_s_at	1,45E-04	TBCD	tubulin-specific chaperone d ; tubulin-specific chaperone d
1569878_at	1,46E-04	C10orf21	chromosome 10 open reading frame 21
213191_at	1,48E-04	TICAM1	toll-like receptor adaptor molecule 1
236330_at	1,48E-04		CDNA FLJ42688 fis, clone BRAMY3002120
226081_at	1,49E-04	LZIC	Leucine zipper and CTNNBIP1 domain containing
1563489_at	1,58E-04	LOC285638	hypothetical protein LOC285638
224978_s_at	1,60E-04	USP36	ubiquitin specific peptidase 36
229547_s_at	1,64E-04	WNK2	WNK lysine deficient protein kinase 2
244405_s_at	1,65E-04		Transcribed locus
203973_s_at	1,65E-04	CEBPD	CCAAT/enhancer binding protein (C/EBP), delta
223610_at	1,65E-04	SEMA5B	sema domain, seven thrombospondin repeats (type 1 and type 1-like), transmembrane domain (TM) and short cytoplasmic domain, (semaphorin) 5B
227642_at	1,70E-04	TFCP2L1	Transcription factor CP2-like 1
231720_s_at	1,75E-04	JAM3	junctional adhesion molecule 3
211851_x_at	1,79E-04	BRCA1	breast cancer 1, early onset
212395_s_at	1,80E-04	KIAA0090	KIAA0090
226437_at	1,82E-04	YIF1B	Yip1 interacting factor homolog B (S. cerevisiae)
1566001_at	1,85E-04	ANKRD11	Ankyrin repeat domain 11
234472_at	1,87E-04	GALNT13	UDP-N-acetyl-alpha-D-galactosamine:polypeptide N-acetylgalactosaminyltransferase 13 (GalNAc-T13)
227480_at	1,87E-04	SUSD2	sushi domain containing 2
1568838_at	1,89E-04		CDNA clone IMAGE:5261280
240781_x_at	1,89E-04	RP42	DCN1, defective in cullin neddylation 1, domain containing 1 (S. cerevisiae)
206581_at	1,89E-04	BNC1	basonuclin 1
208124_s_at	1,94E-04	SEMA4F	sema domain, immunoglobulin domain (Ig), transmembrane domain (TM) and short cytoplasmic domain, (semaphorin) 4F
231731_at	1,96E-04	OTX2	orthodenticle homolog 2 (Drosophila)
238732_at	2,00E-04	COL24A1	collagen, type XXIV, alpha 1
242070_at	2,04E-04	LOC400690	hypothetical gene supported by AK092138
243383_at	2,05E-04		Transcribed locus
243386_at	2,06E-04		Similar to cDNA sequence BC035954
232949_at	2,07E-04	CAPZB	Capping protein (actin filament) muscle Z-line, beta
228286_at	2,09E-04	FLJ40869	hypothetical protein FLJ40869
239691_at	2,09E-04	LOC196415	hypothetical protein LOC196415
233216_at	2,09E-04	ZDHHC21	zinc finger, DHHC-type containing 21
229975_at	2,10E-04	BMPR1B	Bone morphogenetic protein receptor, type IB
241224_x_at	2,12E-04	DSCR8	Down syndrome critical region gene 8
213967_at	2,12E-04	LOC138046	hypothetical protein LOC138046
221214_s_at	2,15E-04	NELF	nasal embryonic LHRH factor
236141_at	2,16E-04		Full-length cDNA clone CS0DC029YI23 of Neuroblastoma Cot 25-normalized of Homo sapiens (human)
226575_at	2,20E-04	ZNF462	zinc finger protein 462
214219_x_at	2,25E-04	MAP4K1	mitogen-activated protein kinase kinase kinase kinase 1
213000_at	2,27E-04	MORC3	MORC family CW-type zinc finger 3
235760_at	2,32E-04	NSD1	nuclear receptor binding SET domain protein 1
244070_at	2,39E-04	SYNE1	spectrin repeat containing, nuclear envelope 1
236233_at	2,42E-04	TRIM32	tripartite motif-containing 32
219450_at	2,45E-04	FLJ11017	hypothetical protein FLJ11017
225686_at	2,56E-04	FAM33A	family with sequence similarity 33, member A
237052_x_at	2,60E-04		
244771_at	2,63E-04	KLHDC6	kelch domain containing 6
233153_at	2,69E-04		MRNA; cDNA DKFZp434O1214 (from clone DKFZp434O1214)
228538_at	2,70E-04	ZNF662	zinc finger protein 662
233675_s_at	2,72E-04	LOC374491	TPTE and PTEN homologous inositol lipid phosphatase pseudogene
214338_at	2,76E-04	DNAJB12	DnaJ (Hsp40) homolog, subfamily B, member 12

217782_s_at	2,78E-04	GPS1	G protein pathway suppressor 1
230622_at	2,79E-04	MLLT4	Myeloid/lymphoid or mixed-lineage leukemia (trithorax homolog, Drosophila); translocated to, 4
223514_at	2,82E-04	CARD11	caspase recruitment domain family, member 11
232947_at	2,82E-04	DKK3	Dickkopf homolog 3 (Xenopus laevis)
219488_at	2,83E-04	A4GALT	alpha 1,4-galactosyltransferase (globotriaosylceramide synthase)
219735_s_at	2,86E-04	TFCP2L1	transcription factor CP2-like 1
229812_at	2,89E-04	USP48	Ubiquitin specific peptidase 48
243888_at	2,90E-04		Transcribed locus
200032_s_at	2,90E-04	RPL9	ribosomal protein L9 ; ribosomal protein L9
1555148_a_at	2,91E-04	LOC130951	hypothetical protein BC014602
226660_at	2,91E-04	RPS6KB1	ribosomal protein S6 kinase, 70kDa, polypeptide 1
201804_x_at	2,92E-04	CKAP1	cytoskeleton associated protein 1
223339_at	2,97E-04	ATPIF1	ATPase inhibitory factor 1
221327_s_at	2,98E-04	OPN1MW ; OPN1LW	opsin 1 (cone pigments), medium-wave-sensitive (color blindness, deutan) ; opsin 1 (cone pigments), long-wave-sensitive (color blindness, protan)
205485_at	3,00E-04	RYR1	ryanodine receptor 1 (skeletal)
205351_at	3,00E-04	GGCX	gamma-glutamyl carboxylase
1554076_s_at	3,02E-04	MGC17839	hypothetical protein MGC17839
242344_at	3,04E-04		CDNA clone IMAGE:4814184
226599_at	3,08E-04	KIAA1727	KIAA1727 protein
215860_at	3,08E-04	SYT12	Synaptotagmin XII
1562632_at	3,09E-04	LOC285191	hypothetical protein LOC285191
216194_s_at	3,13E-04	CKAP1	cytoskeleton associated protein 1
214339_s_at	3,14E-04	MAP4K1	mitogen-activated protein kinase kinase kinase kinase 1
236326_at	3,14E-04	HDAC7A	histone deacetylase 7A
212195_at	3,20E-04	IL6ST	Interleukin 6 signal transducer (gp130, oncostatin M receptor)
222777_s_at	3,27E-04	WHSC1	Wolf-Hirschhorn syndrome candidate 1
240218_at	3,27E-04		Transcribed locus
232738_at	3,28E-04		
215957_at	3,29E-04	UBE2D1	ubiquitin-conjugating enzyme E2D 1 (UBC4/5 homolog, yeast)
232827_at	3,30E-04	ITPR3	Inositol 1,4,5-triphosphate receptor, type 3
242002_at	3,31E-04	TCBA1	T-cell lymphoma breakpoint associated target 1
233210_at	3,33E-04	FLJ12120	hypothetical LOC388439
231997_at	3,33E-04	LRRC35	leucine rich repeat containing 35
241726_at	3,36E-04	HLCS	Holocarboxylase synthetase (biotin-(proprionyl-Coenzyme A-carboxylase (ATP-hydrolysing)) ligase)
228441_s_at	3,36E-04		
225175_s_at	3,37E-04	SLC44A2	solute carrier family 44, member 2
239797_at	3,38E-04	CEPT1	Choline/ethanolamine phosphotransferase 1
1561417_x_at	3,41E-04		CDNA FLJ35886 fis, clone TESTI2009091
207307_at	3,42E-04	HTR2C	5-hydroxytryptamine (serotonin) receptor 2C
238932_at	3,43E-04		CDNA FLJ41867 fis, clone OCBBF2005546
242045_at	3,44E-04		Full-length cDNA clone CS0DJ001YE02 of T cells (Jurkat cell line) Cot 10-normalized of Homo sapiens (human)
232380_at	3,47E-04	FLJ10986	hypothetical protein FLJ10986
203057_s_at	3,48E-04	PRDM2	PR domain containing 2, with ZNF domain
1569334_at	3,50E-04	STRA6	stimulated by retinoic acid gene 6 homolog (mouse)
214586_at	3,51E-04	GPR37	G protein-coupled receptor 37 (endothelin receptor type B-like)
233602_at	3,53E-04		Clone 24926 mRNA sequence
206233_at	3,55E-04	B4GALT6	UDP-Gal:betaGlcNAc beta 1,4- galactosyltransferase, polypeptide 6
235413_at	3,56E-04	GGCX	gamma-glutamyl carboxylase
244729_at	3,57E-04		Transcribed locus
239576_at	3,62E-04	MTUS1	Mitochondrial tumor suppressor 1
205597_at	3,65E-04	SLC44A4	solute carrier family 44, member 4
226112_at	3,68E-04	SGCB	sarcoglycan, beta (43kDa dystrophin-associated glycoprotein)
235740_at	3,69E-04	MCTP1	Multiple C2-domains with two transmembrane regions 1
236835_at	3,69E-04	FUT8	fucosyltransferase 8 (alpha (1,6) fucosyltransferase)
215547_at	3,70E-04	TSC22D2	TSC22 domain family, member 2
235050_at	3,73E-04	SLC2A12	solute carrier family 2 (facilitated glucose transporter), member 12
211078_s_at	3,77E-04	STK3	serine/threonine kinase 3 (STE20 homolog, yeast) ;

			serine/threonine kinase 3 (STE20 homolog, yeast)
240406_at	3,78E-04	USP16	Ubiquitin specific peptidase 16
223895_s_at	3,80E-04	EPN3	epsin 3
213184_at	3,87E-04	SENP5	SUMO1/sentrin specific peptidase 5
218281_at	3,87E-04	MRPL48	mitochondrial ribosomal protein L48
214839_at	3,95E-04	LOC157627	hypothetical protein LOC157627
218271_s_at	4,00E-04	PSARL	presenilin associated, rhomboid-like
204915_s_at	4,01E-04	SOX11	SRY (sex determining region Y)-box 11
211767_at	4,04E-04	SLD5	SLD5 homolog ; SLD5 homolog
231846_at	4,07E-04	FLJ23322	hypothetical protein FLJ23322
236728_at	4,09E-04	LNPEP	leucyl/cystinyl aminopeptidase
205417_s_at	4,12E-04	DAG1	dystroglycan 1 (dystrophin-associated glycoprotein 1)
227851_s_at	4,12E-04	PGS1	Dynein, axonemal, heavy polypeptide 17
229470_at	4,16E-04		CDNA FLJ27196 fis, clone SYN02831
223156_at	4,18E-04	MRPS23	mitochondrial ribosomal protein S23
229072_at	4,23E-04		CDNA clone IMAGE:5259272
243190_at	4,23E-04		
217542_at	4,25E-04	CPM	Carboxypeptidase M
214175_x_at	4,26E-04	PDLIM4	PDZ and LIM domain 4
228355_s_at	4,28E-04	mimitin	Myc-induced mitochondria protein
39313_at	4,30E-04	WNK1	WNK lysine deficient protein kinase 1
240759_at	4,34E-04	CENTB2	Centaurin, beta 2
212446_s_at	4,37E-04	LASS6	LAG1 longevity assurance homolog 6 (S. cerevisiae)
228390_at	4,41E-04		CDNA clone IMAGE:5259272
244727_at	4,42E-04	KCNQ1OT1	KCNQ1 overlapping transcript 1
237181_at	4,43E-04	PPP2R5C	Protein phosphatase 2, regulatory subunit B (B56), gamma isoform
202515_at	4,43E-04	DLG1	discs, large homolog 1 (Drosophila)
1566959_at	4,46E-04	GAB2	GRB2-associated binding protein 2
206457_s_at	4,53E-04	DIO1	deiodinase, iodothyronine, type I
211564_s_at	4,53E-04	PDLIM4	PDZ and LIM domain 4
219509_at	4,57E-04	MYOZ1	myozenin 1
220198_s_at	4,60E-04	EIF5A2	eukaryotic translation initiation factor 5A2
225292_at	4,60E-04	COL27A1	collagen, type XXVII, alpha 1
220265_at	4,71E-04	GPR107	G protein-coupled receptor 107
207325_x_at	4,73E-04	MAGEA1	melanoma antigen family A, 1 (directs expression of antigen MZ2-E)
217429_at	4,74E-04	TTLL2	Tubulin tyrosine ligase-like family, member 2
231093_at	4,76E-04	FCRH3	Fc receptor-like 3
1552487_a_at	4,79E-04	BNC1	basonuclin 1
212412_at	4,83E-04	PDLIM5	PDZ and LIM domain 5
1555897_at	4,88E-04	AOF2	amine oxidase (flavin containing) domain 2
242054_s_at	4,91E-04	SIX3	Sine oculis homeobox homolog 3 (Drosophila)
1555071_at	4,91E-04	TLL1	tolloid-like 1
222742_s_at	5,02E-04	RABL5	RAB, member RAS oncogene family-like 5
1557179_s_at	5,03E-04	ING1	Inhibitor of growth family, member 1
1558623_at	5,09E-04		CDNA FLJ40435 fis, clone TESTI2039544
209757_s_at	5,09E-04	MYCN	v-myc myelocytomatosis viral related oncogene, neuroblastoma derived (avian)
217937_s_at	5,16E-04	HDAC7A	histone deacetylase 7A
221407_at	5,19E-04	CX36	connexin-36
1558809_s_at	5,21E-04	LOC284408	hypothetical protein LOC284408
205579_at	5,24E-04	HRH1	histamine receptor H1
227962_at	5,24E-04	ACOX1	Acyl-Coenzyme A oxidase 1, palmitoyl
1556697_at	5,26E-04	LOC285513	hypothetical protein LOC285513
211635_x_at	5,27E-04	IGHV1-69	Immunoglobulin heavy variable 1-69 ; Immunoglobulin heavy variable 1-69
206232_s_at	5,30E-04	B4GALT6	UDP-Gal:betaGlcNAc beta 1,4- galactosyltransferase, polypeptide 6
230116_at	5,30E-04	LOC90133	Keratin 8-like 2
231721_at	5,32E-04	JAM3	junctional adhesion molecule 3
227583_at	5,33E-04	POP4	Processing of precursor 4, ribonuclease P/MRP subunit (S. cerevisiae)
202064_s_at	5,36E-04	SEL1L	sel-1 suppressor of lin-12-like (C. elegans)
220974_x_at	5,36E-04	SFXN3	sideroflexin 3 ; sideroflexin 3

1566301_at	5,38E-04	PPP1R11	Protein phosphatase 1, regulatory (inhibitor) subunit 11
209295_at	5,44E-04	TNFRSF10B	tumor necrosis factor receptor superfamily, member 10b
226414_s_at	5,48E-04	ANAPC11	APC11 anaphase promoting complex subunit 11 homolog (yeast)
1552318_at	5,50E-04	GIMAP1	GTPase, IMAP family member 1
235486_at	5,51E-04	MGC34830	hypothetical protein MGC34830
201522_x_at	5,56E-04	SNRPN ; SNURF	small nuclear ribonucleoprotein polypeptide N ; SNRPN upstream reading frame
1556195_a_at	5,61E-04	LOC400742 ; LOC440572 ; LOC440573	hypothetical gene supported by BC033316 ; hypothetical gene supported by BC033316 ; hypothetical gene supported by BC033316
235648_at	5,66E-04	ZNF567	zinc finger protein 567
200093_s_at	5,67E-04	HINT1	histidine triad nucleotide binding protein 1 ; histidine triad nucleotide binding protein 1
243140_at	5,67E-04	ACTA2	Actin, alpha 2, smooth muscle, aorta
1554553_s_at	5,71E-04	YIF1B	Yip1 interacting factor homolog B (S. cerevisiae)
243008_at	5,74E-04	RHEB	Ras homolog enriched in brain
227956_at	5,81E-04	LOC162073	Hypothetical protein LOC162073
243746_at	5,81E-04		
223495_at	5,84E-04	CCDC8	coiled-coil domain containing 8
204958_at	5,85E-04	PLK3	polo-like kinase 3 (Drosophila)
230727_at	5,94E-04	LOC284106	hypothetical protein LOC284106
210821_x_at	5,98E-04	CENPA	centromere protein A, 17kDa
223597_at	5,99E-04	ITLN1	intelectin 1 (galactofuranose binding)
208345_s_at	6,08E-04	POU3F1	POU domain, class 3, transcription factor 1
211026_s_at	6,14E-04	MGLL	monoglyceride lipase ; monoglyceride lipase
233320_at	6,15E-04	TCAM1	testicular cell adhesion molecule 1 homolog (mouse)
200820_at	6,16E-04	PSMD8	proteasome (prosome, macropain) 26S subunit, non-ATPase, 8
227911_at	6,16E-04	ARHGAP28	Rho GTPase activating protein 28
1568732_at	6,21E-04	COL18A1	Collagen, type XVIII, alpha 1
219299_at	6,26E-04	TRMT12	tRNA methyltranferase 12 homolog (S. cerevisiae)
201919_at	6,30E-04	FLJ10618	Solute carrier family 25, member 36
242932_at	6,30E-04	FARP2	FERM, RhoGEF and pleckstrin domain protein 2
1562458_at	6,31E-04	UBE2W	ubiquitin-conjugating enzyme E2W (putative)
239309_at	6,32E-04	DLX6	distal-less homeo box 6
208451_s_at	6,35E-04	C4A ; C4B	complement component 4A ; complement component 4B ; complement component 4B, telomeric
210428_s_at	6,36E-04	HGS	hepatocyte growth factor-regulated tyrosine kinase substrate
208826_x_at	6,37E-04	HINT1	histidine triad nucleotide binding protein 1
209667_at	6,39E-04	CES2	carboxylesterase 2 (intestine, liver)
238952_x_at	6,51E-04	DKFZp779O175	hypothetical protein DKFZp779O175
1559280_a_at	6,53E-04		CDNA FLJ35259 fis, clone PROST2004251
217811_at	6,62E-04	SELT	selenoprotein T
1570080_at	6,65E-04	HIPK1	Homeodomain interacting protein kinase 1
223681_s_at	6,66E-04	INADL	InaD-like (Drosophila)
205545_x_at	6,66E-04	DNAJC8	DnaJ (Hsp40) homolog, subfamily C, member 8
228724_at	6,71E-04		CDNA clone IMAGE:5312516
232734_at	6,74E-04	TTC23	tetratricopeptide repeat domain 23
223128_at	6,79E-04	H17	hypothetical protein H17
1556144_at	6,82E-04	DHX30	DEAH (Asp-Glu-Ala-His) box polypeptide 30
218730_s_at	6,92E-04	OGN	osteoglycin (osteoinductive factor, mimecan)
202418_at	6,92E-04	YIF1A	Yip1 interacting factor homolog A (S. cerevisiae)
210349_at	7,01E-04	CAMK4	calcium/calmodulin-dependent protein kinase IV
205357_s_at	7,07E-04	AGTR1	angiotensin II receptor, type 1
244274_at	7,10E-04		CDNA clone IMAGE:5269446
221020_s_at	7,12E-04	SLC25A32	solute carrier family 25, member 32 ; solute carrier family 25, member 32
207558_s_at	7,24E-04	PITX2	paired-like homeodomain transcription factor 2
201521_s_at	7,36E-04	NCBP2	nuclear cap binding protein subunit 2, 20kDa
1553016_at	7,42E-04	GPR113	G protein-coupled receptor 113
223242_s_at	7,44E-04	ET	hypothetical protein ET
1555781_at	7,57E-04	PQLC2	PQ loop repeat containing 2
222992_s_at	7,61E-04	NDUFB9	NADH dehydrogenase (ubiquinone) 1 beta subcomplex, 9, 22kDa

241752_at	7,62E-04	SLC8A1	solute carrier family 8 (sodium/calcium exchanger), member 1
227928_at	7,63E-04	FLJ20641	hypothetical protein FLJ20641
223322_at	7,72E-04	RASSF5	Ras association (RalGDS/AF-6) domain family 5
235938_at	7,73E-04		Transcribed locus, weakly similar to XP_517454.1 PREDICTED: similar to hypothetical protein MGC45438 [Pan troglodytes]
207324_s_at	7,78E-04	DSC1	desmocollin 1
215443_at	7,81E-04	TSHR	thyroid stimulating hormone receptor
243907_at	7,81E-04		Transcribed locus
1557488_at	7,84E-04		CDNA clone IMAGE:4798132
230696_at	7,85E-04	ATP2A1	ATPase, Ca++ transporting, cardiac muscle, fast twitch 1
206142_at	7,88E-04	ZNF135	zinc finger protein 135 (clone pHZ-17)
1561017_at	7,90E-04		Full length insert cDNA clone YW28G08
1556653_at	7,92E-04	FLJ25415	hypothetical protein FLJ25415
202940_at	7,99E-04	WNK1	WNK lysine deficient protein kinase 1
1554524_a_at	8,05E-04	OLFM3	olfactomedin 3
233171_at	8,18E-04	GRIN3A	glutamate receptor, ionotropic, N-methyl-D-aspartate 3A
227511_at	8,19E-04	PD2	Paf1, RNA polymerase II associated factor, homolog (S. cerevisiae)
222956_at	8,21E-04	FIGN	fidgetin
234650_at	8,22E-04	LGR4	Leucine-rich repeat-containing G protein-coupled receptor 4
211852_s_at	8,22E-04	ATRN	attractin
236423_at	8,24E-04		Transcribed locus
1558050_at	8,29E-04	EIF2B5	Eukaryotic translation initiation factor 2B, subunit 5 epsilon, 82kDa
236118_at	8,32E-04	LOC201484	hypothetical LOC201484
205558_at	8,33E-04	TRAF6	TNF receptor-associated factor 6
200614_at	8,38E-04	CLTC	clathrin, heavy polypeptide (Hc)
242996_at	8,41E-04	MTRF1	mitochondrial translational release factor 1
230066_at	8,42E-04	SNX25	sorting nexin 25
210503_at	8,44E-04	MAGEA11	melanoma antigen family A, 11
230867_at	8,46E-04	LOC131873	hypothetical protein LOC131873
242560_at	8,51E-04	FANCD2	Fanconi anemia, complementation group D2
230835_at	8,55E-04	UNQ467	KIPV467
233058_at	8,55E-04	MCLC	Chloride channel CLIC-like 1
217613_at	8,57E-04	FLJ11155	hypothetical protein FLJ11155
225933_at	8,72E-04	LOC339229	Hypothetical protein LOC339229
227892_at	8,72E-04		CDNA clone IMAGE:5288757
229622_at	8,72E-04	FLJ37034	hypothetical protein FLJ37034
1557676_at	8,79E-04		CDNA clone IMAGE:4817003
238927_at	8,80E-04		Transcribed locus
219857_at	8,84E-04	C10orf81	chromosome 10 open reading frame 81
223202_s_at	8,84E-04	RP13-360B22.2	hypothetical protein FLJ22679
225516_at	8,93E-04	SLC7A2	solute carrier family 7 (cationic amino acid transporter, y+ system), member 2
239786_at	9,02E-04		Transcribed locus
205199_at	9,04E-04	CA9	carbonic anhydrase IX
1562411_at	9,05E-04	MLCK	cardiac-MyBP-C associated Ca/CaM kinase
213600_at	9,08E-04	SIPA1L3	signal-induced proliferation-associated 1 like 3
204254_s_at	9,11E-04	VDR	vitamin D (1,25- dihydroxyvitamin D3) receptor
209114_at	9,12E-04	TSPAN1	tetraspanin 1
206149_at	9,12E-04	LOC63928	hepatocellular carcinoma antigen gene 520
244566_at	9,22E-04	ARID4A	AT rich interactive domain 4A (RBP1-like)
211634_x_at	9,22E-04	IGHM	immunoglobulin heavy constant mu ; immunoglobulin heavy constant mu
234733_s_at	9,23E-04		
239190_at	9,30E-04	VRK3	vaccinia related kinase 3
240224_at	9,35E-04	PRDM10	PR domain containing 10
232572_at	9,37E-04	KIAA0367	KIAA0367
235350_at	9,39E-04		Transcribed locus
222287_at	9,40E-04	TRDN	triadin
220078_at	9,42E-04	USP48	ubiquitin specific peptidase 48
223316_at	9,43E-04	CCDC3	coiled-coil domain containing 3
219281_at	9,49E-04	MSRA	methionine sulfoxide reductase A
207984_s_at	9,50E-04	MPP2	membrane protein, palmitoylated 2 (MAGUK p55 subfamily member

			2)
229021_at	9,50E-04	MCTP2	Multiple C2-domains with two transmembrane regions 2
240000_at	9,51E-04	LIPI	Lipase, member I
228273_at	9,59E-04	FLJ11029	Hypothetical protein FLJ11029
235608_at	9,62E-04	CLN5	Ceroid-lipofuscinosis, neuronal 5
224175_s_at	9,64E-04	TRIM34 ; TRIM6-TRIM34	tripartite motif-containing 34 ; tripartite motif-containing 6 and tripartite motif-containing 34
218051_s_at	9,64E-04	FLJ12442	hypothetical protein FLJ12442
218983_at	9,66E-04	C1RL	complement component 1, r subcomponent-like
230119_at	9,68E-04		CDNA FLJ43434 fis, clone OCBBF2028055
206306_at	9,77E-04	RYR3	ryanodine receptor 3
224984_at	9,77E-04	NFAT5	nuclear factor of activated T-cells 5, tonicity-responsive
220264_s_at	9,84E-04	GPR107	G protein-coupled receptor 107
238149_at	9,93E-04	FLJ46385	FLJ46385 protein
220915_s_at	9,97E-04		MRNA similar to hypothetical protein FLJ11871 (cDNA clone IMAGE:6160816)
229621_x_at	9,99E-04	DKFZp667B0210	Early B-cell factor 3
239062_at	1,00E-03		Clone FP3361 unknown mRNA
