# Supplementary material for: Gene Expression Analysis in Ovarian Cancer – Faults and Hints from DNA Microarray Study
Source: Front Oncol. 2014 Jan 28;4:6. doi: 10.3389/fonc.2014.00006 (PMC3904181; doi:10.3389/fonc.2014.00006)
Supplement: Supplementary file 7 [file 61993_Lisowska_DataSheet7.PDF]

Tumor grade

1. Hierarchical clustering: Dendrogram showes the relations between tumor samples of different grade. Heat map shows relative intensity of gene expression. Grade 2 samples are clustered together (except one), in the left branch of dendrogram and show distinct gene expression pattern. Grade 3 and grade 4 samples are mixed together and do not show distinct gene expression pattern.

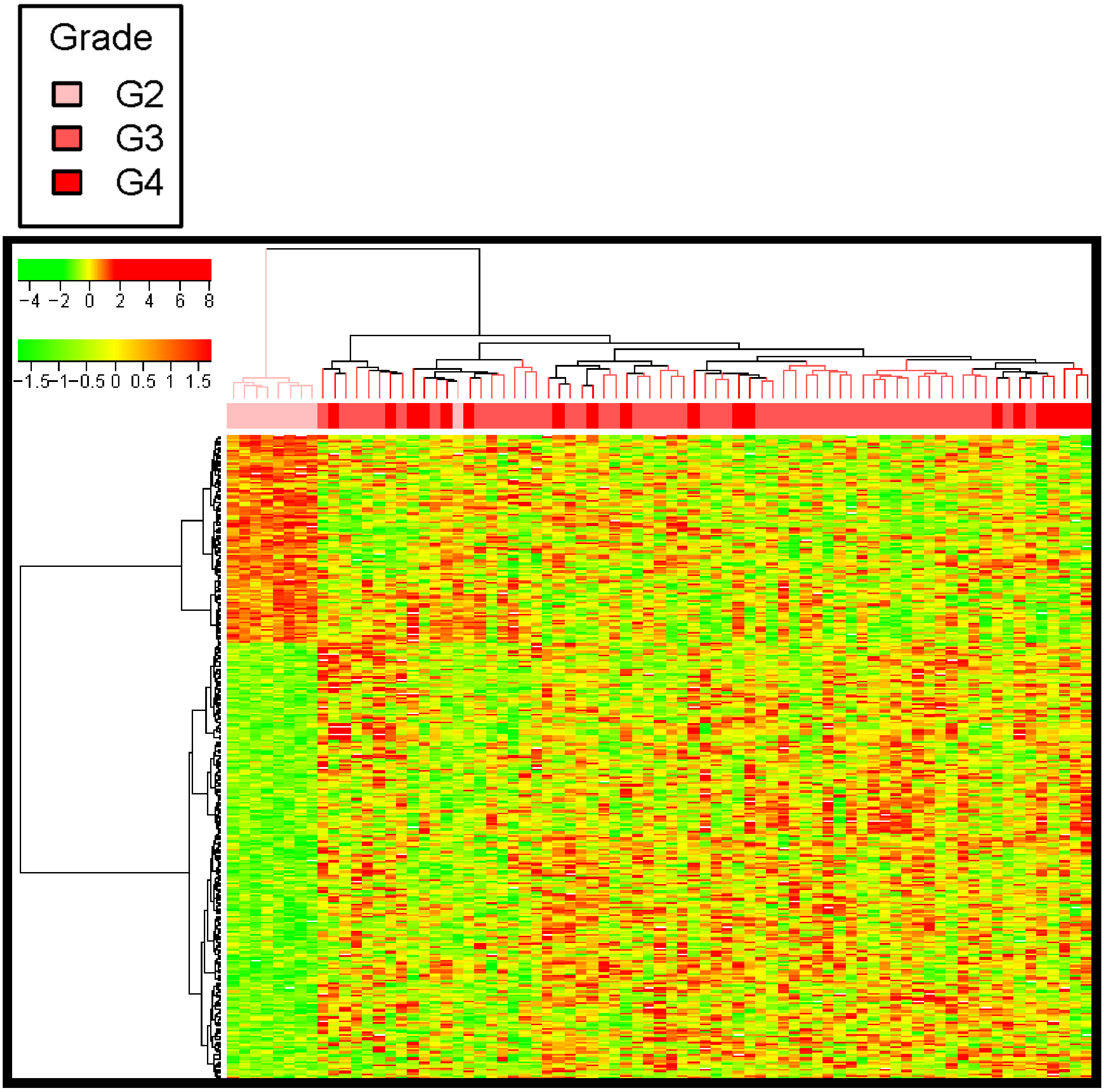

2. Gene Ontology analysis was performed using annotated genes selected in Welch (p<0.001) for the difference between grade 2 versus grade 3/4 ovarian cancers.

Gene Ontology class	p-value
GO:7292: female gamete generation	0.000965
GO:30183: B cell differentiation	0.000826
GO:42386: hemocyte differentiation (sensu Arthropoda)	7.78e-5
GO:45610: regulation of hemocyte differentiation	1.2e-7
GO:48534: hemopoietic or lymphoid organ development	0.00078
GO:30097: hemopoiesis	0.00078
GO:6464: protein modification	0.000497
GO:18105: peptidyl-serine phosphorylation	6.43e-5
GO:16571: histone methylation	0.000674
GO:18193: peptidyl-amino acid modification	3.93e-5

GO:18200: peptidyl-glutamic acid modification	4.8e-7
GO:17187: peptidyl-glutamic acid carboxylation	4.8e-7
GO:18209: peptidyl-serine modification	6.43e-5
GO:18214: protein amino acid carboxylation	4.8e-7
GO:43412: biopolymer modification	0.000826
GO:7224: smoothened signaling pathway	0.000363
GO:8589: regulation of smoothened signaling pathway	0.000363
GO:185: activation of MAPKKK activity	5.24e-5
GO:43405: regulation of MAPK activity	0.000808

3. Signaling pathways (according to Biocarta repository) with significantly changed gene expression between grade 2 versus grade 3/4 ovarian cancers. Least square (LS) and Kolomogorov-Smirnoff (KS) tests were used for analysis of annotated genes selected in Welch (p<0.001) for the difference between grade 2 versus grade 3/4 ovarian cancers.

	**Signaling pathways (Biocarta)**	**Signaling pathway name**	**p-value LS**	**p-value KS**
1	h_cdc25Pathway	cdc25 and chk1 Regulatory Pathway in response to DNA damage	1e-05	0.0413098
2	h_rbPathway	RB Tumor Suppressor/Checkpoint Signaling in response to DNA damage	9.54e-05	0.1844022
3	h_srcRPTPPathway	Activation of Src by Protein-tyrosine phosphatase alpha	9.92e-05	0.0354198
4	h_ptc1Pathway	Sonic Hedgehog (SHH) Receptor Ptc1 Regulates cell cycle	0.0003439	0.0036253
5	h_g2Pathway	Cell Cycle: G2/M Checkpoint	0.0003785	0.0634496
6	h_atrbrcaPathway	Role of BRCA1, BRCA2 and ATR in Cancer Susceptibility	0.0011847	0.0395389
7	h_akap95Pathway	AKAP95 role in mitosis and chromosome dynamics	0.0011875	0.1764421
8	h_cblPathway	CBL mediated ligand-induced downregulation of EGF receptors	0.0018191	0.00064
9	h_il22bppathway	IL22 Soluble Receptor Signaling Pathway	0.0018852	0.0007607
10	h_stathminPathway	Stathmin and breast cancer resistance to antimicrotubule agents	0.001936	0.0983181
11	h_bcellsurvivalPathway	B Cell Survival Pathway	0.0021628	0.0053152
12	h_cardiacegfPathway	Role of EGF Receptor Transactivation by GPCRs in Cardiac Hypertrophy	0.0023027	0.0169817
13	h_atmPathway	ATM Signaling Pathway	0.0027988	0.0634496
14	h_plk3Pathway	Regulation of cell cycle progression by Plk3	0.0032204	0.1823807
15	h_sam68Pathway	Regulation of Splicing through Sam68	0.0034882	0.2623317
16	h_ace2Pathway	Angiotensin-converting enzyme 2 regulates heart function	0.0039638	0.5244766
17	h_ctla4Pathway	The Co-Stimulatory Signal During T-cell Activation	0.232106	0.002739
18	h_asbcellPathway	Antigen Dependent B Cell Activation	0.2860272	0.0047681
19	h_bbcellPathway	Bystander B Cell Activation	0.2860272	0.0047681
20	h_eosinophilsPathway	The Role of Eosinophils in the Chemokine Network of Allergy	0.4755411	0.0049615

4. Signaling pathways (according to Biocarta repository) with significantly changed gene expression between grade 2 versus grade 3/4 ovarian cancers. Least square (LS) and Kolomogorov-Smirnoff (KS) tests were used for analysis of annotated genes selected in Welch test (p<0.001) for the difference between grade 2 versus grade 3/4 ovarian cancers.

	**Signaling pathways (Biocarta)**	**Signaling pathway name**	**p-value**
1	h_metPathway	Signaling of Hepatocyte Growth Factor Receptor	2.13e-05
2	h_tffPathway	Trefoil Factors Initiate Mucosal Healing	2.72e-05
3	h_cdc25Pathway	cdc25 and chk1 Regulatory Pathway in response to DNA damage	0.0001118
4	h_stemPathway	Regulation of hematopoiesis by cytokines	0.0001209
5	h_bcellsurvivalPathway	B Cell Survival Pathway	0.0001348
6	h_edg1Pathway	Phospholipids as signalling intermediaries	0.0001417

7	h_erkPathway	Erk1/Erk2 Mapk Signaling pathway	0.0001456
8	h_akapCentrosomePathway	Protein Kinase A at the Centrosome	0.0001598
9	h_ghPathway	Growth Hormone Signaling Pathway	0.000199
10	h_ptc1Pathway	Sonic Hedgehog (SHH) Receptor Ptc1 Regulates cell cycle	0.0002605
11	h_amiPathway	Acute Myocardial Infarction	0.000286
12	h_srcRPTPPathway	Activation of Src by Protein-tyrosine phosphatase alpha	0.0004525
13	h_igf1Pathway	IGF-1 Signaling Pathway	0.0004526
14	h_cblPathway	CBL mediated ligand-induced downregulation of EGF receptors	0.0004843
15	h_At1rPathway	Angiotensin II mediated activation of JNK Pathway via Pyk2 dependent signaling	0.0005084
16	h_longevityPathway	The IGF-1 Receptor and Longevity	0.0005154
17	h_rbPathway	RB Tumor Suppressor/Checkpoint Signaling in response to DNA damage	0.0005456
18	h_agrPathway	Agrin in Postsynaptic Differentiation	0.0005758
19	h_sumoPathway	Basic Mechanisms of SUMOylation	0.0009287
20	h_mPRPathway	How Progesterone Initiates the Oocyte Maturation	0.0009723
21	h_il7Pathway	IL-7 Signal Transduction	0.0010085
22	h_eponfkbPathway	Erythropoietin mediated neuroprotection through NF-kB	0.0010893
23	h_g2Pathway	Cell Cycle: G2/M Checkpoint	0.0011699
24	h_s1pPathway	SREBP control of lipid synthesis	0.0013423
25	h_akap95Pathway	AKAP95 role in mitosis and chromosome dynamics	0.0014676
26	h_intrinsicPathway	Intrinsic Prothrombin Activation Pathway	0.0014892
27	h_spryPathway	Sprouty regulation of tyrosine kinase signals	0.00156
28	h_atrbrcaPathway	Role of BRCA1, BRCA2 and ATR in Cancer Susceptibility	0.0020663
29	h_gleevecpathway	Inhibition of Cellular Proliferation by Gleevec	0.0023232
30	h_il22bppathway	IL22 Soluble Receptor Signaling Pathway	0.0023837
31	h_WNVpathway	West Nile Virus	0.0026908
32	h_dspPathway	Regulation of MAP Kinase Pathways Through Dual Swoistość Phosphatases	0.0028588
33	h_plk3Pathway	Regulation of cell cycle progression by Plk3	0.0030311
34	h_sam68Pathway	Regulation of Splicing through Sam68	0.0031397
35	h_bard1Pathway	BRCA1-dependent Ub-ligase activity	0.0032425
36	h_cdc42racPathway	Role of PI3K subunit p85 in regulation of Actin Organization and Cell Migration	0.0036829
37	h_atmPathway	ATM Signaling Pathway	0.0038717
38	h_Ccr5Pathway	Pertussis toxin-insensitive CCR5 Signaling in Macrophage	0.0038779
39	h_her2Pathway	Role of ERBB2 in Signal Transduction and Oncology	0.0047227
40	h_lympathway	Adhesion and Diapedesis of Lymphocytes	0.0048539
