# Supplementary material for: Gene Expression Analysis in Ovarian Cancer – Faults and Hints from DNA Microarray Study
Source: Front Oncol. 2014 Jan 28;4:6. doi: 10.3389/fonc.2014.00006 (PMC3904181; doi:10.3389/fonc.2014.00006)
Supplement: Supplementary file 8 [file 61993_Lisowska_DataSheet8.PDF]

Supplementary table 5. Cytoreduction/Residual tumor size

List of genes with significantly changed expression in relation to the residual tumor size; 212 probe sets with p<0.001 were selected in Welch test for comparison of R0 *versus* R1/R2 tumors (R0<1cm, 5cm>R1>1 cm, R2>5cm).

Affymetrix probe set ID	p-value	Gene symbol	Gene name
1558101_at	9,11E-08		CDNA FLJ39164 fis, clone OCBBF2002656
217789_at	2,08E-06	SNX6	sorting nexin 6
239571_at	7,06E-06	MEF2A	MADS box transcription enhancer factor 2, polypeptide A (myocyte enhancer factor 2A)
240600_at	9,46E-06	AP3B1	Adaptor-related protein complex 3, beta 1 subunit
240405_at	1,16E-05	CASQ1	Calsequestrin 1 (fast-twitch, skeletal muscle)
218725_at	1,48E-05	SLC25A22	solute carrier family 25 (mitochondrial carrier: glutamate), member 22
235281_x_at	1,55E-05	AHNAK	AHNAK nucleoprotein (desmoyokin)
236665_at	1,66E-05	CCDC18	coiled-coil domain containing 18
217675_at	2,07E-05	APM-1	BTB/POZ-zinc finger protein-like
215668_s_at	2,11E-05	PLXNB1	plexin B1
1554152_a_at	2,69E-05	OGDH	oxoglutarate (alpha-ketoglutarate) dehydrogenase (lipoamide)
209872_s_at	3,01E-05	PKP3	plakophilin 3
235795_at	3,08E-05	PAX6	paired box gene 6 (aniridia, keratitis)
216926_s_at	3,27E-05	KIAA0892	KIAA0892
1565811_at	3,36E-05		
216997_x_at	4,03E-05	TLE4	transducin-like enhancer of split 4 (E(sp1) homolog, Drosophila)
1557965_at	4,53E-05	MTERFD2	MTERF domain containing 2
222501_s_at	4,75E-05	REPIN1	replication initiator 1
216361_s_at	4,90E-05	MYST3	MYST histone acetyltransferase (monocytic leukemia) 3
217620_s_at	5,27E-05	PIK3CB	phosphoinositide-3-kinase, catalytic, beta polypeptide
205646_s_at	5,42E-05	PAX6	paired box gene 6 (aniridia, keratitis)
201638_s_at	5,78E-05	CPSF1	cleavage and polyadenylation specific factor 1, 160kDa
211199_s_at	5,95E-05	ICOSLG	inducible T-cell co-stimulator ligand
231299_at	6,25E-05	CENTG3	centaurin, gamma 3
229294_at	6,42E-05	JPH3	junctophilin 3
237673_at	6,72E-05	TGFB1I4	TSC22 domain family, member 1
235594_at	7,09E-05	SCYE1	Small inducible cytokine subfamily E, member 1 (endothelial monocyte-activating)
240551_at	7,13E-05	ZNF499	zinc finger protein 499
235412_at	7,53E-05	ARHGEF7	Rho guanine nucleotide exchange factor (GEF) 7
213756_s_at	8,13E-05	HSF1	heat shock transcription factor 1
225869_s_at	8,22E-05	UNC93B1	unc-93 homolog B1 (C. elegans)
222546_s_at	8,25E-05	EPS8L2	EPS8-like 2
240141_at	9,83E-05	PAPD4	PAP associated domain containing 4
1553881_at	0,0001	MGC16142	hypothetical protein MGC16142
231939_s_at	0,000105	BDP1	B double prime 1, subunit of RNA polymerase III transcription initiation factor IIIB
242752_at	0,000107		MRNA (clone ICRFp507I1077)
226401_at	0,000112	PARP10	poly (ADP-ribose) polymerase family, member 10
216153_x_at	0,000112	RECK	reversion-inducing-cysteine-rich protein with kazal motifs
1556658_a_at	0,000112	MBNL1	Muscleblind-like (Drosophila)
202476_s_at	0,000112	TUBGCP2	tubulin, gamma complex associated protein 2
209428_s_at	0,000113	ZFPL1	zinc finger protein-like 1
1556834_at	0,000121		CDNA clone IMAGE:5296106
232340_at	0,000123		Hypothetical LOC388889
233436_at	0,000136	MTBP	Mdm2, transformed 3T3 cell double minute 2, p53 binding protein (mouse) binding protein, 104kDa
228158_at	0,000139		PREDICTED: Homo sapiens similar to D(1B) dopamine receptor (D(5) dopamine receptor) (D1beta dopamine receptor) (LOC440684), mRNA
1553162_x_at	0,000147	LOC148137	hypothetical protein BC017947
1555784_s_at	0,000148	IRAK1	interleukin-1 receptor-associated kinase 1
219522_at	0,000148	FJX1	four jointed box 1 (Drosophila)
238243_at	0,000149	CCNH	Cyclin H
220085_at	0,000156	HELLS	helicase, lymphoid-specific
213176_s_at	0,000164	LTBP4	latent transforming growth factor beta binding protein 4

208769_at	0,000167	EIF4EBP2	Eukaryotic translation initiation factor 4E binding protein 2
244181_at	0,000174	PIK3R1	Phosphoinositide-3-kinase, regulatory subunit 1 (p85 alpha)
1561112_at	0,000175		CDNA clone IMAGE:5299117
212139_at	0,000176	GCN1L1	GCN1 general control of amino-acid synthesis 1-like 1 (yeast)
209182_s_at	0,000179	C10orf10	chromosome 10 open reading frame 10
230271_at	0,00018		Homo sapiens, clone IMAGE:4512785, mRNA
226845_s_at	0,000184	MYEOV2	myeloma overexpressed 2
1552611_a_at	0,000188	JAK1	Janus kinase 1 (a protein tyrosine kinase)
236696_at	0,00019	SR140	U2-associated SR140 protein
1560339_s_at	0,000193	NAP1L4	nucleosome assembly protein 1-like 4
239960_x_at	0,000203		CDNA FLJ32429 fis, clone SKMUS2001014
200999_s_at	0,000209	CKAP4	cytoskeleton-associated protein 4
224759_s_at	0,000212	MGC17943	hypothetical protein MGC17943
210362_x_at	0,000213	PML	promyelocytic leukemia
216549_s_at	0,000216	TBC1D22B	TBC1 domain family, member 22B
219723_x_at	0,000238	AGPAT3	1-acylglycerol-3-phosphate O-acyltransferase 3
1554311_a_at	0,000239	SUPT6H	suppressor of Ty 6 homolog (S. cerevisiae)
2028_s_at	0,000243	E2F1	E2F transcription factor 1
1570061_at	0,000248		CDNA clone IMAGE:4555030
238563_at	0,000254	ABI1	Abl-interactor 1
238320_at	0,000254	TncRNA	trophoblast-derived noncoding RNA
1557966_x_at	0,000254	MTERFD2	MTERF domain containing 2
228764_s_at	0,000255	CHMP4A ; MGC5987	chromatin modifying protein 4A ; hypothetical protein MGC5987
218824_at	0,000259	FLJ10781	hypothetical protein FLJ10781
208624_s_at	0,000264	EIF4G1	eukaryotic translation initiation factor 4 gamma, 1
209151_x_at	0,000276	TCF3	transcription factor 3 (E2A immunoglobulin enhancer binding factors E12/E47)
218855_at	0,000282	GPR175	G protein-coupled receptor 175
223578_x_at	0,000284	PRO1073	PRO1073 protein
244016_at	0,000284		Transcribed locus
216069_at	0,000284	HRMT1L1	HMT1 hnRNP methyltransferase-like 1 (S. cerevisiae)
1557749_at	0,000294	EHBP1L1	EH domain binding protein 1-like 1
1553652_a_at	0,000313	C18orf54	chromosome 18 open reading frame 54
222650_s_at	0,00033	SLC2A4RG	SLC2A4 regulator
222816_s_at	0,000332	ZCCHC2	zinc finger, CCHC domain containing 2
225295_at	0,000333	SLC39A10	solute carrier family 39 (zinc transporter), member 10
236621_at	0,000335	RPS27	ribosomal protein S27 (metallopanstimulin 1)
1564699_at	0,000345	C5orf4	Chromosome 5 open reading frame 4
1569477_at	0,000346	FOXO3A	Forkhead box O3A
1555611_s_at	0,00035	MBD1	methyl-CpG binding domain protein 1
235526_at	0,00035	SMAP	Small acidic protein
224078_at	0,000355	HIATL2	hippocampus abundant gene transcript-like 2
239810_at	0,000356	VASH1	vasohibin 1
242163_at	0,00036	THRAP3	thyroid hormone receptor associated protein 3
213201_s_at	0,000364	TNNT1	troponin T type 1 (skeletal, slow)
224628_at	0,000374	C2orf30	chromosome 2 open reading frame 30
230393_at	0,000395	CUL5	Cullin 5
205457_at	0,000398	C6orf106	chromosome 6 open reading frame 106
206997_s_at	0,0004	HS6ST1 ; HS6ST1P	heparan sulfate 6-O-sulfotransferase 1 ; heparan sulfate 6-O-sulfotransferase 1 pseudogene
1554986_a_at	0,000401	SNX19	sorting nexin 19
239332_at	0,000403		Homo sapiens, clone IMAGE:3897156, mRNA
238750_at	0,000407	CCL28	Chemokine (C-C motif) ligand 28
209613_s_at	0,000409	ADH1B	alcohol dehydrogenase IB (class I), beta polypeptide
224563_at	0,000414	WASF2	WAS protein family, member 2
209776_s_at	0,000416	SLC19A1	solute carrier family 19 (folate transporter), member 1
209930_s_at	0,000419	NFE2	nuclear factor (erythroid-derived 2), 45kDa
228763_at	0,000428	CHMP4A ; MGC5987	chromatin modifying protein 4A ; hypothetical protein MGC5987
242628_at	0,000432	KLRB1	Killer cell lectin-like receptor subfamily B, member 1
226405_s_at	0,000437	ARRDC1	arrestin domain containing 1

1563345_at	0,000437		
1556345_s_at	0,000449	MCCC2	Methylcrotonoyl-Coenzyme A carboxylase 2 (beta)
204815_s_at	0,00045	DHX34	DEAH (Asp-Glu-Ala-His) box polypeptide 34
212509_s_at	0,000456	MXRA7	matrix-remodelling associated 7
224064_s_at	0,000469	DHDDS	dehydrodolichyl diphosphate synthase
1555565_s_at	0,000474	TAPBP	TAP binding protein (tapasin)
243293_at	0,000475		Transcribed locus
204027_s_at	0,000476	METTL1	methyltransferase like 1
1556373_a_at	0,000479	MGC35361	Hypothetical protein MGC35361
213455_at	0,000483	LOC283677	hypothetical LOC283677
1555639_a_at	0,000493	RBM14	RNA binding motif protein 14
200046_at	0,000496	DAD1	defender against cell death 1 ; defender against cell death 1
215357_s_at	0,000497	POLDIP3	polymerase (DNA-directed), delta interacting protein 3
238848_at	0,000507	OTUD4	OTU domain containing 4
1559957_a_at	0,000509		Homo sapiens, clone IMAGE:4581955
215079_at	0,00051		LOC441207
228360_at	0,000513	LOC130576	hypothetical protein LOC130576
207793_s_at	0,000514	EPB41	erythrocyte membrane protein band 4.1 (elliptocytosis 1, RH-linked)
1568680_s_at	0,00053	YTHDC2	YTH domain containing 2
240899_at	0,000535		Transcribed locus, moderately similar to XP_517655.1 PREDICTED: similar to KIAA0825 protein [Pan troglodytes]
232356_at	0,000546	KIAA0143	KIAA0143 protein
206511_s_at	0,000547	SIX2	sine oculis homeobox homolog 2 (Drosophila)
215605_at	0,000548	NCOA2	Nuclear receptor coactivator 2
222504_s_at	0,000556	COX4NB	COX4 neighbor
1557970_s_at	0,000558	RPS6KA2	ribosomal protein S6 kinase, 90kDa, polypeptide 2
218382_s_at	0,000562	U2AF2	U2 (RNU2) small nuclear RNA auxiliary factor 2
234730_s_at	0,000565	RIPK4	receptor-interacting serine-threonine kinase 4
221058_s_at	0,000574	CKLF	chemokine-like factor
33646_g_at	0,000575	GM2A	GM2 ganglioside activator
238506_at	0,000579	LOC116064	hypothetical protein LOC116064
211453_s_at	0,000579	AKT2	v-akt murine thymoma viral oncogene homolog 2
237333_at	0,000586	SYNCOILIN	Syncoilin, intermediate filament 1
231436_at	0,000592		
220521_s_at	0,000593	ATG16L1	ATG16 autophagy related 16-like 1 (S. cerevisiae)
201282_at	0,000599	OGDH	oxoglutarate (alpha-ketoglutarate) dehydrogenase (lipoamide)
231109_at	0,000602	CUGBP2	CUG triplet repeat, RNA binding protein 2
224284_x_at	0,000603	LOC441446	similar to FKSG60
1560817_at	0,000618	MOV10	Mov10, Moloney leukemia virus 10, homolog (mouse)
242485_at	0,000618	PTK2	PTK2 protein tyrosine kinase 2
234675_x_at	0,000624		CDNA: FLJ23566 fis, clone LNG10880
207043_s_at	0,000625	SLC6A9	solute carrier family 6 (neurotransmitter transporter, glycine), member 9
216222_s_at	0,000634	MYO10	myosin X
32837_at	0,000636	AGPAT2	1-acylglycerol-3-phosphate O-acyltransferase 2 (lysophosphatidic acid acyltransferase, beta)
213209_at	0,000656	TAF6L	TAF6-like RNA polymerase II, p300/CBP-associated factor (PCAF)-associated factor, 65kDa
229541_at	0,000671		
224630_at	0,000676	C2orf30	chromosome 2 open reading frame 30
202549_at	0,000678	VAPB	VAMP (vesicle-associated membrane protein)-associated protein B and C
202614_at	0,000683	SLC30A9	solute carrier family 30 (zinc transporter), member 9
226893_at	0,000693	ABL2	V-abl Abelson murine leukemia viral oncogene homolog 2 (arg, Abelson-related gene)
239405_at	0,000694	RAB7	RAB7, member RAS oncogene family
201367_s_at	0,000694	ZFP36L2	zinc finger protein 36, C3H type-like 2
219611_s_at	0,000696	CCDC21	coiled-coil domain containing 21
206104_at	0,000701	ISL1	ISL1 transcription factor, LIM/homeodomain, (islet-1)
237477_at	0,000712	PDCL2	phosducin-like 2
232729_at	0,000713	FBXO32	F-box protein 32
237766_at	0,000722	ATP9B	ATPase, Class II, type 9B
242854_x_at	0,000724		Full length insert cDNA YR33G07

1553668_at	0,000728	LRCH3	leucine-rich repeats and calponin homology (CH) domain containing 3
220176_at	0,00073	NUBPL	nucleotide binding protein-like
211709_s_at	0,000734	CLEC11A	C-type lectin domain family 11, member A ; C-type lectin domain family 11, member A
221785_at	0,00074	WIZ	widely-interspaced zinc finger motifs
213121_at	0,00074	SNRP70	small nuclear ribonucleoprotein 70kDa polypeptide (RNP antigen)
212362_at	0,000745	ATP2A2	ATPase, Ca++ transporting, cardiac muscle, slow twitch 2
218064_s_at	0,000746	AKAP8L	A kinase (PRKA) anchor protein 8-like
226442_at	0,000754	ABTB1	ankyrin repeat and BTB (POZ) domain containing 1
205426_s_at	0,000756	HIP1	huntingtin interacting protein 1
219152_at	0,000772	PODXL2	podocalyxin-like 2
215581_s_at	0,000774	MCM3AP	MCM3 minichromosome maintenance deficient 3 (S. cerevisiae) associated protein
1563321_s_at	0,000779	MLLT10	myeloid/lymphoid or mixed-lineage leukemia (trithorax homolog, Drosophila); translocated to, 10
213182_x_at	0,00078	CDKN1C	cyclin-dependent kinase inhibitor 1C (p57, Kip2)
1558733_at	0,000783	ZBTB38	zinc finger and BTB domain containing 38
215833_s_at	0,000785	SPPL2B	signal peptide peptidase-like 2B
227918_s_at	0,00079	ZYG11B	zyg-11 homolog B (C. elegans)
212005_at	0,000814	C1orf144	chromosome 1 open reading frame 144
214216_s_at	0,000815	LARP5	La ribonucleoprotein domain family, member 5
215037_s_at	0,000829	BCL2L1	BCL2-like 1
224928_at	0,000841	SET7	SET domain-containing protein 7
239818_x_at	0,000842	TRIB1	tribbles homolog 1 (Drosophila)
208801_at	0,000851	SRP72	signal recognition particle 72kDa
241661_at	0,000854	JMJD1C	jumonji domain containing 1C
235730_at	0,000857		CDNA FLJ34425 fis, clone HHDPC2008297
233304_at	0,000861	NFIB	Nuclear factor I/B
1565999_at	0,000878	LOC286299	hypothetical protein LOC286299
207760_s_at	0,000882	NCOR2	nuclear receptor co-repressor 2
222872_x_at	0,000889	FLJ22833	hypothetical protein FLJ22833
1558173_a_at	0,000897	LUZP1	leucine zipper protein 1
227078_at	0,000902	MGC52022	Musculoskeletal, embryonic nuclear protein 1
242890_at	0,00091	HELLS	Helicase, lymphoid-specific
215620_at	0,000916	RREB1	ras responsive element binding protein 1
239911_at	0,000921		Full-length cDNA clone CS0DJ013YP06 of T cells (Jurkat cell line) Cot 10-normalized of Homo sapiens (human)
203942_s_at	0,000924	MARK2	MAP/microtubule affinity-regulating kinase 2
220602_s_at	0,000926	FLJ22795 ; LOC388152 ; LOC388161	hypothetical protein FLJ22795 ; hypothetical protein FLJ90297 ; LOC388161
236558_at	0,000932	MBNL1	Muscleblind-like (Drosophila)
66053_at	0,000936	HNRPUL2	heterogeneous nuclear ribonucleoprotein U-like 2
208144_s_at	0,000936		
235581_at	0,000946		Transcribed locus, weakly similar to NP_990417.1 ferritin H chain [Gallus gallus]
228760_at	0,000953	SRP46	Splicing factor, arginine/serine-rich, 46kD
235167_at	0,000964	DKFZp547E087	hypothetical gene LOC283846
205197_s_at	0,000967	ATP7A	ATPase, Cu++ transporting, alpha polypeptide (Menkes syndrome)
209113_s_at	0,000973	HMG20B	high-mobility group 20B
233085_s_at	0,000974	FLJ22833	hypothetical protein FLJ22833
216894_x_at	0,000985	CDKN1C	cyclin-dependent kinase inhibitor 1C (p57, Kip2)
1556657_at	0,000986	MBNL1	Muscleblind-like (Drosophila)
