# Supplementary material for: Gene Expression Analysis in Ovarian Cancer – Faults and Hints from DNA Microarray Study
Source: Front Oncol. 2014 Jan 28;4:6. doi: 10.3389/fonc.2014.00006 (PMC3904181; doi:10.3389/fonc.2014.00006)
Supplement: Supplementary file 9 [file 61993_Lisowska_DataSheet9.PDF]

## Residual tumor size

**1. Hierarchical clustering:** Dendrogram showes the relations between tumor samples from patients with different residual tumor size (R0<1cm, 5cm>R1>1cm, R2>5cm). Heat map shows relative intensity of genes expression. Samples are clustered into two main branches with distinct gene expression pattern, however this division seems unrelated with the size of residual tumor: samples R0, R1 and R2 are mixed together in both branches of the dendrogram.

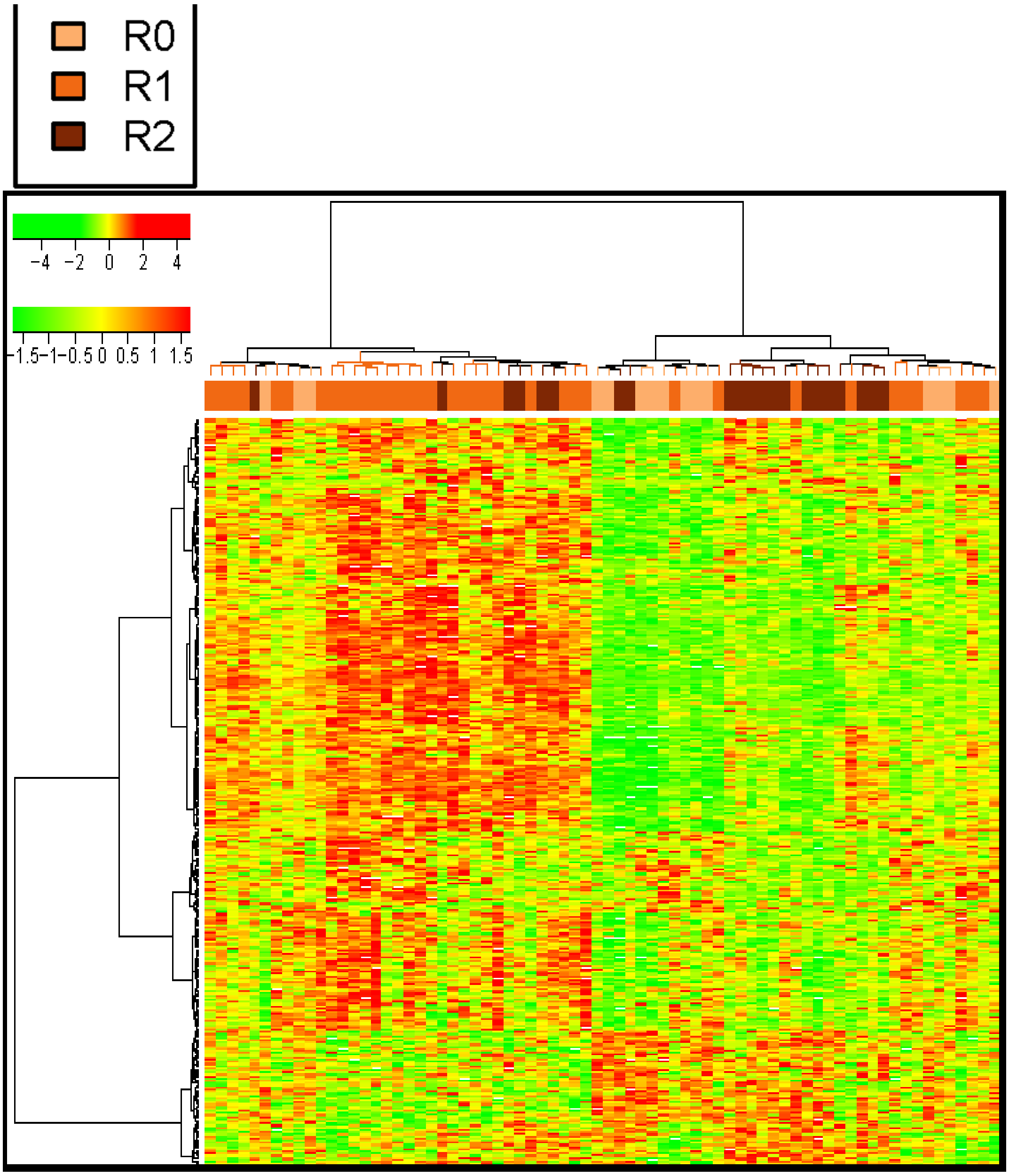


**2. Gene Ontology analysis** was performed using annotated genes selected in Welch (p<0.001) for the difference between R0 *versus* R1/R2 tumors (R0<1cm, 5cm>R1>1cm, R2>5cm).

| Gene Ontology class | p-value |
|---|---|
| GO:35107: appendage morphogenesis | 0.000183 |
| GO:35108: limb morphogenesis | 0.000183 |
| GO:30326: embryonic limb morphogenesis | 0.000112 |
| GO:35113: embryonic appendage morphogenesis | 0.000112 |
| GO:48598: embryonic morphogenesis | 0.000434 |
| GO:43009: embryonic development (sensu Vertebrata) | 0.000896 |
| GO:1701: embryonic development (sensu Mammalia) | 0.000896 |
| GO:48637: skeletal muscle development | 0.000666 |
| GO:48741: skeletal muscle fiber development | 0.000666 |
| GO:48747: muscle fiber development | 0.000666 |

| | |
|---|---|
| GO:48736: appendage development | 0.000183 |

**3. Signaling pathways** (according to Biocarta repository) with significantly changed gene expression between R0 *versus* R1/R2 tumors (R0<1cm, 5cm>R1>1cm, R2>5cm).
Least square (LS) and Kolomogorov-Smirnoff (KS) tests were used for analysis of annotated genes selected in Welch (p<0.001) for the difference between R0 *versus* R1/R2 tumors.

| | **Signaling pathways (Biocarta)** | **Signaling pathway name** | **p-value LS** | **p-value KS** |
|---|---|---|---|---|
| 1 | h_hSWI-SNFpathway | Chromatin Remodeling by hSWI/SNF ATP-dependent Complexes | 1e-05 | 0.0003721 |
| 2 | h_iresPathway | Internal Ribosome entry pathway | 1e-05 | 0.0124445 |
| 3 | h_p35alzheimersPathway | Deregulation of CDK5 in Alzheimers Disease | 1e-05 | 0.0224286 |
| 4 | h_rarrxrPathway | Nuclear receptors coordinate the activities of chromatin remodeling complexes and coactivators to facilitate initiation of transcription in carcinoma cells | 1e-05 | 0.0061562 |
| 5 | h_il2rbPathway | IL-2 Receptor Beta Chain in T cell Activation | 0.0001047 | 0.0023371 |
| 6 | h_vdrPathway | Control of Gene Expression by Vitamin D Receptor | 0.0003917 | 8e-04 |
| 7 | h_carm-erPathway | CARM1 and Regulation of the Estrogen Receptor | 0.0009617 | 0.0744877 |
| 8 | h_aktPathway | AKT Signaling Pathway | 0.0011545 | 0.0018995 |
| 9 | h_HivnefPathway | HIV-I Nef: negative effector of Fas and TNF | 0.0012251 | 0.0041861 |
| 10 | h_aifPathway | Opposing roles of AIF in Apoptosis and Cell Survival | 0.0018552 | 0.0430208 |
| 11 | h_longevityPathway | The IGF-1 Receptor and Longevity | 0.0028867 | 0.0004183 |
| 12 | h_RELAPathway | Acetylation and Deacetylation of RelA in The Nucleus | 0.0172831 | 0.0022354 |
| 13 | h_cdc25Pathway | cdc25 and chk1 Regulatory Pathway in response to DNA damage | 0.0888813 | 0.0027169 |
| 14 | h_rbPathway | RB Tumor Suppressor/Checkpoint Signaling in response to DNA damage | 0.0980191 | 0.0036151 |
| 15 | h_igf1mtorpathway | Skeletal muscle hypertrophy is regulated via AKT/mTOR pathway | 0.1458465 | 0.0042863 |

**4. Signaling pathways** (according to Biocarta repository) with significantly changed gene expression between R0 *versus* R1/R2 tumors (R0<1cm, 5cm>R1>1cm, R2>5cm). Hotelling test was used for analysis of annotated genes selected in Welch test (p<0.001) for the difference between R0 *versus* R1/R2 tumors.

| | **Signaling pathways (Biocarta)** | **Signaling pathway name** | **p-value** |
|---|---|---|---|
| 1 | h_iresPathway | Internal Ribosome entry pathway | 0.0001336 |
| 2 | h_nfkbPathway | NF-kB Signaling Pathway | 0.0004274 |
| 3 | h_il4Pathway | IL 4 signaling pathway | 0.0007516 |
| 4 | h_tercPathway | Overview of telomerase RNA component gene hTerc Transcriptional Regulation | 0.0007812 |
| 5 | h_HivnefPathway | HIV-I Nef: negative effector of Fas and TNF | 0.0009426 |
| 6 | h_RELAPathway | Acetylation and Deacetylation of RelA in The Nucleus | 0.0011095 |
| 7 | h_rhoPathway | Rho cell motility signaling pathway | 0.0013813 |
| 8 | h_calcineurinPathway | Effects of calcineurin in Keratinocyte Differentiation | 0.0014919 |
| 9 | h_rarrxrPathway | Nuclear receptors coordinate the activities of chromatin remodeling complexes and coactivators to facilitate initiation of transcription in carcinoma cells | 0.0016827 |
| 10 | h_caspasePathway | Caspase Cascade in Apoptosis | 0.0017188 |
| 11 | h_smPathway | Spliceosomal Assembly | 0.001745 |
| 12 | h_bcellsurvivalPathway | B Cell Survival Pathway | 0.0023689 |
| 13 | h_rasPathway | Ras Signaling Pathway | 0.0025734 |
| 14 | h_aktPathway | AKT Signaling Pathway | 0.0036686 |
| 15 | h_ranMSpathway | Role of Ran in mitotic spindle regulation | 0.0037095 |
| 16 | h_aifPathway | Opposing roles of AIF in Apoptosis and Cell Survival | 0.0040409 |
| 17 | h_hSWI-SNFpathway | Chromatin Remodeling by hSWI/SNF ATP-dependent Complexes | 0.0041561 |
| 18 | h_trkaPathway | Trka Receptor Signaling Pathway | 0.004251 |
| 19 | h_rnaPathway | Double Stranded RNA Induced Gene Expression | 0.004562 |
| 20 | h_deathPathway | Induction of apoptosis through DR3 and DR4/5 Death Receptors | 0.0046078 |
| 21 | h_chemicalPathway | Apoptotic Signaling in Response to DNA Damage | 0.0046144 |
| 22 | h_vdrPathway | Control of Gene Expression by Vitamin D Receptor | 0.0047563 |
| 23 | h_p35alzheimersPathway | Deregulation of CDK5 in Alzheimers Disease | 0.0048028 |
| 24 | h_glycolysisPathway | Glycolysis Pathway | 0.0048048 |
