# Supplementary material for: Gene Expression Analysis in Ovarian Cancer – Faults and Hints from DNA Microarray Study
Source: Front Oncol. 2014 Jan 28;4:6. doi: 10.3389/fonc.2014.00006 (PMC3904181; doi:10.3389/fonc.2014.00006)
Supplement: Supplementary file 10 [file 61993_Lisowska_DataSheet10.PDF]

Supplementary table 6. Chemotherapy response

List of genes with significantly changed expression in tumors responding and non-responding to chemotherapy in relation to the residual tumor size; 196 probe sets with p<0.001 were selected in Welch test for comparison of tumors from patients with complete response (CR) or partial response (PR) after first line chemotherapy versus stable disease (SD) and progression (P).

Affymetrix probe set ID	p-value	Gene symbol	Gene name
221193_s_at	5,23E-11	ZCCHC10	zinc finger, CCHC domain containing 10
241902_at	3,02E-07	C10orf48	chromosome 10 open reading frame 48
228231_at	1,03E-06	SNX8	Sorting nexin 8
205350_at	1,11E-06	CRABP1	cellular retinoic acid binding protein 1
231181_at	2,87E-06		Transcribed locus
1553392_at	3,22E-06	FLJ25818 ; LOC441279 ; LOC442615	hypothetical protein FLJ25818 ; similar to hypothetical protein FLJ25818 ; similar to hypothetical protein FLJ25818
234480_at	3,85E-06	DLG2	Discs, large homolog 2, chapsyn-110 (Drosophila)
1560819_a_at	4,25E-06		Hypothetical gene supported by BC040060
240067_at	5,06E-06	FGF12	Fibroblast growth factor 12
239187_at	1,07E-05	FLJ20280	hypothetical protein FLJ20280
216025_x_at	1,19E-05	CYP2C19 ; CYP2C9	cytochrome P450, family 2, subfamily C, polypeptide 19 ; cytochrome P450, family 2, subfamily C, polypeptide 9
230673_at	1,43E-05	PKHD1L1	polycystic kidney and hepatic disease 1 (autosomal recessive)-like 1
210398_x_at	1,48E-05	FUT6	fucosyltransferase 6 (alpha (1,3) fucosyltransferase)
224337_s_at	1,72E-05	FZD4	frizzled homolog 4 (Drosophila) ; frizzled homolog 4 (Drosophila)
1557676_at	1,81E-05		CDNA clone IMAGE:4817003
204938_s_at	2,04E-05	PLN	phospholamban
225223_at	2,65E-05	SMAD5	SMAD, mothers against DPP homolog 5 (Drosophila)
219231_at	2,74E-05	NCOA6IP	nuclear receptor coactivator 6 interacting protein
230680_at	3,35E-05		Transcribed locus
209307_at	3,60E-05	SWAP70	SWAP-70 protein
214131_at	3,86E-05	CYorf15B	chromosome Y open reading frame 15B
205054_at	3,98E-05	NEB	nebulin
1564795_at	4,13E-05	NUP98	Nucleoporin 98kDa
237160_at	4,22E-05	MGC34732	hypothetical protein MGC34732
217941_s_at	4,31E-05	ERBB2IP	erbb2 interacting protein
221467_at	4,51E-05	MC4R	melanocortin 4 receptor
237281_at	4,73E-05	AKAP14	A kinase (PRKA) anchor protein 14
243855_at	4,99E-05	SNRPN	Small nuclear ribonucleoprotein polypeptide N
236407_at	5,35E-05	KCNE1	potassium voltage-gated channel, Isk-related family, member 1
236320_at	5,40E-05	CCDC17	coiled-coil domain containing 17
206814_at	5,60E-05	NGFB	nerve growth factor, beta polypeptide
220624_s_at	5,83E-05	ELF5	E74-like factor 5 (ets domain transcription factor)
239098_at	5,95E-05	KCNRG	potassium channel regulator
212597_s_at	7,31E-05	HMG2L1	high-mobility group protein 2-like 1
237225_at	7,63E-05	ZFY	Zinc finger protein, Y-linked
205825_at	8,08E-05	PCSK1	proprotein convertase subtilisin/kexin type 1
226755_at	8,52E-05		Nasopharyngeal carcinoma-associated antigen NPC-A-5
232996_at	8,66E-05	JM11	JM11 protein
235884_at	9,25E-05		Transcribed locus, weakly similar to XP_376619.2 PREDICTED: hypothetical protein XP_376619 [Homo sapiens]
1566831_at	9,85E-05	LOC283104	hypothetical protein LOC283104
241886_x_at	1,04E-04	LOC440115	LOC440115
234162_at	1,09E-04	DJ159A19.3	AT hook, DNA binding motif, containing 1
1553183_at	1,11E-04	UMODL1	uromodulin-like 1
211875_x_at	1,16E-04	PCDHGA10	protocadherin gamma subfamily A, 10
235156_at	1,39E-04	BRWD3	Bromodomain and WD repeat domain containing 3
1568939_at	1,43E-04	OR8B2	Olfactory receptor, family 8, subfamily B, member 2
1563169_at	1,45E-04	POU6F2	POU domain, class 6, transcription factor 2
205509_at	1,52E-04	CPB1	carboxypeptidase B1 (tissue)
1564573_at	1,56E-04	LOC402778	similar to RIKEN cDNA 6330512M04 gene (mouse)
221371_at	1,65E-04	TNFSF18	tumor necrosis factor (ligand) superfamily, member 18
1553794_at	1,66E-04	STOML3	stomatin (EPB72)-like 3

204952_at	1,69E-04	LYPD3	LY6/PLAUR domain containing 3
202034_x_at	1,69E-04	RB1CC1	RB1-inducible coiled-coil 1
208043_at	1,70E-04		
228961_at	1,72E-04	FLJ35954	hypothetical protein FLJ35954
231017_at	1,77E-04	STK11	serine/threonine kinase 11 (Peutz-Jeghers syndrome)
1555942_a_at	1,79E-04		Nasopharyngeal carcinoma-associated antigen NPC-A-5
243056_at	1,79E-04	MGC47869	Hypothetical protein MGC47869
222790_s_at	1,81E-04	RSBN1	round spermatid basic protein 1
1553944_at	1,81E-04	MIA2	melanoma inhibitory activity 2
206479_at	1,87E-04	TRPM1	transient receptor potential cation channel, subfamily M, member 1
239319_at	1,87E-04		CDNA clone IMAGE:5297581
228220_at	1,90E-04	FCHO2	FCH domain only 2
1565694_at	1,92E-04	DTYMK	deoxythymidylate kinase (thymidylate kinase)
212122_at	2,00E-04	RHOQ ; LOC284988	ras homolog gene family, member Q ; similar to ARHQ protein
214340_at	2,23E-04	ALOX12P2	arachidonate 12-lipoxygenase pseudogene 2
233229_at	2,38E-04	SCFD1	sec1 family domain containing 1
207815_at	2,39E-04	PF4V1	platelet factor 4 variant 1
235591_at	2,41E-04	SSTR1	somatostatin receptor 1
1568606_at	2,47E-04	FLJ46266	FLJ46266 protein
239430_at	2,50E-04	IGFL1	insulin growth factor-like family member 1
215989_at	2,55E-04	CBX2	chromobox homolog 2 (Pc class homolog, Drosophila)
1564482_at	2,56E-04	ATP5O	ATP synthase, H+ transporting, mitochondrial F1 complex, O subunit (oligomycin sensitivity conferring protein)
205628_at	2,57E-04	PRIM2A	primase, polypeptide 2A, 58kDa
231373_at	2,58E-04	RNF133	ring finger protein 133
234803_at	2,58E-04	CSTL1	cystatin-like 1
1570339_x_at	2,59E-04	MGC25181	Hypothetical protein MGC25181 ; CDNA FLJ26792 fis, clone PRS04865
226441_at	2,74E-04	MAP3K2	Mitogen-activated protein kinase kinase kinase 2
1555187_at	2,92E-04		
215829_at	2,97E-04	SHANK2	SH3 and multiple ankyrin repeat domains 2
215968_at	3,02E-04	MGC15606	Family with sequence similarity 55, member C
1556003_a_at	3,08E-04		
1559488_at	3,13E-04	LOC257358	hypothetical protein LOC257358
238094_at	3,14E-04		Homo sapiens, clone IMAGE:4523945, mRNA
242345_at	3,24E-04	LOC285929	similar to matrilin 2 precursor
224103_at	3,25E-04		Clone FLB1825 PRO0419
1565769_at	3,26E-04	ZNF268	Zinc finger protein 268
220108_at	3,38E-04	GNA14	guanine nucleotide binding protein (G protein), alpha 14
237592_at	3,43E-04		
231143_at	3,58E-04		Similar to RIKEN cDNA 2810417M05
240809_at	3,62E-04	C21orf121	chromosome 21 open reading frame 121
228644_s_at	3,62E-04	SLC12A4	Solute carrier family 12 (potassium/chloride transporters), member 4
210735_s_at	3,68E-04	CA12	carbonic anhydrase XII
232771_at	3,72E-04	NRK	Nik related kinase
201652_at	3,78E-04	COPS5	COP9 constitutive photomorphogenic homolog subunit 5 (Arabidopsis)
224367_at	3,82E-04	BEX2	brain expressed X-linked 2 ; brain expressed X-linked 2
242763_at	3,90E-04	PKHD1L1	Polycystic kidney and hepatic disease 1 (autosomal recessive)-like 1
242263_at	3,91E-04	TMED5	Transmembrane emp24 protein transport domain containing 5
210533_at	3,98E-04	MSH4	mutS homolog 4 (E. coli)
233348_at	4,09E-04	KIAA1423	KIAA1423
238720_at	4,11E-04		
231550_at	4,16E-04	XYLT2	Xylosyltransferase II
229804_x_at	4,20E-04	CBWD1 ; CBWD2 ; CBWD3	COBW domain containing 1 ; COBW domain containing 2 ; COBW domain containing 3
228745_at	4,21E-04	FLJ13611	Hypothetical protein FLJ13611
227288_at	4,26E-04		Hypothetical LOC133993
218611_at	4,31E-04	IER5	immediate early response 5

208096_s_at	4,32E-04	COL21A1	collagen, type XXI, alpha 1 ; collagen, type XXI, alpha 1
207307_at	4,55E-04	HTR2C	5-hydroxytryptamine (serotonin) receptor 2C
220433_at	4,63E-04	PRRG3	proline rich Gla (G-carboxyglutamic acid) 3 (transmembrane)
209951_s_at	4,64E-04	MAP2K7	mitogen-activated protein kinase kinase 7
218665_at	4,73E-04	FZD4	frizzled homolog 4 (Drosophila)
236469_at	4,77E-04		MRNA full length insert cDNA clone EUROIMAGE 110216
236211_at	4,82E-04		Transcribed locus, weakly similar to NP_009083.1 zinc finger protein 195 [Homo sapiens]
222161_at	4,85E-04	NAALAD2	N-acetylated alpha-linked acidic dipeptidase 2
212672_at	4,89E-04	ATM	ataxia telangiectasia mutated (includes complementation groups A, C and D)
231337_at	5,01E-04		
236761_at	5,03E-04	LHFPL3	lipoma HMGIC fusion partner-like 3
238822_at	5,13E-04	MRPL3	Mitochondrial ribosomal protein L3
1553912_at	5,14E-04	FLJ35424	hypothetical protein FLJ35424
228871_at	5,17E-04		
217541_x_at	5,18E-04	LOC125893	hypothetical protein LOC125893
234233_s_at	5,26E-04	KBTBD2	kelch repeat and BTB (POZ) domain containing 2
237282_s_at	5,29E-04	AKAP14	A kinase (PRKA) anchor protein 14
209484_s_at	5,40E-04	C1orf48	chromosome 1 open reading frame 48
233934_at	5,43E-04		Clone N1 NTera2D1 teratocarcinoma mRNA
1557829_at	5,47E-04	SLC8A1	Solute carrier family 8 (sodium/calcium exchanger), member 1
203074_at	5,49E-04	ANXA8	annexin A8
1559513_a_at	5,52E-04	FANCC	Fanconi anemia, complementation group C
244815_at	5,60E-04		
216849_at	5,66E-04	FLJ16124	FLJ16124 protein
235141_at	5,68E-04	MARVELD2	MARVEL domain containing 2
238559_at	5,71E-04		CDNA clone IMAGE:5266242
205434_s_at	5,74E-04	AAK1	AP2 associated kinase 1
242758_x_at	5,81E-04	JMJD1A	Jumonji domain containing 1A
224482_s_at	5,82E-04	RAB11FIP4	RAB11 family interacting protein 4 (class II) ; RAB11 family interacting protein 4 (class II)
1557729_at	5,96E-04		FP2025
216545_at	5,98E-04	LOC441886	similar to Aspartate aminotransferase, mitochondrial precursor (Transaminase A) (Glutamate oxaloacetate transaminase-2)
222172_at	5,99E-04	NPAS3	neuronal PAS domain protein 3
244261_at	6,00E-04	IL28RA	interleukin 28 receptor, alpha (interferon, lambda receptor)
239575_at	6,02E-04	TMEM10	transmembrane protein 10
237495_at	6,07E-04	MPP7	membrane protein, palmitoylated 7 (MAGUK p55 subfamily member 7)
1555380_at	6,16E-04	ADAMTS4	ADAM metallopeptidase with thrombospondin type 1 motif, 4
232636_at	6,28E-04	SLITRK4	SLIT and NTRK-like family, member 4
1552777_a_at	6,31E-04	RAET1E	retinoic acid early transcript 1E
202047_s_at	6,43E-04	CBX6	chromobox homolog 6
226821_at	6,45E-04		Full-length cDNA clone CS0DF029YD16 of Fetal brain of Homo sapiens (human)
215103_at	6,58E-04	CYP2C18	cytochrome P450, family 2, subfamily C, polypeptide 18
207032_s_at	6,60E-04	CRISP1	cysteine-rich secretory protein 1
223337_at	6,77E-04	SDCCAG10	serologically defined colon cancer antigen 10
220585_at	6,79E-04	HKDC1	hexokinase domain containing 1
226742_at	6,83E-04		Transcribed locus, moderately similar to XP_512541.1 PREDICTED: similar to hypothetical protein [Pan troglodytes]
1555471_a_at	6,90E-04	FMN2	formin 2
211442_x_at	6,94E-04	CYP3A43	cytochrome P450, family 3, subfamily A, polypeptide 43
221485_at	6,99E-04	B4GALT5	UDP-Gal:betaGlcNAc beta 1,4- galactosyltransferase, polypeptide 5
244256_at	7,11E-04	CACNA1E	Calcium channel, voltage-dependent, alpha 1E subunit
220192_x_at	7,19E-04	SPDEF	SAM pointed domain containing ets transcription factor
228377_at	7,21E-04	KLHL14	kelch-like 14 (Drosophila)
240431_at	7,24E-04	LOC144762	hypothetical LOC144762
233629_at	7,31E-04	FOXO3A	Forkhead box O3A
224864_at	7,37E-04	SRA1	steroid receptor RNA activator 1
244321_at	7,41E-04	PGAP1	GPI deacylase

209793_at	7,46E-04	GRIA1	glutamate receptor, ionotropic, AMPA 1
216738_at	7,60E-04	HRH1	Histamine receptor H1
1562380_at	7,64E-04		CDNA clone IMAGE:5268742
210619_s_at	7,78E-04	HYAL1	hyaluronoglucosaminidase 1
207415_at	8,02E-04	PLA2R1	phospholipase A2 receptor 1, 180kDa
212620_at	8,12E-04	ZNF609	zinc finger protein 609
1569129_s_at	8,13E-04		Homo sapiens, clone IMAGE:4695648, mRNA
229118_at	8,15E-04	PRRG3	Proline rich Gla (G-carboxyglutamic acid) 3 (transmembrane)
226449_at	8,18E-04	FLJ36090	hypothetical protein FLJ36090
239117_at	8,22E-04		Transcribed locus
226321_at	8,22E-04	LYSMD3	LysM, putative peptidoglycan-binding, domain containing 3
227735_s_at	8,22E-04	C10orf99	chromosome 10 open reading frame 99
219614_s_at	8,32E-04	SLC6A20	solute carrier family 6 (proline IMINO transporter), member 20
1566152_a_at	8,42E-04		CDNA FLJ39016 fis, clone NT2RP7001532
206069_s_at	8,44E-04	ACADL	acyl-Coenzyme A dehydrogenase, long chain
213040_s_at	8,46E-04	NPTXR	neuronal pentraxin receptor
233091_at	8,48E-04	ATAD3B	ATPase family, AAA domain containing 3B
214739_at	8,48E-04	LRCH3	leucine-rich repeats and calponin homology (CH) domain containing 3
238164_at	8,53E-04	USP6NL	USP6 N-terminal like
233906_at	8,81E-04		MRNA; cDNA DKFZp434K1021 (from clone DKFZp434K1021)
1567380_at	8,82E-04	LOC399818	Similar to CG9643-PA
228614_at	8,84E-04	LOC205251	hypothetical protein LOC205251
1553321_a_at	8,84E-04	SULT1C2	sulfotransferase family, cytosolic, 1C, member 2
210290_at	8,84E-04	ZNF174	zinc finger protein 174
235347_at	8,85E-04		Homo sapiens, clone IMAGE:4183899, mRNA
1570293_at	8,87E-04	TBL1X	transducin (beta)-like 1X-linked
1554379_a_at	8,89E-04	TP73	tumor protein p73
206797_at	9,10E-04	NAT2	N-acetyltransferase 2 (arylamine N-acetyltransferase)
1557406_s_at	9,17E-04	LOC283697	hypothetical protein LOC283697
244123_at	9,52E-04	DMWD	Dystrophia myotonica-containing WD repeat motif
235611_at	9,68E-04		Transcribed locus
215704_at	9,86E-04	FLG	filaggrin
239606_at	9,92E-04	GCNT2	Glucosaminyl (N-acetyl) transferase 2, I-branching enzyme
207039_at	9,92E-04	CDKN2A	cyclin-dependent kinase inhibitor 2A (melanoma, p16, inhibits CDK4)
238087_at	9,92E-04		Transcribed locus, weakly similar to XP_524454.1 PREDICTED: hypothetical protein XP_524454 [Pan troglodytes]
