# Supplementary material for: Gene Expression Analysis in Ovarian Cancer – Faults and Hints from DNA Microarray Study
Source: Front Oncol. 2014 Jan 28;4:6. doi: 10.3389/fonc.2014.00006 (PMC3904181; doi:10.3389/fonc.2014.00006)
Supplement: Supplementary file 11 [file 61993_Lisowska_DataSheet11.PDF]

Response to chemotherapy

1. Hierarchical clustering: Dendrogram showes the relations between tumor samples from patients with different response to chemotherapy. Heat map shows relative intensity of genes expression. Interestingly, samples from patients with stable disease (SD) and progression (P) are clustered together and show homogenous gene expression pattern, distinct from samples from patients with complete response (CR) or partial response (PR).

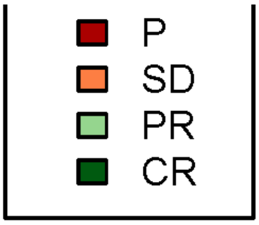

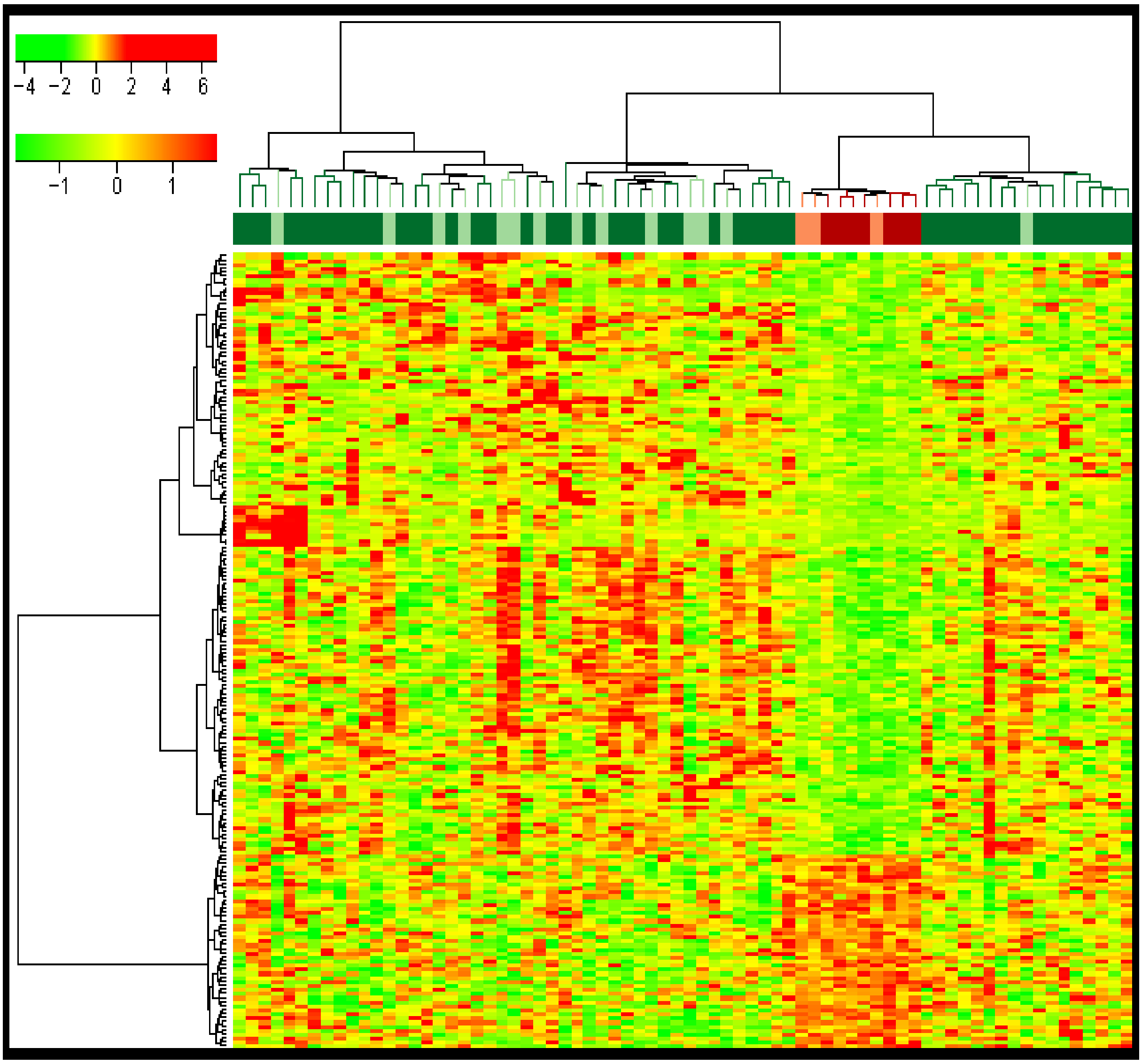

2. Gene Ontology analysis was performed using annotated genes selected in Welch test (p<0.001) for the difference between CR+PR versus SD+P tumors.

**Gene Ontology class**	**p-value**
GO:7402: ganglion mother cell fate determination	0.0061
GO:51327: M phase of meiotic cell cycle	0.00686
GO:7126: meiosis	0.00686

GO:51321: meiotic cell cycle	0.00686
GO:7631: feeding behavior	0.00515
GO:7222: frizzled signaling pathway	0.00876

3. Signaling pathways (according to Biocarta repository) with significantly changed gene expression between CR+PR versus SD+P tumors. Least square (LS) and Kolomogorov-Smirnoff (KS) tests were used for analysis of annotated genes selected in Welch (p<0.001) for the difference between CR+PR versus SD+P tumors.

	**Signaling pathways (Biocarta)**	**Signaling pathway name**	**p-value LS**	**p-value KS**
1	h_cellcyclePathway	Cyclins and Cell Cycle Regulation	0.0034352	0.0664212
2	h_botulinPathway	Blockade of Neurotransmitter Relase by Botulinum Toxin	0.0088157	0.0001285
