# Supplementary material for: Gene Expression Analysis in Ovarian Cancer – Faults and Hints from DNA Microarray Study
Source: Front Oncol. 2014 Jan 28;4:6. doi: 10.3389/fonc.2014.00006 (PMC3904181; doi:10.3389/fonc.2014.00006)
Supplement: Supplementary file 12 [file 61993_Lisowska_DataSheet12.PDF]

Supplementary table 8. Disease free survival

Genes (18 probe sets) related with disease free survival (DFS) were selected using Cox regression model. Odds ratios (OR) for shortening of DFS were estimated for twofold expression change of each gene.

	Affymetrix probe set ID	Gene description	Gene name	p-value	FDR	OR
1	240939_x_at	Cytoplasmic linker associated protein 1	CLASP1	0.0002351	0.8571438	3.529
2	205536_at	vav 2 oncogene	VAV2	0.0003277	0.8571438	3.712
3	218061_at	male-enhanced antigen 1	MEA1	0.0004064	0.8571438	0.32
4	212760_at	ubiquitin protein ligase E3 component n-recognin 2	UBR2	0.0004656	0.8571438	0.331
5	218106_s_at	mitochondrial ribosomal protein S10	MRPS10	0.000487	0.8571438	0.398
6	227704_at	chromosome 19 open reading frame 12	C19orf12	0.0004995	0.8571438	2.318
7	1559026_at	CDNA clone IMAGE:5295490		0.0005027	0.8571438	0.436
8	228658_at	chromosome 22 open reading frame 35	C22orf35	0.0005542	0.8571438	2.508
9	211022_s_at	alpha thalassemia/mental retardation syndrome X-linked (RAD54 homolog, S. cerevisiae)	ATRX	0.0006658	0.8571438	3.135
10	1552736_a_at	neuropilin (NRP) and tolloid (TLL)-like 1	NETO1	0.0006761	0.8571438	1.968
11	214721_x_at	CDC42 effector protein (Rho GTPase binding) 4	CDC42EP4	0.0006924	0.8571438	0.41
12	242645_at	Mitochondrial carrier homolog 2 (C. elegans)	MTCH2	0.0007589	0.8571438	3.06
13	222304_x_at	olfactory receptor, family 7, subfamily E, member 47 pseudogene	OR7E47P	0.0008108	0.8571438	0.312
14	213188_s_at	MYC induced nuclear antigen	MINA	0.0008254	0.8571438	0.345
15	206326_at	gastrin-releasing peptide	GRP	0.0008337	0.8571438	2.342
16	218062_x_at	CDC42 effector protein (Rho GTPase binding) 4	CDC42EP4	0.0008715	0.8571438	0.392
17	209317_at	polymerase (RNA) I polypeptide C, 30kDa	POLR1C	0.0008818	0.8571438	0.387
18	91952_at	hypothetical protein BC002926	LOC90379	0.0009914	0.8571438	2.982
